# Molybdate‐Modified NiOOH for Efficient Methanol‐Assisted Seawater Electrolysis

**DOI:** 10.1002/advs.202410911

**Published:** 2025-02-19

**Authors:** Zhen Li, Youbin Zheng, Wenhan Zu, Liang Dong, Lawrence Yoon Suk Lee

**Affiliations:** ^1^ Department of Applied Biology and Chemical Technology and Research Institute for Smart Energy The Hong Kong Polytechnic University Hung Hom Kowloon Hong Kong SAR China; ^2^ Key Laboratory of Dielectric and Electrolyte Functional Material School of Resources and Materials Northeastern University at Qinhuangdao Qinhuangdao Hebei 066004 China

**Keywords:** anti‐corrosion, direct seawater electrolysis, methanol electrooxidation, molybdate modulation, non‐electrochemical process

## Abstract

Seawater electrolysis holds great promise for sustainable, green hydrogen production but faces challenges of high overpotentials and competing chlorine evolution reaction (CER). Replacing the oxygen evolution reaction with the methanol oxidation reaction (MOR) presents a compelling alternative due to its lower anodic potential which mitigates the risk of CER. While NiOOH is known for its MOR activity, its performance is limited by sluggish non‐electrochemical kinetics and Cl‐induced degradation. Herein, a MoO_4_^2−^‐modified NiOOH electrocatalyst is reported that significantly enhances MOR‐assisted seawater splitting efficiency. In situ leached MoO_4_^2−^ from the heterojunction optimizes methanol adsorption and facilitates proton migration, thereby accelerating the non‐electrochemical steps in MOR. Additionally, the adsorbed MoO_4_^2−^ effectively repels Cl^−^, protecting the electrodes from Cl^−^‐induced corrosion. The MOR‐assisted electrolyzer using NiMo||Ni(OH)_2_/NiMoO₄ requires only 1.312 V to achieve 10 mA cm^−2^, substantially lower than conventional alkaline seawater electrolysis (1.576 V). Furthermore, it demonstrates remarkable stability, sustaining high current densities (up to 1.0 A cm^−2^) for over 130 h. This work presents a promising strategy for designing high‐performance electrocatalysts for efficient and sustainable green hydrogen production from seawater.

## Graphical abstract

MoO_4_^2−^‐modulated NiOOH is synthesized and employed as multifunctional electrocatalysts for methanol‐assisted direct seawater electrolysis. The MoO_4_^2−^ modification enhances the oxygen evolution reaction and proton‐coupled electron transfer in the methanol oxidation reaction while simultaneously increasing the energy barrier for the chlorine evolution reaction. This dual effect effectively reduces the energy requirements of seawater splitting and enables stable operation at industrial current densities.

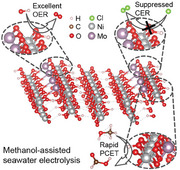

## 1 Introduction

Green hydrogen production via water electrolysis powered by renewable energy is a key step toward carbon neutrality.^[^ ^1^, ^2^ ^]^ While freshwater‐based electrolyzers offer a promising solution, their large‐scale deployment raises concerns about water scarcity. Seawater, comprising 96.5% of Earth's water, presents a compelling alternative for green hydrogen generation through direct electrolysis, aligning with dual‐carbon goals.^[^ ^3^, ^4^, ^5^ ^]^ This approach eliminates the need for desalination, simplifying the process and reducing production costs. Nevertheless, direct seawater electrolysis faces challenges due to the high thermodynamic energy barrier for water oxidation (1.23 V vs reversible hydrogen electrode (RHE)) and the corrosive effects of Cl^−^‐rich environments on electrocatalysts.^[^ ^6^, ^7^ ^]^

Alternative anodic reactions to the sluggish oxygen evolution reaction (OER) have recently been explored to reduce the required potential of electrolysis cells in both freshwater and seawater.^[^ ^8^, ^9^, ^10^, ^11^, ^12^ ^]^ Methanol, due to its low cost and favorable thermodynamic oxidation potential (0.016 V vs RHE), is a promising candidate for small‐molecule‐assisted seawater splitting.^[^ ^13^ ^]^ Beyond Pt‐based catalysts such as Pt_1.8_Pd_0.2_CuGa and PtTe,^[^ ^14^, ^15^ ^]^ NiOOH, formed in situ during the surface reconstruction of Ni‐based electrocatalysts, is considered the critical active species for methanol oxidation reaction (MOR).^[^ ^16^, ^17^, ^18^ ^]^ High‐valence Ni^3+^ facilitates proton‐coupled electron transfer (PCET) by capturing protons from methanol.^[^ ^16^, ^19^ ^]^ Thus, efficient Ni^3+^ utilization and MOR activity hinge on this non‐electrochemical Ni^3+^–methanol interaction. Recent studies suggest that residual or adsorbed anionic species can modulate the electronic structure of NiOOH active sites, thereby optimizing anodic oxidation activities.^[^ ^20^, ^21^, ^22^, ^23^ ^]^ However, their influence on MOR, particularly the non‐electrochemical process, remains largely unexplored. In seawater, Cl^−^ ions readily attack electron‐deficient transition metal sites, such as Ni^3+^, triggering the chlorine evolution reaction (CER) and generating corrosive products (Cl_2_ or ClO^−^) that deactivate catalysts. Limiting the anodic potential below 1.72 V in alkaline media can suppress CER but usually results in significantly lower current densities than industrial standards.^[^ ^24^, ^25^ ^]^ Recent studies indicate that manipulating the catalyst surface microenvironment through anion adsorption can reduce Cl^−^ adsorption, thus effectively mitigating corrosion by chlorine derivatives and ensuring the stability of seawater oxidation.^[^ ^26^, ^27^, ^28^, ^29^ ^]^ The key challenge lies in simultaneously accelerating the non‐electrochemical process in MOR and alleviating chlorine corrosion during methanol oxidation‐assisted seawater electrolysis.

Herein, we present a MoO_4_^2−^‐adsorption strategy to modulate the surface microenvironment of NiOOH for efficient MOR‐assisted seawater splitting. This approach simultaneously adjusts Cl^−^ adsorption behavior and promotes the non‐electrochemical process in MOR. The pre‐catalyst, Ni(OH)_2_/NiMoO_4_ (Ni(OH)_2_/NMO), possesses a 3D hierarchical structure that effectively enlarges the electrochemical surface area for efficient mass diffusion. The heterojunction between Ni(OH)_2_ and NiMoO_4_ facilitates the reconstruction process of Ni(OH)_2_ to generate NiOOH, which serves as the active site for both MOR and OER. In situ leached MoO_4_^2−^ optimizes the coordination environment on the NiOOH surface to facilitate proton capture from methanol, thus promoting PCET during MOR. Additionally, the surface‐coordinated MoO_4_^2−^ increases the Cl^−^ adsorption energy, thereby mitigating catalyst corrosion. As a result, the Ni(OH)_2_/NMO demonstrates exceptional electrocatalytic MOR performance, achieving a current density of 10 mA cm^−2^ at a low potential of 1.305 V in alkaline methanol. In alkaline seawater, Ni(OH)_2_/NMO requires low overpotentials of 267 and 381 mV to reach 100 and 500 mA cm^−2^, respectively, and maintains excellent stability at 500 mA cm^−2^ for 600 h. Moreover, a membrane electrode assembly electrolyzer based on NiMo||Ni(OH)_2_/NMO exhibits 13.5% energy savings at 0.5 A cm^−2^ in a hybrid electrolyte of alkaline seawater and methanol compared to a commercial Pt/C||RuO_2_ electrolyzer. This work presents a promising strategy for developing small molecule‐assisted seawater electrolysis for sustainable green hydrogen production.

## 2 Results and Discussion

### 2.1 Synthesis and Characterizations of Ni(OH)_2_/NMO

**Figure 1:**
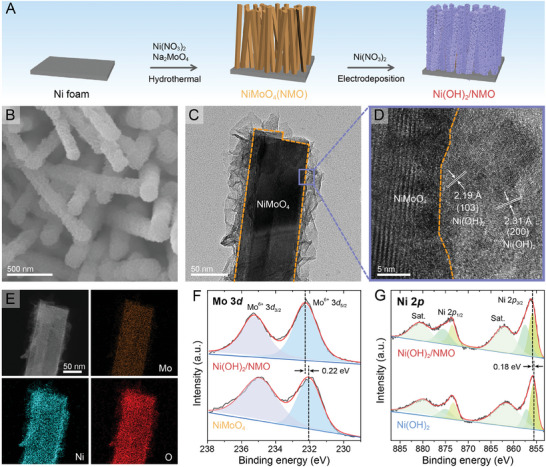
A) A schematic diagram of the synthetic procedure for Ni(OH)_2_/NMO. B) SEM, C) TEM, D) high‐resolution TEM, and E) STEM and the corresponding EDS mapping images of Ni(OH)_2_/NMO. XPS spectra of F) Ni(OH)_2_/NMO and NiMoO_4_ and G) Ni(OH)_2_/NMO and Ni(OH)_2_ in the Mo 3*d* and Ni 2*p* regions, respectively.

**Figure** **1**A depicts a two‐step route for synthesizing hierarchical Ni(OH)_2_/NMO on Ni foam. First, NiMoO_4_ nanorods (average *d* = 110 nm, Figure S1a, Supporting Information) were grown on Ni foam using a hydrothermal method in a mixed solution of Ni(NO_3_)_2_ and Ni_2_MoO_4_ (details in Supporting Information). Subsequent electroreduction of Ni(NO_3_)_2_ at −1 V (vs saturated calomel electrode, SCE) forms Ni(OH)_2_ nanosheets on the NiMoO_4_ nanorods (Figure 1B). This approach enables precise control over the Ni(OH)_2_‐to‐NMO ratio and Ni(OH)_2_ morphology (Figures S1b,c and Table S1, Supporting Information). Transmission electron microscopic (TEM) image displays the hierarchical morphology of Ni(OH)_2_/NMO (Figure 1C), confirming the nanorod structure of NiMoO_4_ remaining intact after Ni(OH)_2_ nanosheet decoration. The high‐resolution TEM image, presented in Figure 1D, reveals two lattice spacings of 2.19 and 2.31 Å, corresponding to the (103) and (200) planes of Ni(OH)_2_, respectively. Scanning TEM (STEM) analysis of Ni(OH)_2_/NMO along with the corresponding energy‐dispersive spectroscopic (EDS) mappings reveal Mo enrichment within the inner nanorod region, while Ni and O are uniformly distributed throughout the entire structure (Figure 1E).

X‐ray diffraction (XRD) analysis of the pristine NiMoO_4_ nanorods confirms that its structure matches the reference pattern for hydrated NiMoO_4_ (JCPDS 13–0128) and is well maintained after the electrodeposition process (Figure S2, Supporting Information). Raman spectroscopic analysis further supports the successful deposition of Ni(OH)_2_ on NiMoO_4_ nanorods (Figure S3, Supporting Information). Characteristic Raman peaks for NiMoO_4_ at 347 cm^−1^ (Mo–O bending), 825 and 857 cm^−1^ (asymmetric Mo═O stretching), and 941 cm^−1^ (symmetric Mo═O stretching)^[^ ^30^ ^]^ are observed alongside peaks corresponding to Ni(OH)_2_ at 460 and 1045 cm^−1^.^[^ ^31^ ^]^

X‐ray photoelectron spectroscopy (XPS) was employed to investigate the changes in chemical composition and surface electronic states. The high‐resolution Mo 3*d* spectrum of pristine NiMoO_4_ nanorod displays two peaks at 232.05 and 235.15 eV, which can be ascribed to Mo 3*d*_5/2_ and Mo 3*d*_3/2_, respectively (Figure 1F). These peaks are separated by 3.10 eV, indicating the Mo^6+^ oxidation state.^[^ ^32^ ^]^ In Ni(OH)_2_/NMO, the Mo 3*d* peaks positively shift by 0.22 eV compared to pristine NiMoO_4_, suggesting a reduction in electron density around the Mo sites. This can be attributed to charge migration at the interface between Ni(OH)_2_ and NiMoO_4_.^[^ ^33^ ^]^ The Ni 2*p* spectra of both Ni(OH)_2_ and Ni(OH)_2_/NMO exhibit two pairs of Ni 2*p*_3/2_ and Ni 2*p*_1/2_ peaks corresponding to Ni^2+^ and Ni^3+^ species (Figure 1G). Additionally, satellite peaks associated with Ni^2+^ species are observed.^[^ ^34^, ^35^ ^]^ Similar to the Mo 3*d* peaks, a positive shift of 0.18 eV is observed in the Ni 2*p* region of Ni(OH)_2_/NMO. This indicates that Ni atoms in Ni(OH)_2_/NMO are in a higher valence state due to electronic interplay with NiMoO_4_. This is further supported by an increase in the Ni^3+^/(Ni^3+^+Ni^2+^) peak area ratio from 0.31 in Ni(OH)_2_ to 0.48 in Ni(OH)_2_/NMO (Figure S4, Supporting Information). These observations signify a higher proportion of Ni^3+^ species within the composite, which is known to promote the formation of NiOOH, the catalytically active species for OER.^[^ ^28^ ^]^ The O 1*s* spectra of Ni(OH)_2_, NiMoO_4_, and Ni(OH)_2_/NMO can be deconvoluted into three peaks of metal–oxygen (M═O) bond, hydroxyl group, and adsorbed water molecules (Figure S5, Supporting Information).^[^ ^36^, ^37^, ^38^ ^]^ The M_1_═O and M_2_═O peaks in Ni(OH)_2_ and NiMoO_4_ are observed at 531.2 and 530.35 eV, respectively. In Ni(OH)_2_/NMO, these two peaks exhibit negative shifts of 0.2 and 0.35 eV, respectively, indicating an increase in electron density around the O sites.

### 2.2 OER Performances of Ni(OH)2/NMO in Freshwater and Alkaline Seawater

**Figure 2:**
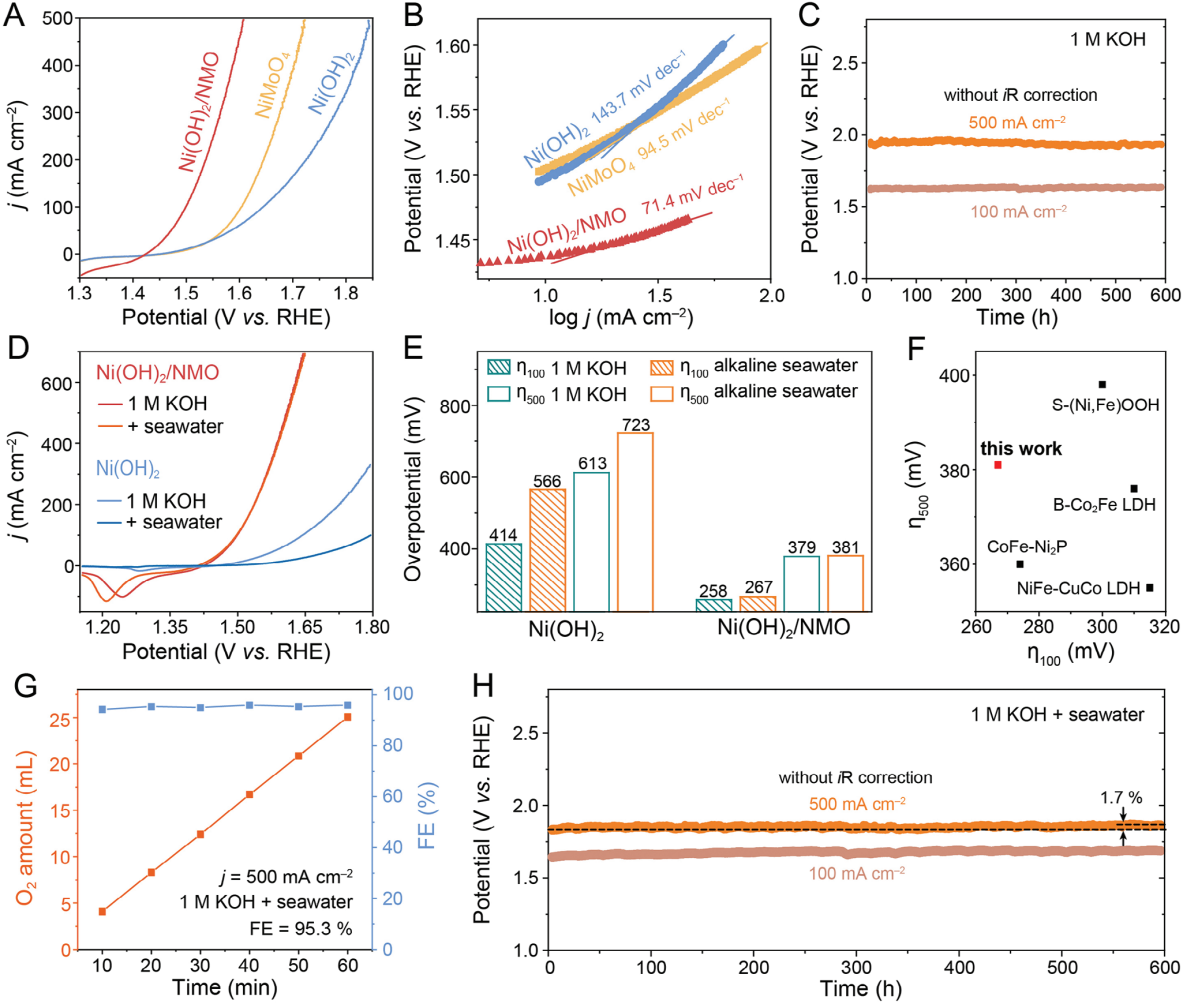
A) LSV curves (with *i*R correction) and B) the corresponding Tafel slopes of NiMoO_4_, Ni(OH)_2_, and Ni(OH)_2_/NMO. C) Chronopotentiometric curves of Ni(OH)_2_/NMO in 1 m KOH. D) LSV curves (with *i*R correction) and E) the corresponding overpotentials of Ni(OH)_2_/NMO and Ni(OH)_2_ in alkaline seawater oxidation. F) Comparison of OER overpotentials (at 100 and 500 mA cm^−2^) of Ni(OH)_2_/NMO with other electrocatalysts. G) FE (blue squares) of Ni(OH)_2_/NMO measured at 500 mA cm^−2^ in seawater + 1 m KOH. Orange squares show the O_2_ amount generated during the FE test. FE = 95.3% H) Chronopotentiometric curves of Ni(OH)_2_/NMO in 1 M KOH + seawater.

The OER activity of the as‐prepared samples was evaluated in an O_2_‐saturated 1 m KOH using a standard three‐electrode setup. **Figure** **2**A compares the linear sweep voltammograms (LSVs) recorded at a scan rate of 2 mV s^−1^. Notably, Ni(OH)_2_/NMO achieves a current density of 10 mA cm^−2^ at a significantly lower overpotential (η_10_) of 207 mV compared to Ni(OH)_2_ (267 mV) and NiMoO_4_ (274 mV, Figure S6, Supporting Information). Moreover, Ni(OH)_2_/NMO only requires 258 and 379 mV to reach current densities of 100 and 500 mA cm^−2^, respectively. Tafel analysis conducted on the polarization curves reveals a superior Tafel slope of 71.4 mV dec^−1^ for Ni(OH)_2_/NMO compared to the two counterparts (Figure 2B), indicating its faster reaction kinetics. Turnover frequency (TOF) is an important parameter for evaluating the intrinsic activity of electrocatalysts. The high TOF value of 0.016 s^−1^, measured at 1.540 V versus RHE, ≈2.72 times greater than Ni(OH)_2_ (0.006 s^−1^, Figure S7, Supporting Information), further highlights the enhanced intrinsic activity due to the heterojunctions in Ni(OH)_2_/NMO. The OER performance of Ni(OH)_2_/NMO is highly influenced by sample preparation conditions, as detailed in Figure S8 and Table S2 (Supporting Information). The sample synthesized via 10‐min Ni(OH)_2_ deposition exhibits the lowest resistance (3.0 Ω) at the reaction interface (R_ct_, Figure S9 and Table S3, Supporting Information), whereas both shorter and longer depositions (5 and 15 min) increase the R_ct_ (5.3 and 5.5 Ω, respectively). Long‐term chronopotentiometry without *i*R correction demonstrates the exceptional stability of Ni(OH)_2_/NMO during OER in 1 m KOH, maintaining current densities of 100 and 500 mA cm^−2^ for over 600 h (Figure 2C).

To assess its potential for seawater electrolysis application, the OER activity of Ni(OH)_2_/NMO was further evaluated in alkaline seawater electrolytes (natural seawater + 1 m KOH). As shown in Figure 2D, Ni(OH)_2_/NMO exhibits superior OER performance with minimal activity decline, maintaining almost the same activity as in freshwater. Compared to Ni(OH)_2_, its overpotentials at 100 and 500 mA cm^−2^ slightly increase by only 9 and 2 mV, respectively (Figure 2E), suggesting excellent OER selectivity of Ni(OH)_2_/NMO. This OER performance of Ni(OH)_2_/NMO surpasses or matches other state‐of‐the‐art catalysts designed for seawater oxidation (Figure 2F; Table S4, Supporting Information). Additionally, Ni(OH)_2_/NMO achieves a high average Faradaic efficiency (FE) of 95.3% at a high current density of 500 mA cm^−2^ in alkaline seawater (Figure 2G). Iodide titration confirms the high OER selectivity of Ni(OH)_2_/NMO against the hypochlorite reaction, as evidenced by the absence of a characteristic absorption peak for hypochlorite ions in the post‐FE test electrolyte (Figure S10, Supporting Information).^[^ ^39^ ^]^ Chronopotentiometry further reveals the superior long‐term electrocatalytic stability of Ni(OH)_2_/NMO during alkaline seawater OER. Remarkably, Ni(OH)_2_/NMO maintains excellent stability at both 100 and 500 mA cm^−2^ over 600 h (Figure 2H). The potential required to reach 500 mA cm^−2^ is lower in seawater compared to freshwater, attributed to the reduced Ohmic potential drop in ion‐rich seawater environments. While a slight increase (1.7%) is observed in the potential required to sustain 500 mA cm^−2^ for seawater OER, this is significantly lower than the 6.1% increase for Ni(OH)_2_ within just 2.5 h (Figure S11, Supporting Information). This further demonstrates the superior long‐term stability of Ni(OH)_2_/NMO in seawater oxidation. The post‐electrolysis SEM image of Ni(OH)_2_/NMO reveals that its hierarchical morphology is well retained after a 600‐h stability test (Figure S12, Supporting Information), supporting its high corrosion resistance in seawater. However, EDS mapping images detect the deposition of additional Ca and Mg elements, likely due to the formation of insoluble Ca(OH)_2_ and Mg(OH)_2_ on the catalyst surface during OER. These deposits may obscure active sites, potentially contributing to the increased potential required for long‐term reaction. To demonstrate the practical application for seawater splitting, a 1.5 V commercial battery was used to power a NiMo||Ni(OH)_2_/NMO electrolysis system (Figure S13, Supporting Information). The NiMo alloy was employed as the cathode because of its Pt‐like hydrogen evolution reaction performance (Figure S14, Supporting Information). The generation of bubbles on both electrodes underscores the potential of Ni(OH)_2_/NMO for practical seawater electrolysis.

### 2.3 MOR and Methanol‐Assisted Seawater Electrolysis

**Figure 3:**
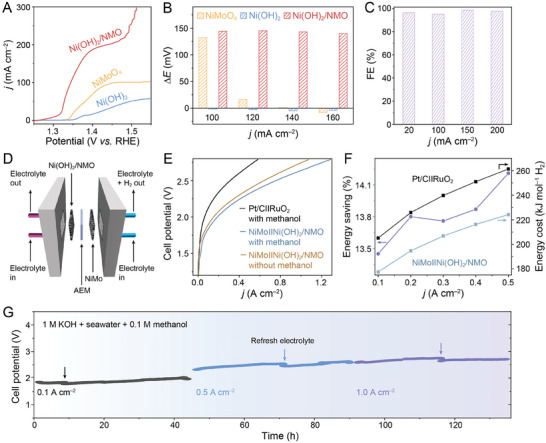
A) LSV curves of NiMoO_4_, Ni(OH)_2_, and Ni(OH)_2_/NMO in 1 m KOH + 0.1 m methanol. B) Potential differences between OER and MOR at various current densities. C) MOR FE at various current densities D) A schematic diagram of the MEA, where Ni(OH)_2_/NMO and NiMo are used as the anode and cathode, respectively. E) MEA polarization curve measured in various electrolytes. F) Energy costs of NiMo||Ni(OH)_2_/NMO and Pt/C||RuO_2_ at 0.1–0.5 A cm^−2^ and the corresponding energy savings. G) Chronopotentiograms of NiMo||Ni(OH)_2_/NMO measured at 0.1, 0.5, and 1.0 A cm^−2^ in alkaline seawater.

The methanol oxidation reaction (MOR) performances of Ni(OH)_2_, NiMoO_4_, and Ni(OH)_2_/NMO were investigated in an aqueous electrolyte containing 1 m KOH and 0.1 m methanol. As shown in **Figure** **3**A, Ni(OH)_2_/NMO exhibits a significantly lower MOR potential (1.305 V) to reach a current density of 10 mA cm^−2^ compared to Ni(OH)_2_ (1.372 V) and NiMoO_4_ (1.345 V). Furthermore, Ni(OH)_2_/NMO demonstrates the greatest potential difference between MOR and OER at all current densities tested (100, 120, 140, and 160 mA cm^−2^) compared with Ni(OH)_2_ and NiMoO_4_, (Figure 3B; Figure S15–S17, Supporting Information), which signifies superior MOR selectivity of Ni(OH)_2_/NMO. Chronopotentiometry at various current densities for 0.5 h was employed to determine the FE of the MOR process by Ni(OH)_2_/NMO. The stable *U*‐t curves indicate steady MOR between 20 and 200 mA cm^−2^ (Figure S18, Supporting Information). ^1^H nuclear magnetic resonance (NMR) spectroscopy further identifies and quantifies formate products (Figure S19, Supporting Information). Ni(OH)_2_/NMO maintains a high FE exceeding 95% at these current densities in alkaline methanol electrolytes.

To assess the potential for large‐scale applications, a 1 cm^2^ Ni(OH)_2_/NMO electrode was paired with a 1 cm^2^ NiMo cathode in a membrane electrode assembly (MEA), as depicted in Figure 3D (with the setup shown in Figure S20, Supporting Information). In a hybrid seawater electrolyte (1 m KOH + 0.1 m methanol + seawater) at 23 °C, the NiMo||Ni(OH)_2_/NMO system achieves current densities of 0.1 and 0.5 A cm^−2^ at considerably lower cell voltages (1.840 and 2.324 V, respectively) compared to the alkaline‐seawater electrolyte (1.904 and 2.392 V, respectively; Figure 3E; Figure S21, Supporting Information). The calculated energy consumption for H_2_ production using the NiMo||Ni(OH)_2_/NMO system are 177.5 and 224.2 kJ mol^−1^ H_2_ at current densities of 0.1 and 0.5 A cm^−2^, respectively (Figure 3F; Table S5, Supporting Information). These values are substantially lower than those for the commercial Pt/C||RuO_2_ system (205.1 and 261.4 kJ mol^−1^ H_2_, respectively), representing energy savings of over 13.5%. This highlights the potential of the NiMo||Ni(OH)_2_/NMO system for cost‐effective and practical H_2_ production from seawater, offering a more energy‐efficient alternative to existing technologies. Importantly, the NiMo||Ni(OH)_2_/NMO flow cell demonstrates stable and continuous operation for over 130 h at current densities of 0.1, 0.5, and 1.0 A cm^−2^ with minimal performance degradation (Figure 3G).

### 2.4 Mechanisms and Anti‐Corrosion Properties of Ni(OH)_2_/NMO

To elucidate the mechanism responsible for the enhanced OER performance, electrochemical impedance spectroscopy (EIS) was conducted. The corresponding Nyquist plots were analyzed to investigate the charge transfer dynamics at the electrode/electrolyte interface (Figure S22, Supporting Information). The resistance at this interface (*R*_ct_) is significantly reduced for Ni(OH)_2_/NMO (2.9 Ω) compared to Ni(OH)_2_ (17.5 Ω) and NiMoO_4_ (20.1 Ω). This indicates a lower charge transfer barrier in the composite material. We further assessed the electrochemically active surface area (ECSA) of the as‐prepared catalysts by measuring the electrochemical double‐layer capacitances (*C*_dl_) in a non‐Faradaic potential region at various scan rates (Figures S23 and S24, Supporting Information). Ni(OH)_2_/NMO exhibits the largest ECSA, exceeding that of Ni(OH)_2_ and NiMoO_4_ by factors of 1.46 and 1.54, respectively (Figure S25, Supporting Information). This suggests that the hierarchical structure provides a significantly larger surface area with more accessible active sites for the OER. Interestingly, even after normalizing the polarization curves by ECSA, the OER activity of Ni(OH)_2_/NMO remains superior to Ni(OH)_2_ and NiMoO_4_ (Figure S26, Supporting Information), which implies that its excellent performance is not solely due to the increased ECSA from the hierarchical structure but also a higher intrinsic activity. Furthermore, Arrhenius plots were obtained from LSVs conducted at various temperatures (Figures S27 and S28, Supporting Information) and used to determine the activation energy (*E*_a_) from their slopes. Ni(OH)_2_/NMO displays the lowest *E*_a_ of 23.22 kJ mol^−1^, followed by NiMoO_4_ (32.37 kJ mol^−1^) and Ni(OH)_2_ (50.39 kJ mol^−1^). This lower activation energy suggests that the OER process on Ni(OH)_2_/NMO proceeds more readily compared to the other two materials.

**Figure 4:**
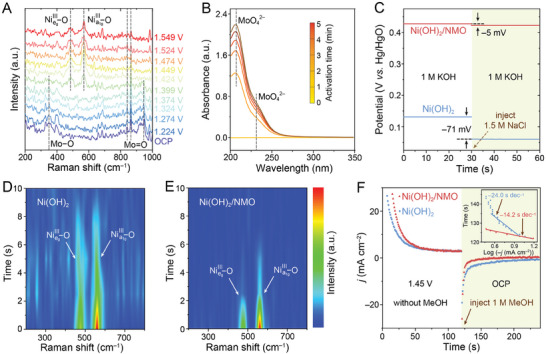
A) In situ Raman spectra of Ni(OH)_2_/NMO. B) Quasi‐in situ UV–vis spectra for MoO_4_^2−^ detection. C) OCP measurements of Ni(OH)_2_ and Ni(OH)_2_/NMO upon NaCl injection. Quasi‐in situ Raman spectra of oxidized D) Ni(OH)_2_ and E) Ni(OH)_2_/NMO during the reaction with methanol. F) Chronoamperometric responses of Ni(OH)_2_ and Ni(OH)_2_/NMO upon methanol injection.

In situ Raman spectroscopy was employed to track the real‐time evolution of surface species during OER. Figure S29 (Supporting Information) shows the Raman spectra of Ni(OH)_2_, captured from the open‐circuit potential (OCP) to an applied potential of 1.499 V (vs RHE). At 1.399 V, the characteristic peaks for NiOOH species, associated with the Ni^3+^(*e*_g_)–O and Ni^3+^(*a*_1_ _g_)–O vibration modes, are observed at 476 (peak i) and 558 cm^−1^ (peak ii), respectively,^[^ ^40^ ^]^ signifying a transformation from the starting materials during the OER process. The intensity ratio of these peaks (I_558_/I_476_) increases from 1.44 at 1.399 V to 1.64 at 1.499 V (Figure S30, Supporting Information). The pronounced increase in the peak ii intensity, relative to peak i, suggests a phase transition from the initial γ‐NiOOH to the more active β‐NiOOH phase at higher anodic potentials.^[^ ^41^, ^42^ ^]^ Similar trends are observed for Ni(OH)_2_/NMO (**Figure** **4**A), but the transition to the β‐NiOOH phase occurs at a lower potential, as reflected by a higher I_558_/I_476_ ratio of 1.97 at 1.424 V. Besides, Mo═O bending (347 cm^−1^) and symmetric Mo═O stretching (941 cm^−1^) disappear when the potential increases to 1.399 V, which suggests the partial collapse of NiMoO_4_ structure. On the other hand, NiMoO_4_ exhibits sluggish phase transition during the OER activation process, which can be related to its poorer catalytic performance (Figure S31, Supporting Information). XRD confirms the formation of NiOOH in Ni(OH)_2_/NMO after OER activation as evidenced by the lattice spacings of 2.39 and 2.08 Å, corresponding to the (011) and (210) plane of NiOOH, respectively, (Figure S32, Supporting Information). Further analysis using XPS reveals the shifts in Mo 3*d* peaks toward lower binding energies by 0.14 eV, indicating an increase in electron density around Mo and the charge transfer during the OER activation process (Figure S33, Supporting Information).^[^ ^43^ ^]^ Notably, the peak intensity of these Mo peaks decreases with higher potentials, suggesting a loss of Mo during OER activation. Quasi‐in situ UV–vis spectroscopy corroborated the release of MoO_4_^2−^ anion (Figure S34, Supporting Information). An absorption peak for MoO_4_^2−^ appears at ≈209 and 231 nm after applying 2.5 V to Ni(OH)_2_/NMO, and its intensity increases with prolonged oxidation processes (Figure 4B). This observation confirms the release of MoO_4_^2−^ from NiMoO_4_ during OER. It is worth noting that these released anions can change the local coordination environment of NiOOH, driven by the positive potential of the anode, thereby influencing the adsorption behaviors of other molecules, such as Cl^−^ and methanol, and the reaction kinetics of CER and MOR.^[^ ^44^ ^]^

The OCP measurement can provide insights into the impact of Cl^−^ adsorption on the Helmholtz layer of catalysts. A larger OCP shift upon introducing Cl^−^ indicates a stronger influence of Cl^−^ on the catalyst surface.^[^ ^45^, ^46^ ^]^ Upon the addition of 1.5 m NaCl, the OCP of Ni(OH)_2_/NMO slightly decreases by 5 mV, while Ni(OH)_2_ exhibits a significant drop of 71 mV (Figure 4C), suggesting a weaker impact of Cl^−^ on Ni(OH)_2_/NMO. Notably, Raman spectroscopy performed after a 600‐h stability test in alkaline seawater confirms the persistence of NiOOH and symmetric Mo═O stretching mode (Figure S35, Supporting Information).^[^ ^47^, ^48^ ^]^ This highlights the durability and corrosion resistance of the Ni(OH)_2_/NMO catalyst under harsh conditions.

In situ Raman spectroscopy was conducted to validate the mechanism of enhanced MOR performance of Ni(OH)_2_/NMO. For Ni(OH)_2_, the characteristic peak for Ni^3+^–O appears at a higher potential (1.424 V, Figure S36, Supporting Information) in a KOH–methanol electrolyte than in the blank KOH (Figure S29, Supporting Information), indicating that the conversion of Ni^2+^ to Ni^3+^ is retarded until 1.424 V. This is due to the MOR process that consumes produced Ni^3+^ species. The accumulation of Ni^3+^ above 1.424 V can be attributed to the accelerated Ni^3+^ generation rate over the methanol dehydrogenation rate. The further delayed appearance of the Ni^3+^–O peak (1.449 V, Figure S37, Supporting Information) indicates the faster methanol dehydrogenation rate on Ni(OH)_2_/NMO. We further investigated the non‐electrochemical step using quasi‐*operando* Raman spectroscopy. The electrodes were first oxidized at a fixed potential of 1.50 V, followed by methanol addition and Raman measurement at different reaction time intervals. The Ni^3+^–O vibration mode on Ni(OH)_2_ weakens and disappears after 10 s of reaction with methanol (Figure 4D). Notably, the Ni^3+^–O peak in Ni(OH)_2_/NMO vanishes much faster, completely disappearing within 5 s (Figure 4E). This implies a significantly faster hydrogen transfer rate during the non‐electrochemical step for Ni(OH)_2_/NMO. Periodic electrochemical measurements support this observed change in the hydrogen transfer rate (Figure 4F). After the initial activation by applying 1.45 V for 120 s, the transient current change was monitored upon methanol injection. The current response of Ni(OH)_2_/NMO drops to 0 mA cm^−2^ faster than Ni(OH)_2_. The calculated slope for Ni(OH)_2_/NMO (−14.2 s dec^−1^; inset in Figure 4F) is much lower than that of Ni(OH)_2_ (−24.0 s dec^−1^), confirming faster catalytic kinetics of the PCET process in Ni(OH)_2_/NMO. The change in methanol adsorption within the Helmholtz layer was also assessed by OCP measurements. A significantly larger OCP drop (0.244 V) is observed for Ni(OH)_2_/NMO compared to Ni(OH)_2_ upon adding methanol (Figure S38, Supporting Information). This indicates a stronger interaction between methanol and Ni(OH)_2_/NMO, potentially leading to enhanced MOR activity.

*Operando* electrochemical impedance spectroscopy (EIS) provided further insights into the interfacial dynamics and electron transfer mechanisms. The low‐frequency domain (0.01–10 Hz) in the Bode plots (Figures S39 and S40, Supporting Information) corresponds to the MOR and OER interfaces, while the high‐frequency region (10–10^5^ Hz) reflects surface oxidation processes.^[^ ^49^, ^50^ ^]^ Compared to Ni(OH)_2_, the phase angle of transition peaks attributed to MOR (0.1–1 Hz) on Ni(OH)_2_/NMO is smaller at the same voltage. This suggests a faster conversion rate of oxide species at the interface. Nyquist plots offer additional information about the interfacial resistances at various potentials (Figure S41 and Table S6, Supporting Information). Ni(OH)_2_/NMO consistently exhibits lower resistance at the electrode/electrolyte interface compared to Ni(OH)_2_, indicating enhanced charge transfer efficiency (Figure S42, Supporting Information). The peak observed at 10–100 Hz in Ni(OH)_2_/NMO can be attributed to the oxidation of NiMoO_4_, consistent with the UV–vis spectra (Figure S34, Supporting Information).

Density functional theory (DFT) calculations were conducted to gain insights into how MoO_4_^2−^ enhances the anti‐corrosion property, water oxidation activity, and methanol oxidation selectivity of Ni(OH)_2_/NMO. The activated states of Ni(OH)_2_ and Ni(OH)_2_/NMO were modeled using the crystal structures of NiOOH and MoO_4_^2−^‐adsorbed NiOOH (Mo‐NiOOH), respectively. The (010) surface of NiOOH was chosen as the model (Figure S43, Supporting Information) based on its reported low reaction barrier.^[^ ^51^, ^52^, ^53^ ^]^ Among four adsorption sites on the NiOOH surface considered (Figure S44, Supporting Information), the most favorable configuration involves MoO_4_^2−^ coordinated with four O atoms, with an adsorption free energy of −65.3 eV (Figure S45, Supporting Information). This configuration was selected for further calculations. The corresponding charging density difference image reveals that the MoO_4_^2−^ exhibits strong chemisorption on NiOOH by forming a Ni─O─Mo bond (Figure S46, Supporting Information).

**Figure 5:**
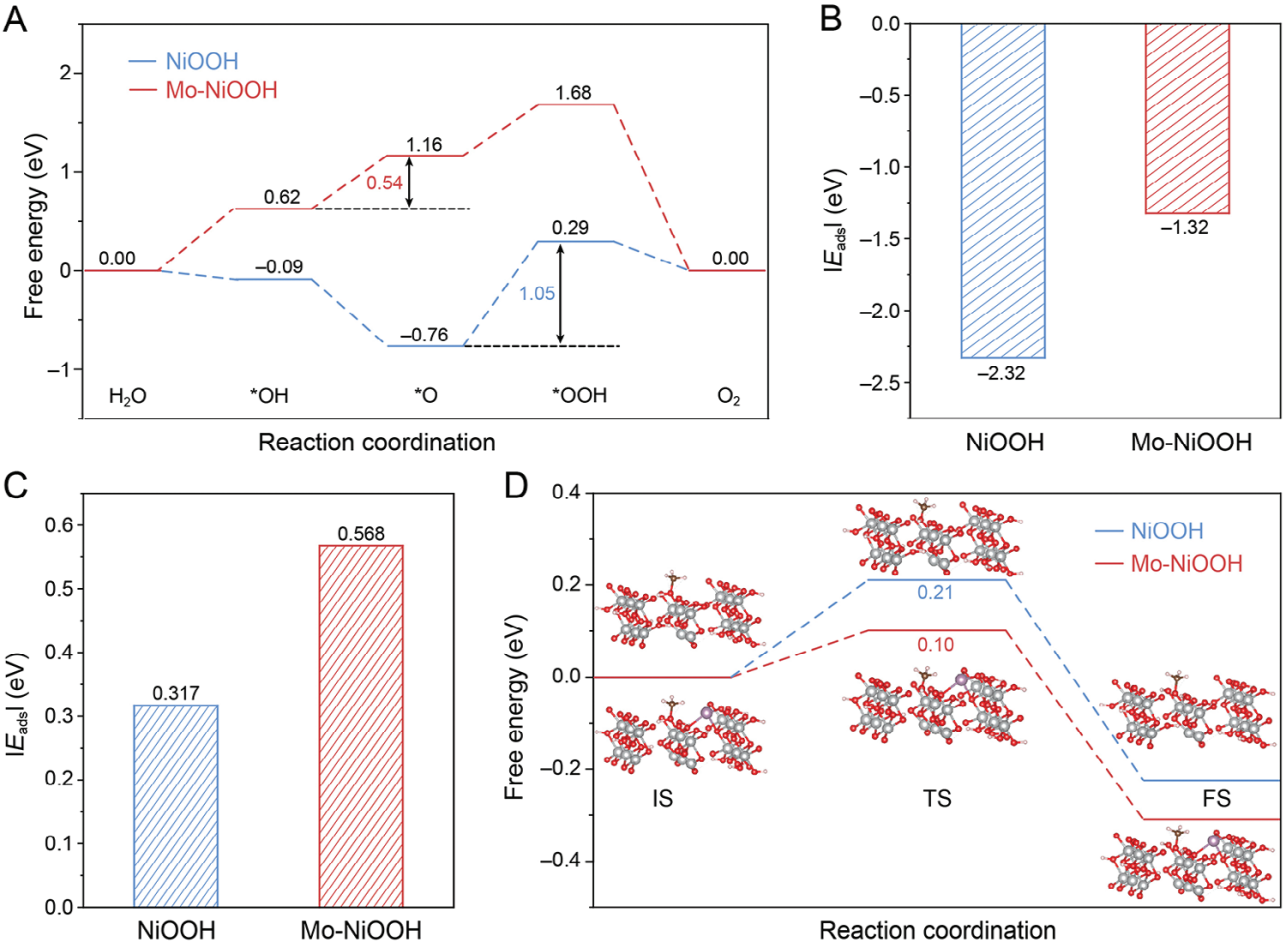
A) Gibbs free energy diagrams for OER. Adsorption‐free energies of B) Cl^−^ and C) methanol on NiOOH and Mo‐NiOOH. D) Free energy changes in the hydrogen transfer process on NiOOH and Mo‐NiOOH.

DFT calculations were employed to calculate the free energy changes at each step of the OER (Figures S47 and S48, Supporting Information). The OER free energy diagram (**Figure** **5**A) reveals that the second step, involving deprotonation and electron transfer from *OH to form *O, is the rate‐determining step (RDS) for NiOOH. Interestingly, in Mo‐NiOOH, the RDS shifts to the third step (*OOH formation). Consequently, the overall OER free energy is significantly reduced from 1.05 eV for NiOOH to 0.54 eV for Mo‐NiOOH, highlighting the beneficial effects of MoO_4_^2−^ adsorption on OER catalysis. Additionally, Mo‐NiOOH exhibits an increased Cl^−^ adsorption energy of −1.32 eV compared to NiOOH (−2.32 eV, Figure 5B; Figures S49 and S50, Supporting Information), which indicates that the leached MoO_4_^2−^ effectively reduces the Cl^−^ adsorption on the NiOOH surface, thereby suppressing CER and enhancing the corrosion resistance of the electrode.

During methanol adsorption on the Mo‐NiOOH surface, an energy of 0.568 eV is released (Figure 5C), which exceeds the energy released on the NiOOH surface (0.317 eV). This indicates a more favorable adsorption of methanol on Mo‐NiOOH. To gain a theoretical understanding of the influence of MoO_4_^2−^ adsorption on the PCET process, DFT calculations were further engaged to analyze the hydrogen transfer energy barrier. As the core mechanism of methanol electrooxidation involves proton transfer from the substrate molecule to NiOOH, the energy barriers of proton transfer from methanol to NiOOH or Mo‐NiOOH surface were calculated using optimized models of the initial state (IS), transition state (TS), and final state (FS, Figure 5d; Figure S51 and S52, Supporting Information). The energy barrier for the PCET process on NiOOH is reduced from 0.21 to 0.10 eV after MoO_4_^2−^ decoration, indicating a faster rate of the PCET process and an accelerated cycle between Ni^3+^ reduction and methanol dehydrogenation.

## 3 Conclusion

In summary, this study demonstrates that the incorporation of MoO_4_^2−^ effectively improves the methanol oxidation activity of NiOOH during seawater electrolysis, simultaneously protecting NiOOH from chlorine‐induced corrosion. The 3D hierarchical structure of Ni(OH)_2_/NMO pre‐catalyst expands the ECSA, providing an abundance of active sites. Additionally, the interfacial interaction between Ni(OH)_2_ and NiMoO_4_ facilitates the reconstruction process to form MOR‐ and OER‐activated NiOOH. Furthermore, in situ leached MoO_4_^2−^ modifies the surface microenvironment of NiOOH. These anions decrease the adsorption of Cl^−^, thereby mitigating electrode corrosion. The adsorbed MoO_4_^2−^ also facilitates the adsorption of methanol and concurrently reduces the energy barrier for the non‐electrochemical process, promoting the PCET during MOR. Consequently, Ni(OH)_2_/NMO catalyst exhibits significantly enhanced OER activity and durability in alkaline seawater, achieving 100 mA cm^−2^ at 267 mV and demonstrating remarkable long‐term stability, with only a 1.7% activity loss for 600 h at 500 mA cm^−2^. When combined with a NiMo cathode, the NiMo||Ni(OH)_2_/NMO cell requires only 1.305 V to reach 10 mA cm^−2^ in seawater, assisted by methanol oxidation. Compared with Pt/C||RuO_2_, the NiMo||Ni(OH)_2_/NMO cell offers a 13.5% energy saving for hydrogen production at 0.5 A cm^−2^ in hybrid seawater. This cell demonstrates stable operation for over 130 h at 0.1, 0.5, and 1.0 A cm^−2^. This study introduces a novel design concept for modulating the catalytic surface microenvironment, paving the way for Ni‐based catalysts with high selectivity toward small molecule oxidation reactions and strong anti‐corrosion properties in hybrid seawater electrolysis.

## 4 Experimental Section

Details of chemicals, materials synthesis methods, characterizations, electrochemical and flow cell measurements, energy consumption calculations, and theoretical calculations are presented in the Supporting Information.

## Conflict of Interest

The authors declare no conflict of interest.

## Author Contributions

Z.L. and Y.Z. contributed equally to this work. Z.L. and L.Y.S.L. conceived the conceptual idea and designed the experiments. Z.L. and W.Z. synthesized materials. Y.Z. conducted theoretical calculations and formal analysis under the supervision of L.D. Z.L. conducted physical characterization, electrochemical tests, formal analysis, and wrote the final manuscript. L.Y.S.L. acquired funding and resources, supervised the project, and revised the manuscript.

## Supporting information



Supporting Information

## Data Availability

The data that support the findings of this study are available from the corresponding author upon reasonable request.

## References

[1] S. Sanati , A. Morsali , H. Garcia , Energy Environ. Sci. 2022, 15, 3119.

[2] Q. Zhu , T. Zhang , X. Zhu , J. Zhang , M. Shan , Z. Hu , G. Xu , M. Zhu , Energy Mater 2024, 4, 400016.

[3] J. Peng , W. Dong , Z. Wang , Y. Meng , W. Liu , P. Song , Z. Liu , Mater. Today Adv. 2020, 8, 100081.

[4] J. F. Chang , G. Z. Wang , Z. Z. Yang , B. Y. Li , Q. Wang , R. Kuliiev , N. Orlovskaya , M. Gu , Y. G. Du , G. F. Wang , Y. Yang , Adv. Mater. 2021, 33, 2101425. 10.1002/adma.202101425 34235791

[5] Y. Zhao , Z. Yu , A. Ge , L. Liu , J. L. Faria , G. Xu , M. Zhu , Green Energy Environ. 2024, 10.1016/j.gee.2024.02.001.

[6] F. S. Hegner , F. A. Garces‐Pineda , J. Gonzalez‐Cobos , B. Rodriguez‐Garcia , M. Torrens , E. Palomares , N. Lopez , J. R. Galan‐Mascaros , ACS Catal. 2021, 11, 13140.

[7] X. Luo , P. X. Ji , P. Y. Wang , X. Tan , L. Chen , S. C. Mu , Adv. Sci. 2022, 9, 2104846. 10.1002/advs.202104846 PMC8895145 35243823

[8] P. Shen , B. W. Zhou , Z. Chen , W. P. Xiao , Y. L. Fu , J. Wan , Z. X. Wu , L. Wang , Appl. Catal. B‐Environ. Energy 2023, 325, 122305.

[9] L. S. Zhang , L. P. Wang , H. P. Lin , Y. X. Liu , J. Y. Ye , Y. Z. Wen , A. Chen , L. Wang , F. L. Ni , Z. Y. Zhou , S. G. Sun , Y. Y. Li , B. Zhang , H. S. Peng , Angew. Chem., Int. Ed. 2019, 58, 16820. 10.1002/anie.201909832 31535447

[10] C. F. Liu , X. R. Shi , K. H. Yue , P. J. Wang , K. Zhan , X. Y. Wang , B. Y. Xia , Y. Yan , Adv. Mater. 2023, 35, 2211177. 10.1002/adma.202211177 36606317

[11] J. X. Zhu , L. X. Xia , R. H. Yu , R. H. Lu , J. T. Li , R. H. He , Y. C. Wu , W. Zhang , X. F. Hong , W. Chen , Y. Zhao , L. Zhou , L. Q. Mai , Z. Y. Wang , J. Am. Chem. Soc. 2022, 144, 15529. 35943197 10.1021/jacs.2c03982

[12] Z. P. Yu , L. F. Liu , Adv. Mater. 2024, 36, 2308647. 10.1002/adma.202308647 38143285

[13] Y. Yang , X. X. Wu , M. Ahmad , F. Z. Si , S. J. Chen , C. H. Liu , Y. Zhang , L. Wang , J. J. Zhang , J. L. Luo , X. Z. Fu , Angew. Chem., Int. Ed. 2023, 62, e202302950. 10.1002/anie.202302950 36946249

[14] Z. P. Yu , G. D'Olimpio , H. L. Huang , C. N. Kuo , C. S. Lue , G. Nicotra , F. Lin , D. W. Boukhvalov , A. Politano , L. F. Liu , Adv. Funct. Mater. 2024, 34, 2403099.

[15] K. Y. Xu , L. C. Liang , T. Li , M. J. Bao , Z. P. Yu , J. W. Wang , S. M. Thalluri , F. Lin , Q. B. Liu , Z. M. Cui , S. Q. Song , L. F. Liu , Adv. Mater. 2024, 36, 2403792. 10.1002/adma.202403792 38742953

[16] J. S. Li , L. M. Li , J. Wang , A. Cabot , Y. F. Zhu , ACS Energy Lett. 2024, 9, 853.

[17] J. S. Li , X. Tian , X. Wang , T. Zhang , M. C. Spadaro , J. Arbiol , L. M. Li , Y. Zuo , A. Cabot , Inorg. Chem. 2022, 61, 13433. 35983854 10.1021/acs.inorgchem.2c01695

[18] B. Zhao , J. W. Liu , X. W. Wang , C. Y. Xu , P. F. Sui , R. F. Feng , L. Wang , J. J. Zhang , J. L. Luo , X. Z. Fu , Nano Energy 2021, 80, 105530.

[19] W. Chen , J. Q. Shi , C. Xie , W. Zhou , L. T. Xu , Y. Y. Li , Y. D. Wu , B. B. Wu , Y. C. Huang , B. Zhou , M. Yang , J. L. Liu , C. L. Dong , T. H. Wang , Y. Q. Zou , S. Y. Wang , Natl. Sci. Rev. 2023, 10, nwad099. 37287808 10.1093/nsr/nwad099 PMC10243987

[20] S. L. Li , R. G. Ma , J. C. Hu , Z. C. Li , L. J. Liu , X. L. Wang , Y. Lu , G. E. Sterbinsky , S. H. Liu , L. Zheng , J. Liu , D. M. Liu , J. C. Wang , Nat. Commun. 2022, 13, 2916. 35614111 10.1038/s41467-022-30670-4 PMC9133001

[21] B. You , N. Jiang , X. Liu , Y. J. Sun , Angew. Chem., Int. Ed. 2016, 55, 9913. 10.1002/anie.201603798 27417546

[22] Y. Huang , X. D. Chong , C. B. Liu , Y. Liang , B. Zhang , Angew. Chem., Int. Ed. 2018, 57, 13163. 10.1002/anie.201807717 30118157

[23] Y. M. Shi , W. Du , W. Zhou , C. H. Wang , S. S. Lu , S. Y. Lu , B. Zhang , Angew. Chem., Int. Ed. 2020, 59, 22470. 10.1002/anie.202011097 32897620

[24] W. M. Tong , M. Forster , F. Dionigi , S. Dresp , R. S. Erami , P. Strasser , A. J. Cowan , P. Farràs , Nat. Energy 2020, 5, 367.

[25] P. F. Guo , D. Liu , R. B. Wu , Small Struct. 2023, 4, 2300192.

[26] L. Yu , L. B. Wu , B. McElhenny , S. W. Song , D. Luo , F. H. Zhang , Y. Yu , S. Chen , Z. F. Ren , Energy Environ. Sci. 2020, 13, 3439.

[27] B. S. Zhang , S. Liu , S. J. Zhang , Y. Cao , H. L. Wang , C. Y. Han , J. Sun , SmallSmall 2022, 18, 2203852.

[28] Z. Li , M. J. Liu , J. Yan , L. Y. S. Lee , Chem. Eng. J. 2023, 473, 145293.

[29] L. Yu , Q. Zhu , S. W. Song , B. McElhenny , D. Z. Wang , C. Z. Wu , Z. J. Qin , J. M. Bao , Y. Yu , S. Chen , Z. F. Ren , Nat. Commun. 2019, 10, 5106. 31704926 10.1038/s41467-019-13092-7 PMC6841982

[30] R. N. Dürr , P. Maltoni , H. N. Tian , B. Jousselme , L. Hammarström , T. Edvinsson , ACS Nano 2021, 15, 13504. 34383485 10.1021/acsnano.1c04126 PMC8388116

[31] S. Q. Niu , Y. C. Sun , G. J. Sun , D. Rakov , Y. Z. Li , Y. Ma , J. Y. Chu , P. Xu , ACS Appl. Energy Mater. 2019, 2, 3927.

[32] G. Solomon , A. Landström , R. Mazzaro , M. Jugovac , P. Moras , E. Cattaruzza , V. Morandi , I. Concina , A. Vomiero , Adv. Energy Mater. 2021, 11, 2101324.

[33] J. R. Ran , W. W. Guo , H. L. Wang , B. C. Zhu , J. G. Yu , S. Z. Qiao , Adv. Mater. 2018, 30, 1800128. 10.1002/adma.201800128 29707838

[34] Y. N. Zhou , W. L. Yu , Y. N. Cao , J. Zhao , B. Dong , Y. Ma , F. L. Wang , R. Y. Fan , Y. L. Zhou , Y. M. Chai , Appl. Catal. B‐Environ. Energy 2021, 292, 120150.

[35] Z. Y. Zhao , Q. Shao , J. Y. Xue , B. L. Huang , Z. Niu , H. W. Gu , X. Q. Huang , J. P. Lang , Nano Res. 2022, 15, 310.

[36] K. Dastafkan , X. J. Shen , R. K. Hocking , Q. Meyer , C. Zhao , Nat. Commun. 2023, 14, 547. 36725848 10.1038/s41467-023-36100-3 PMC9892594

[37] L. N. Sha , K. Ye , J. L. Yin , K. Zhu , K. Cheng , J. Yan , G. L. Wang , D. X. Cao , Chem. Eng. J. 2020, 381, 122603.

[38] L. Liu , Y. C. He , Q. Li , C. S. Cao , M. H. Huang , D. D. Ma , X. T. Wu , Q. L. Zhu , Exploration 2024, 4, 20230043. 38939862 10.1002/EXP.20230043 PMC11189569

[39] H. J. Song , H. Yoon , B. Ju , D. Y. Lee , D. W. Kim , ACS Catal. 2020, 10, 702.

[40] M. X. Chen , Y. Y. Zhang , R. Wang , B. Zhang , B. Song , Y. C. Guan , S. W. Li , P. Xu , J. Energy Chem. 2023, 84, 173.

[41] B. J. Trzesniewski , O. Diaz‐Morales , D. A. Vermaas , A. Longo , W. Bras , M. T. M. Koper , W. A. Smith , J. Am. Chem. Soc. 2015, 137, 15112. 26544169 10.1021/jacs.5b06814

[42] M. J. Liu , K. A. Min , B. Han , L. Y. S. Lee , Adv. Energy Mater. 2021, 11, 2101281.

[43] J. Jiang , F. F. Sun , S. Zhou , W. Hu , H. Zhang , J. C. Dong , Z. Jiang , J. J. Zhao , J. F. Li , W. S. Yan , M. Wang , Nat. Commun. 2018, 9, 2885. 30038335 10.1038/s41467-018-05341-y PMC6056503

[44] T. F. Ma , W. W. Xu , B. R. Li , X. Chen , J. J. Zhao , S. S. Wan , K. Jiang , S. X. Zhang , Z. F. Wang , Z. Q. Tian , Z. Y. Lu , L. Chen , Angew. Chem., Int. Ed. 2021, 60, 22740. 10.1002/anie.202110355 34431193

[45] Z. H. Yang , S. Wang , C. Y. Wei , L. Chen , Z. M. Xue , T. C. Mu , Energy Environ. Sci. 2024, 17, 1603.

[46] Y. X. Lu , T. Y. Liu , C. L. Dong , Y. C. Huang , Y. F. Li , J. Chen , Y. Q. Zou , S. Y. Wang , Adv. Mater. 2021, 33, 2007056. 10.1002/adma.202007056 33470476

[47] B. Konkena , J. Masa , A. J. R. Botz , I. Sinev , W. Xia , J. Kossmann , R. Drautz , M. Muhler , W. Schuhmann , ACS Catal. 2017, 7, 229.

[48] J. Huang , Y. Li , Y. Zhang , G. Rao , C. Wu , Y. Hu , X. Wang , R. Lu , Y. Li , J. Xiong , Angew. Chem., Int. Ed. 2019, 58, 17458. 10.1002/anie.201910716 31550415

[49] S. Q. Li , S. B. Wang , Y. H. Wang , J. H. He , K. Li , Y. J. Xu , M. X. Wang , S. Y. Zhao , X. N. Li , X. Zhong , J. G. Wang , Adv. Funct. Mater. 2023, 33, 2214488.

[50] Y. X. Lu , T. Y. Liu , C. L. Dong , C. M. Yang , L. Zhou , Y. C. Huang , Y. F. Li , B. Zhou , Y. Q. Zou , S. Y. Wang , Adv. Mater. 2022, 34, 2107185. 10.1002/adma.202107185 34655453

[51] S. H. Lee , Y. C. Chu , L. C. Bai , H. M. Chen , X. L. Hu , Chem Catal 2023, 3, 100475.

[52] Z. Y. Wang , W. A. Goddard , H. Xiao , Nat. Commun. 2023, 14, 4228. 37454140 10.1038/s41467-023-40011-8 PMC10349880

[53] F. Dionigi , Z. H. Zeng , I. Sinev , T. Merzdorf , S. Deshpande , M. B. Lopez , S. Kunze , I. Zegkinoglou , H. Sarodnik , D. X. Fan , A. Bergmann , J. Drnec , J. F. de Araujo , M. Gliech , D. Teschner , J. Zhu , W. X. Li , J. Greeley , B. Roldan Cuenya , P. Strasser , Nat. Commun. 2020, 11, 2522. 32433529 10.1038/s41467-020-16237-1 PMC7239861

