# Supplementary material for: Molybdate‐Modified NiOOH for Efficient Methanol‐Assisted Seawater Electrolysis
Source: Adv Sci (Weinh). 2025 Feb 19;12(14):2410911. doi: 10.1002/advs.202410911 (PMC11984929; doi:10.1002/advs.202410911)
Supplement: Supplementary file 1 — Supporting Information [file ADVS-12-2410911-s001.docx]

Supporting information

**Molybdate-Modified NiOOH for Efficient Methanol-Assisted Seawater Electrolysis**

Zhen Li,^†^ Youbin Zheng,^†^ Wenhan Zu, Liang Dong,* Lawrence Yoon Suk Lee*

Z. Li, W. Zu, Prof. L. Y. S. Lee

*Department of Applied Biology and Chemical Technology and Research Institute for Smart Energy, The Hong Kong Polytechnic University, Hung Hom, Kowloon, Hong Kong SAR, China*

*E-mail address*: lawrence.ys.lee@polyu.edu.hk (L. Y. S. Lee)

Y. Zheng, Prof. L. Dong

*Key Laboratory of Dielectric and Electrolyte Functional Material, School of Resources and Materials, Northeastern University at Qinhuangdao, Qinhuangdao, 066004, Hebei Province, China*

*E-mail address*: dongliang@neuq.edu.cn (L. Dong)

**Experimental Section**

*1. Chemicals*

Nickel nitrate hexahydrate (Ni(NO_3_)_2_·6H_2_O, 99.9 %), Nafion perfluorinated resin solution (5 wt.%), sodium molybdate dihydrate (NaMoO_4_∙2H_2_O, 99.0 %), potassium nitrate (KNO_3_, 99.0 %), and potassium hydroxide (KOH, 99.9 %) were purchased from Sigma–Aldrich. Sulfuric acid (H_2_SO_4_, 98.0 %), methanol (99.8 %), and acetone (99.8 %) were purchased from Duksan Chemicals. Ammonium molybdate tetrahydrate ((NH_4_)_6_Mo_7_O_24_∙4H_2_O, 83.0 %), sodium chloride (NaCl, 99.5 %), and potassium iodide (KI, 99.0 %) were obtained from Shenzhen Dieckmann Tech. Aqueous solutions were prepared using deionized (DI) water produced by MilliQ Water System. Natural seawater (pH = ~8) was collected from Hung Hom Bay near the Hong Kong Polytechnic University, Kowloon, Hong Kong SAR, China. All chemicals were used as received.

*2. Preparation of Materials*

*Synthesis of NiMoO_4_*: NiMoO_4_ nanorods were directly grown on Ni foam through a hydrothermal method. A Ni foam (1.0 × 3.5 cm^2^) was cleaned by sonication in acetone and washed with 3 M HCl for 10 min to remove surface oxides. A solution was then prepared by dissolving Ni(NO_3_)_2_∙6H_2_O (0.175 g) and Na_2_MoO_4_∙2H_2_O (0.145 g) in DI water (10 mL) under magnetic stirring for 30 min. This solution was transferred to a 15 mL Teflon-lined autoclave containing the Ni foam. The autoclave was sealed and heated at 150 °C for 4 h. After cooling to room temperature, the sample was washed with DI water several times and dried at 60 °C under vacuum for 12 hours.

*Synthesis of* *Ni(OH)_2_/NiMoO_4_ (Ni(OH)_2_/NMO)*: An electrodeposition method was engaged to prepare Ni(OH)_2_/NMO. As-prepared NiMoO_4_ on Ni foam (0.25 cm^2^) served as the working electrode in a three-electrode configuration. A standard calomel electrode and a carbon rod were employed as the reference and counter electrodes, respectively, in an aqueous electrolyte. Ni(OH)_2_ was electrodeposited onto the NiMoO_4_ nanorods by applying −1 V for various durations in an electrolyte (50 mL) containing Ni(NO_3_)_2_ (1.0 M) and KNO_3_ (0.2 M). The resulting Ni(OH)_2_/NMO composite was rinsed with DI water and dried at 60 °C under vacuum for 12 h. For comparison, a control Ni(OH)_2_ electrode was prepared using the same procedure but on pristine Ni foam (0.25 cm^2^) without NMO nanorods. Separately, Pt/C (or RuO_2_) catalyst ink was prepared by mixing 20 wt.% Pt/C (or RuO_2_, 5 mg) with a solution of 5 wt.% Nafion (40 μL) in ethanol (960 μL). The mixture was sonicated for 60 min. The catalyst ink (100 μL) was drop-cast on a Ni foam (1 × 1 cm^2^) and allowed to dry naturally in the air.

*Synthesis of NiMo*: The NiMo electrode was synthesized according to a previously reported method.^[1]^ Briefly, as-prepared NMO nanorods on Ni foam were placed in a tube furnace and heated under an Ar/H_2_ (50 sccm) environment. The temperature was ramped to 550 °C at a rate of 5 °C min^−1^ and held for 1 h. The NiMo on Ni foam was then allowed to cool down to room temperature.

*3. Materials Characterizations*

The phase and crystallinity of the as-synthesized samples were determined using X-ray diffraction (XRD, Rigaku SmartLab) with Cu *K*_α_ radiation at a scanning rate of 10° min^−1^ within a 2θ range from 10° to 70°. Raman spectra were collected on a confocal micro-Raman spectroscopy system (Renishaw, inVia) equipped with a 785-nm stream-line laser excitation source. Field emission scanning electron microscopy (SEM, Tescan MIRA) was used to investigate the morphology and size distribution of the samples. Scanning transmission electron microscopy (STEM, JEM-2100F STEM) equipped with an energy-dispersive spectrometer (EDS) was used to examine the lattice fringe and elemental distribution within the samples. Chemical bonding states and elemental composition were analyzed using X-ray photoelectron spectroscopy (XPS) equipped with monochromatic Al *K*_α_ radiation (ESCALAB 250 Xi, Thermo Fisher). The elemental compositions were further quantified by inductively coupled plasma optical emission spectroscopy (ICP-OES) using an Agilent 710 Series spectrometer. UV–vis spectra were recorded on a Jasco V780 spectrophotometer. Methanol oxidation products were analyzed by nuclear magnetic resonance (NMR) spectroscopy (400 MHz).

*4. Electrochemical Measurements*

The electrocatalytic properties of the samples for OER were evaluated using a standard three-electrode configuration in 1.0 M KOH electrolyte. A graphite rod and a Hg/HgO electrode were used as the counter and reference electrodes, respectively. To prepare the 1 M KOH seawater, KOH (5.61 g) was dissolved in the filtered seawater (100 mL) that was collected from Hung Hom Bay near the Hong Kong Polytechnic University and allowed to equilibrate for 1 h. The supernatant (pH = ~13.7) was then used as the electrolyte for seawater oxidation reactions. All data were acquired using a Princeton multichannel electrochemical station. All potentials in this work were calibrated against the reversible hydrogen electrode (RHE, *E*_RHE_ = *E*_Hg/HgO_ + 0.059 × pH + 0.098). Prior to OER testing, the working electrodes were cycled between 1.124 and 1.624 V for 100 cycles to achieve a stable cyclic voltammogram (CV). Linear sweep voltammetry (LSV) was then conducted from 1.2 to 1.9 V at a scan rate of 2 mV s^−1^. The MOR activity of the samples was evaluated using the same method in 0.1 M methanol + 1 M KOH. All polarization curves were corrected for ohmic losses with 90 % *i*R compensation.

Electrochemical impedance spectroscopy (EIS) was performed at 1.45 V across a frequency range between 100 kHz and 0.01 Hz with an amplitude of 10 mV. *In situ* EIS measurements were conducted within the same frequency range but at various potentials. Electrochemical surface area (ECSA) was estimated by measuring double-layer capacitances (*C*_dl_) from the CVs collected in a non-Faradaic region between 1.254 and 1.354 V at various scan rates of 20, 40, 60, 80, and 100 mV s^−1^ using the equation:

$ECSA=\frac{C_{\mathrm{dl}}}{C_{s}}$ (1)

where *C*_s_ is the specific capacitance of the sample, which is usually 0.04 mF cm^−2^ in an alkaline solution.

Turnover frequency (TOF) values were calculated using the equation:

$TOF=\frac{i}{nFN_{\mathrm{site}}}$ (2)

where *i* is the current at a given OER overpotential, *n* is the transferred electron numbers to produce one O_2_ molecule (4 for OER), *F* is the Faradaic constant (96,485 C mol^−1^), and *N*_site_ is the total number of metal sites (mol) on electrodes determined by ICP-OES.

Apparent electrochemical activation enthalpy (*E*_a_) for OER was estimated from the slope of the Arrhenius plot using the following equation:

$\frac{\partial(lnj_{0})}{\partial(\frac{1}{T})}=\frac{-E_{a}}{R}$ (3)

where *R* is the universal gas constant, *T* is the temperature, and *j*_0_ is the kinetic current density.

The Faradaic efficiency (FE) was determined using a gas collection method in a gas-tight H-cell. The working electrode was operated at a constant current density of 100 mA cm^−2^ in either 1 M KOH or seawater electrolyte. The evolved O_2_ gas product was collected using a graduated tube with one sealed end. The FE was calculated based on the total charge passed through the electrode (*Q*) and the amount of O_2_ produced (*n*) using the following equation:

$\mathrm{FE}=\frac{4F ⨯ n}{Q}$ (4)

Immediately following the FE measurements, the presence of hypochlorite ions (ClO^−^) generated during OER was investigated using iodide titration. Hypochlorite reacts with I^−^, leading to an instantaneous color change and a distinct absorption peak at around 358 nm. Briefly, after the FE test, 10 mL of electrolyte was pipetted from the reaction cell. The pH of the extracted electrolyte was adjusted to 5 using 0.5 M H_2_SO_4_, followed by the addition of 0.5 M KI solution (5 mL). The solution was then titrated with 0.01 M thiosulfate solution dropwise. Subsequently, a UV−vis spectrum was collected using a spectrophotometer (Agilent Cary-60) within the wavelength range of 300 to 600 nm. The presence of hypochlorite was confirmed by monitoring the peak intensity at 358 nm.

*In situ* Raman spectroscopy was employed to monitor the electrode surface using the OER process. The measurements were conducted on a Raman spectrometer equipped with a 785 nm streamline laser excitation and a working station (CHI760E). To minimize potential sample damage or interference, each spectrum was collected with a 10-s exposure time and a low laser intensity of 1.5 mW. Two accumulations were performed for each spectrum to enhance signal quality. For these measurements, a standard three-electrode configuration was used with a Hg/HgO reference electrode and a Pt wire counter electrode. LSV scans were conducted at a scan rate of 0.25 mV s^−1^ to allow for sufficient time for Raman spectra acquisition. *Quasi-operando* Raman spectroscopy measurements were used to probe the electrode surface under conditions closer to real-world OER application. The working electrodes were first oxidized at a fixed potential of 1.50 V. Following the addition of methanol, Raman spectra were recorded at different reaction times.

Quasi-*in-situ* UV−vis spectra were collected using a spectrophotometer (Jasco V780) and a potentiostat (CHI760E) to identify the presence of MoO_4_^2−^ during the reconstruction of Ni(OH)_2_/NMO. Using ammonium molybdate ((NH_4_)_6_Mo_7_O_24_∙4H_2_O) as a reference for MoO_4_^2−^ detection, standard absorption spectra were first obtained for solutions containing 0, 0.001, 0.005, and 0.01 mM ((NH_4_)_6_Mo_7_O_24_∙4H_2_O). The characteristic absorption peaks for MoO_4_^2−^ were identified at 209 and 231 nm. To detect MoO_4_^2−^ during the OER process, UV−vis absorption spectra of the electrolytes were collected at different reaction times. A two-electrode configuration was used, with Ni(OH)_2_/NMO as the anode and a Pt wire as the cathode using 3 mL of the reaction reagent as the electrolyte. An applied potential of 2.5 V was maintained using chronoamperometry for 1 min, and the entire electrolyte was extracted from the cell and analyzed using UV–vis spectroscopy. A fresh electrolyte (3 mL) was added to the cell for subsequent measurements.

*5. Flow Cell Measurements*

For hydrogen evolution reaction (HER) evaluation, the NiMo cathode was coupled with a scaled-up Ni(OH)_2_/NMO anode (1 cm × 1 cm) to form a full-cell water-splitting system. For comparison, commercial RuO_2_ and Pt/C on Ni foam were used as a benchmark for the anode and cathode, respectively. The water-splitting reaction was conducted using a Princeton multichannel electrochemical station equipped with a membrane electrode assembly (MEA) electrolyzer and a peristaltic pump. The MEA electrolyzer consisted of separate flow fields for the anode and cathode, each with an active surface area of 1 cm^2^. These flow fields were responsible for the continuous delivery of aqueous electrolytes over the electrode surfaces. Inside the MEA, the cathode and anode were positioned on their respective flow-field plates and physically separated by an anion exchange membrane (AEM, Fumasep, Fuel Cell Store). The MEA was then uniformly compressed to ensure proper contact and sealing between the electrodes and the AEM. Following the MEA assembly, alkaline seawater electrolyte or hybrid seawater electrolyte (0.1 M methanol + 1 M KOH + seawater) was continuously pumped through the anode and cathode compartments at a constant flow rate of 5 mL min^−1^. For performance evaluation, chronopotentiometric tests were conducted by applying constant current densities of 0.1, 0.5, and 1.0 A cm^−2^ to the anode. The corresponding cell voltage was continuously monitored and recorded throughout each test.

*6. Calculations of Energy Cost and Saving for the H_2_ Production*

The electrical energy required to produce a specific amount of H_2_ in the electrochemical cells of NiMo||Ni(OH)_2_/NMO and Pt/C||RuO_2_ was calculated using Equation (5):

*E* = *U* × *n* × *F* (5)

where *U* is the cell voltage, *n* is the amount of produced H_2_ in mol, and *F* is the Faraday constant (96,485 C mol^−1^). The percentage of energy saved during H_2_ generation using the NiMo||Ni(OH)_2_/NMO cell compared to the Pt/C||RuO_2_ cell was calculated using Equation (6):

$P_{energy saving}=\frac{U_{Pt/C||RuO2}-U_{NiMo||Ni(OH)2/NMO}}{U_{Pt/C||RuO2}}\times100\%$ (6)

where *U*_Pt/C||RuO2_ and *U*_NiMo||Ni(OH)2/NMO_ are the cell voltage of Pt/C||RuO_2_ cell and NiMo||Ni(OH)_2_/NMO cell at a specific current density, respectively.

*7. Theoretical Calculations*

The Vienna Ab initio Simulation Package (VASP) code^[2-4]^ was employed for electronic structure calculations. Projected augmented-wave (PAW) potentials and Perdew−Burke−Enzerhof (PBE) functional within a generalized gradient approximation (GGA) framework were used to accurately represent electron–ion and exchange-correlation interactions.^[5-7]^ An energy cutoff of 480 eV and stringent force convergence criteria of 0.03 eV·Å^−1^ were applied to ensure precision. A 12-Å vacuum layer in the *z*-direction was added to the model to minimize the influence of neighboring layers through van der Waals forces. The Monkhorst-Pack scheme with a 2 × 6 × 1 k-mesh was employed for the Brillouin zone sampling.^[8]^ The Bader charge analysis^[9-11]^ was employed to determine the partial charges of individual atoms within the materials. The Gibbs free energy (Δ*G*) was calculated using Equation (7):

Δ*G* = Δ*E* + Δ*ZPE* – *T*Δ*S* + Δ*G*_U_ + Δ*G*_pH_ (7)

where Δ*E* is reaction energy change, Δ*ZPE* is zero-point energy, *T* is temperature (set to 298.15 K in this study), Δ*S* is entropy change, Δ*G*_U_ is electrode potential bias, and ΔG_pH_ is pH corrections. Here, Δ*ZPE* was calculated by summing the vibrational frequencies (*ν*) of each normal mode using Equation (8):

$\Delta ZPE=\frac{1}{2}h\sum v$ (8)

where *h* is the Planck’s constant. Δ*G*_U_ was calculated using Equation (9):

Δ*G* = −*neU* (9)

where *U* refers to the applied potential (*vs*. RHE), *n* is the number of proton–electron pairs transferred, and *e* is the elementary charge. Δ*G*_pH_ is the correction for the free energy associated with H^+^, which can be calculated using Equation (10):

Δ*G*_pH_ *= −k*_B_*T*ln[H^+^] (10)

where *k*_B_ is the Boltzmann constant.

**Supporting Figures**


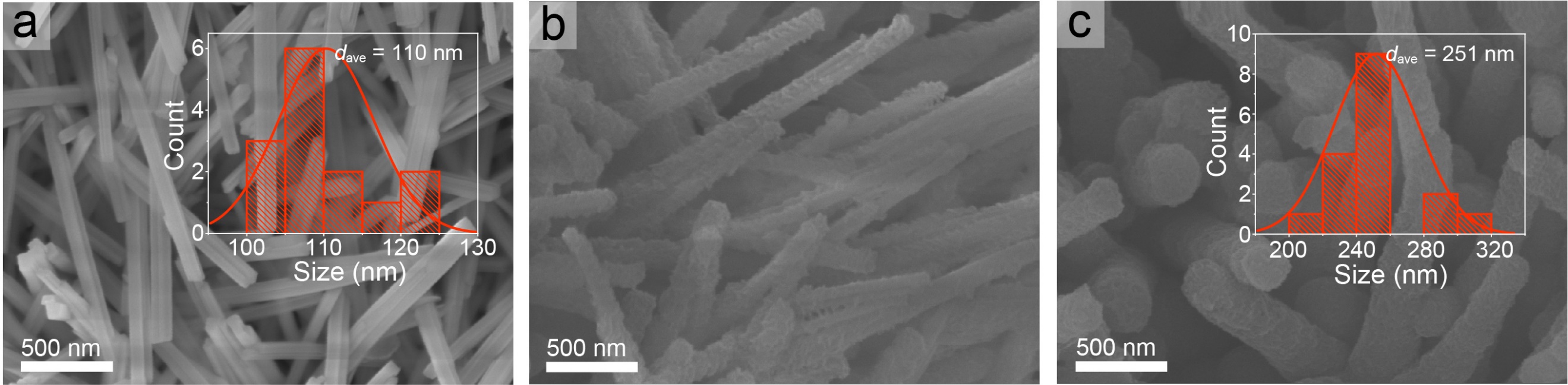


**Figure S1.** SEM images of (a) NiMoO_4_ and (b,c) Ni(OH)_2_/NMO prepared using different electrodeposition durations: (a) 0 min, (b) 5 min, and (c) 15 min. Insets in (a) and (c) are histograms showing the distribution of diameter.


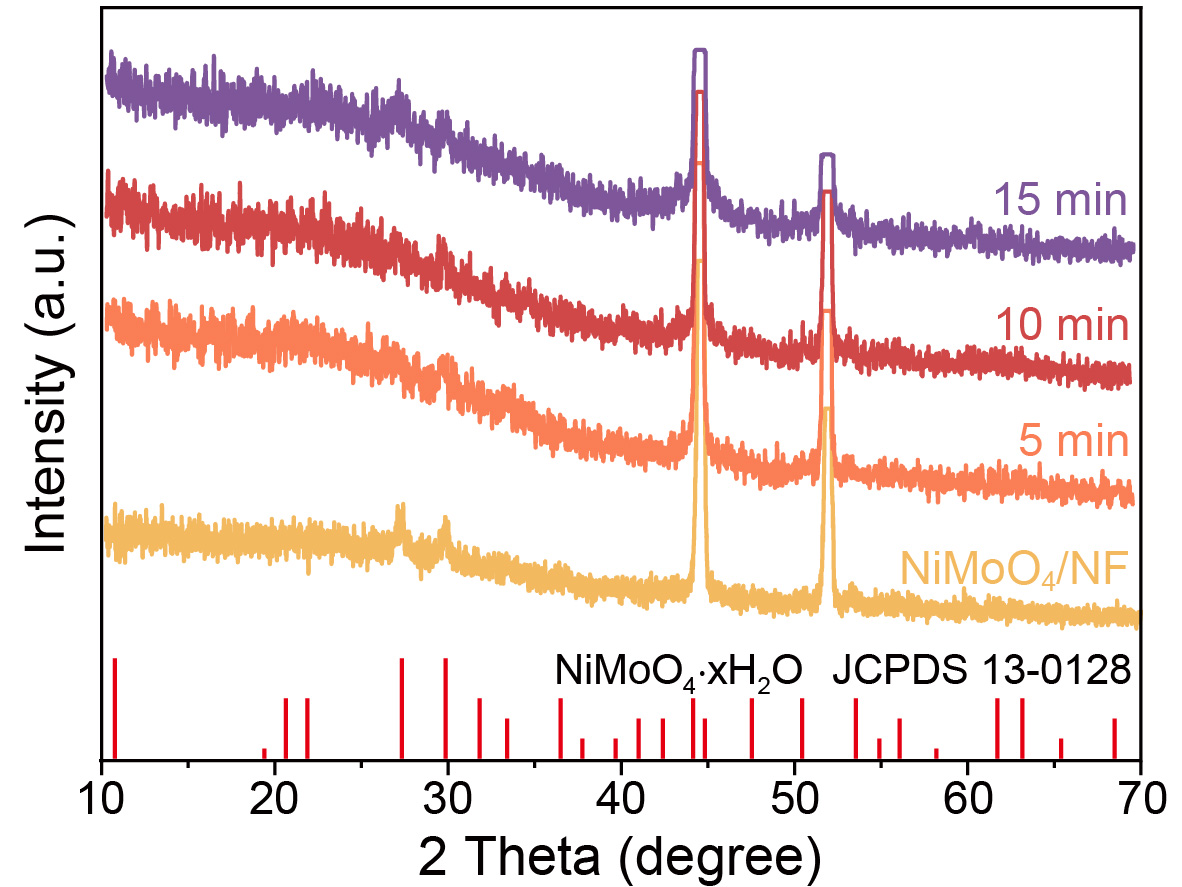


**Figure S2.** XRD patterns of NiMoO_4_ and Ni(OH)_2_/NMO samples synthesized using different electrodeposition durations from 5 to 15 min.


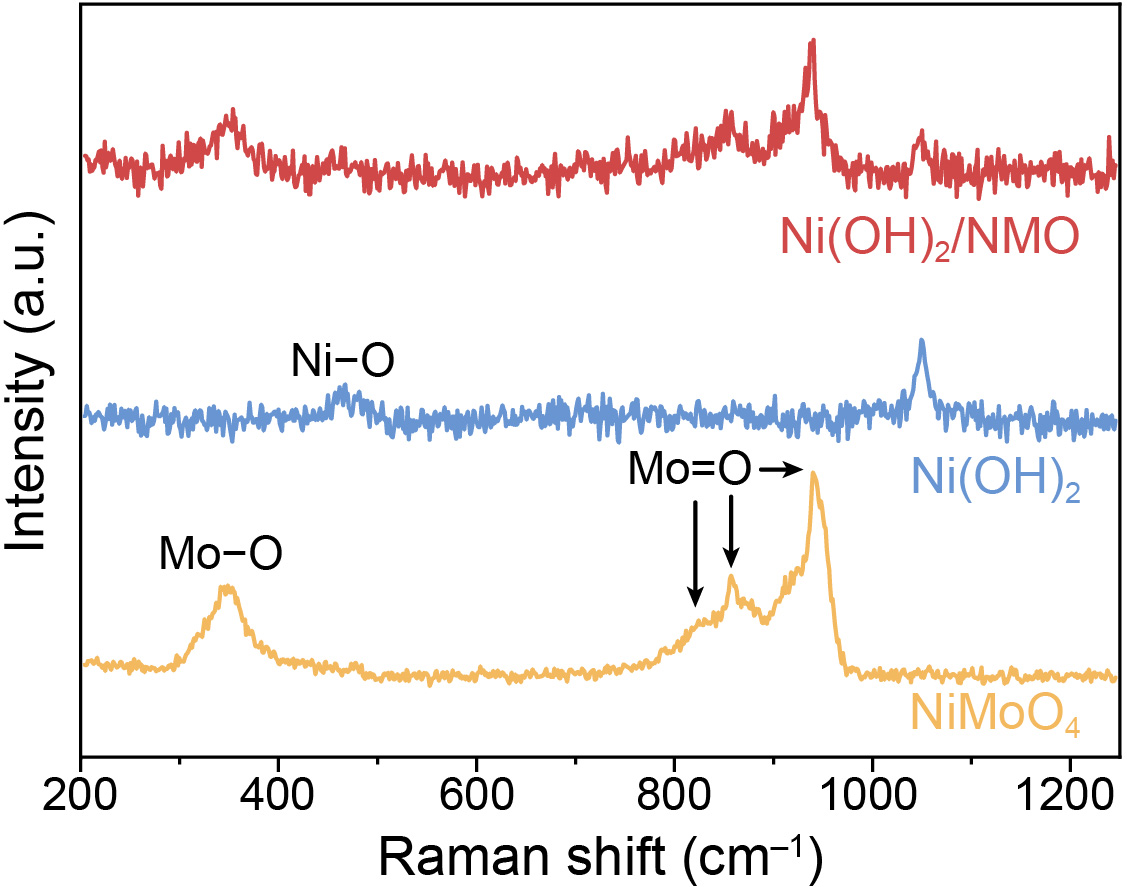


**Figure S3.** Raman spectra of NiMoO_4_, Ni(OH)_2_, and Ni(OH)_2_/NMO.


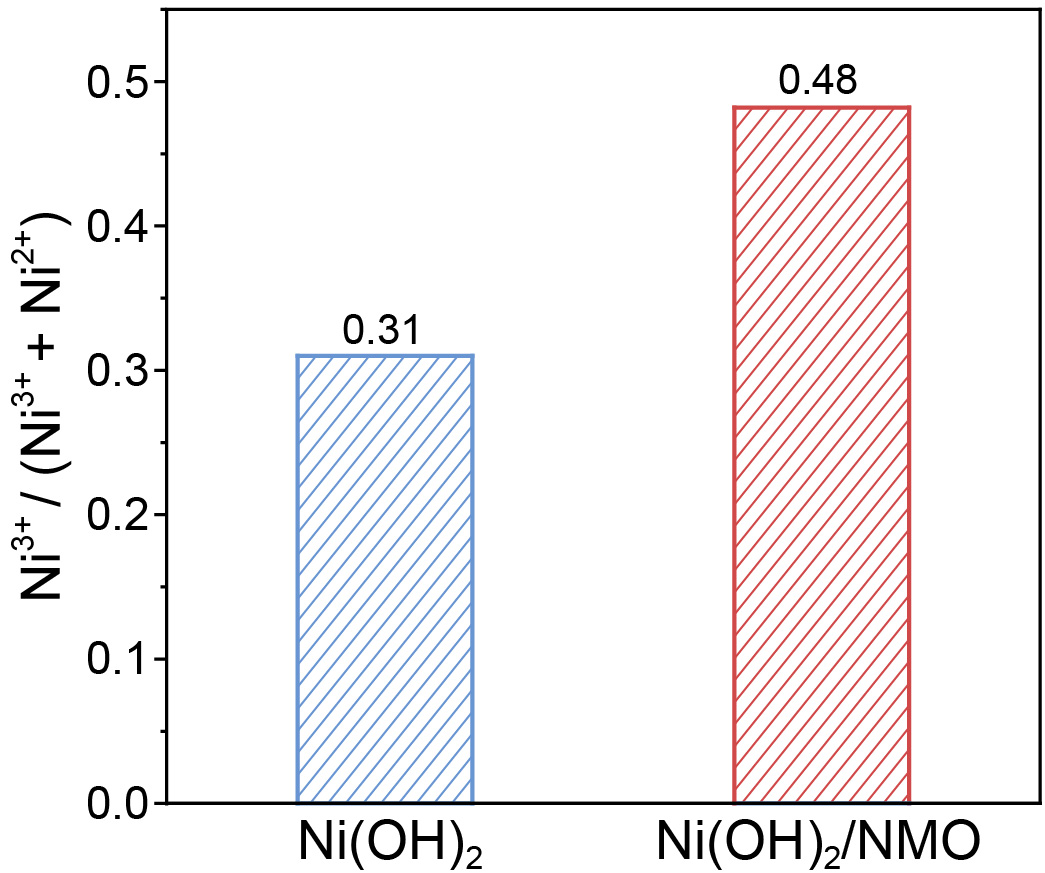


**Figure S4.** Ni^3+^/(Ni^3+^+Ni^2+^) peak area ratio of Ni(OH)_2_ and Ni(OH)_2_/NMO.


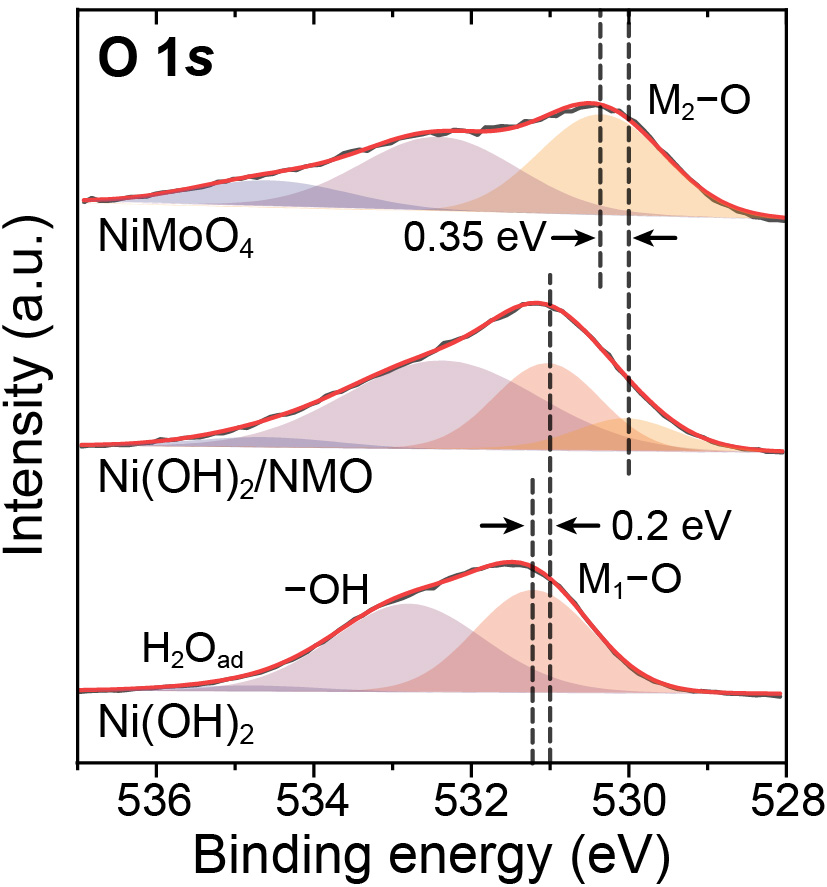


**Figure S5.** XPS O 1*s* spectra of NiMoO_4_, Ni(OH)_2_, and Ni(OH)_2_/NMO.


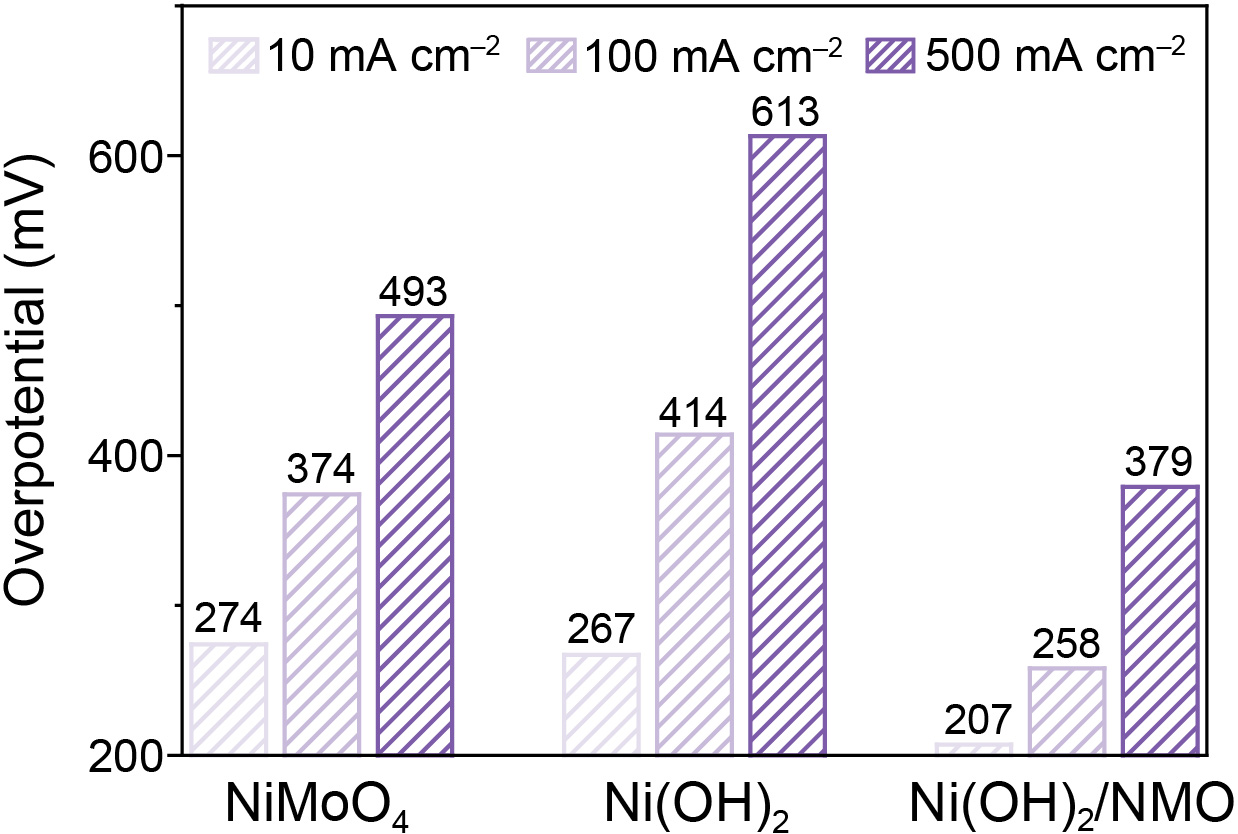


**Figure S6.** OER performance of NiMoO_4_, Ni(OH)_2_, and Ni(OH)_2_/NMO: Comparison of OER overpotentials required to achieve current densities of 10, 100, and 500 mA cm^−2^.


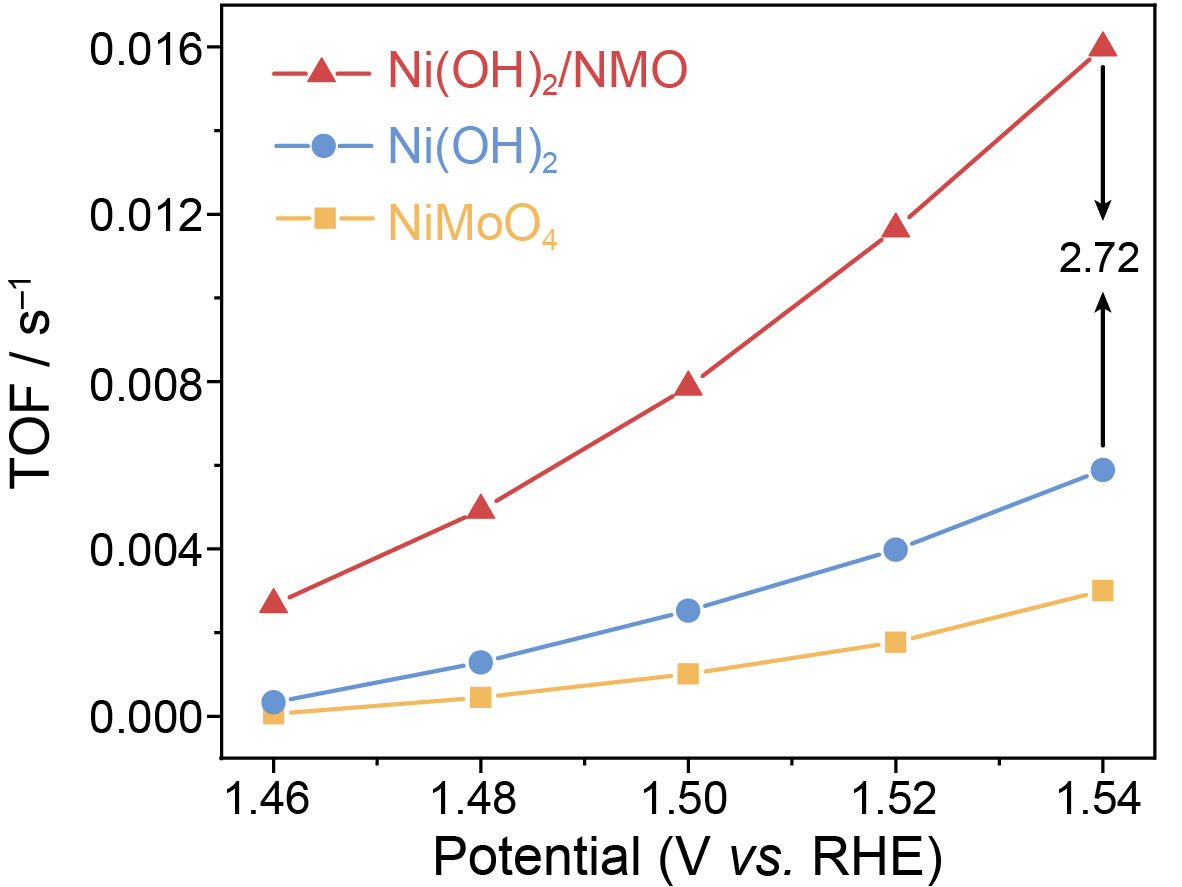


**Figure S7.** TOF values of NiMoO_4_, Ni(OH)_2_, and Ni(OH)_2_/NMO in a potential range from 1.46 to 1.54 V in 1 M KOH.


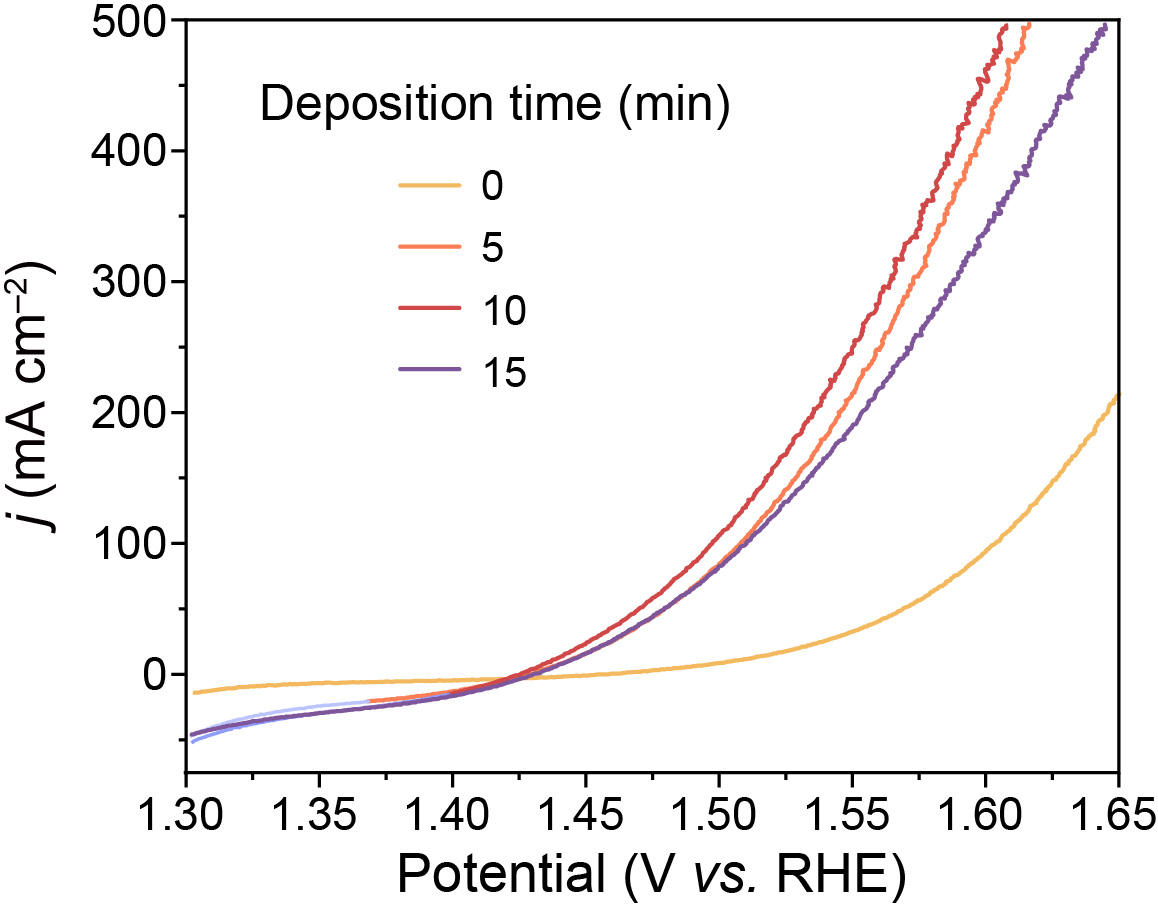


**Figure S8.** LSVs of Ni(OH)_2_/NMO samples synthesized using various electrodeposition durations in 1 M KOH.


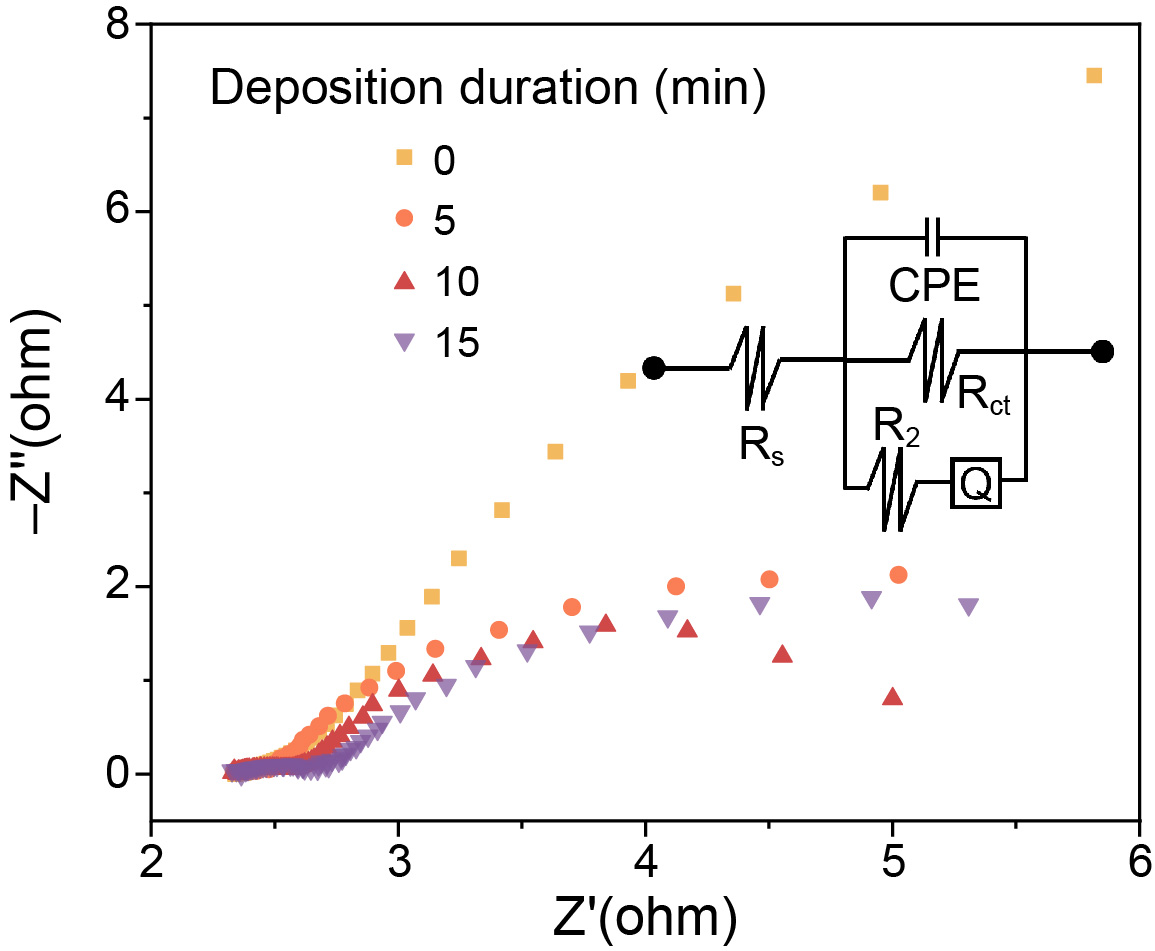


**Figure S9.** Nyquist plots of Ni(OH)_2_/NMO composites prepared using various electrodeposition durations. Inset is an equivalent circuit, where R_s_ stands for the electrolyte resistance, CPE represents double-layer capacitance, and R_ct_ (low-frequency range) is related to the interfacial charge transfer reaction. CPE, Q, and R_2_ (high-frequency range) are associated with the dielectric properties, inductance, and the resistance of the electrode itself, respectively. The estimated values are summarized in **Table S3**.


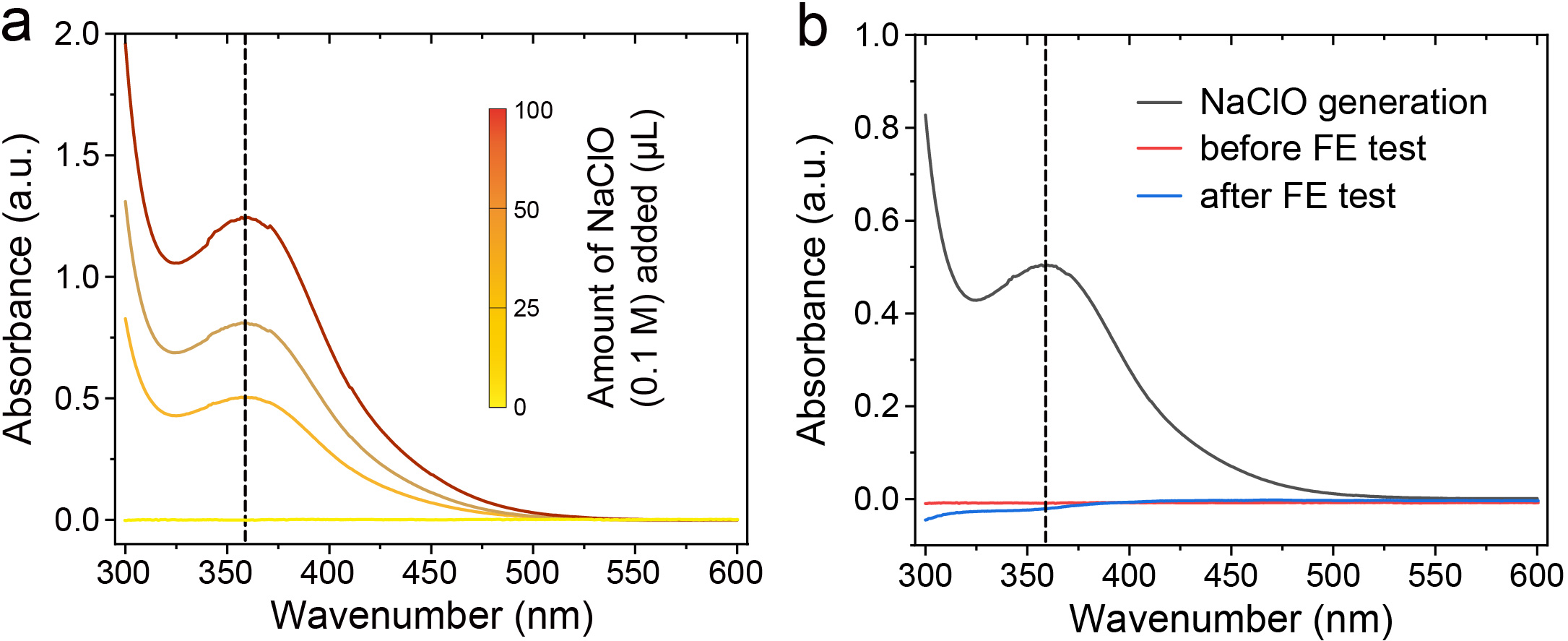


**Figure S10.** UV–vis spectra of (a) iodide titration with various NaClO concentrations and (b) electrolytes before and after the FE test.


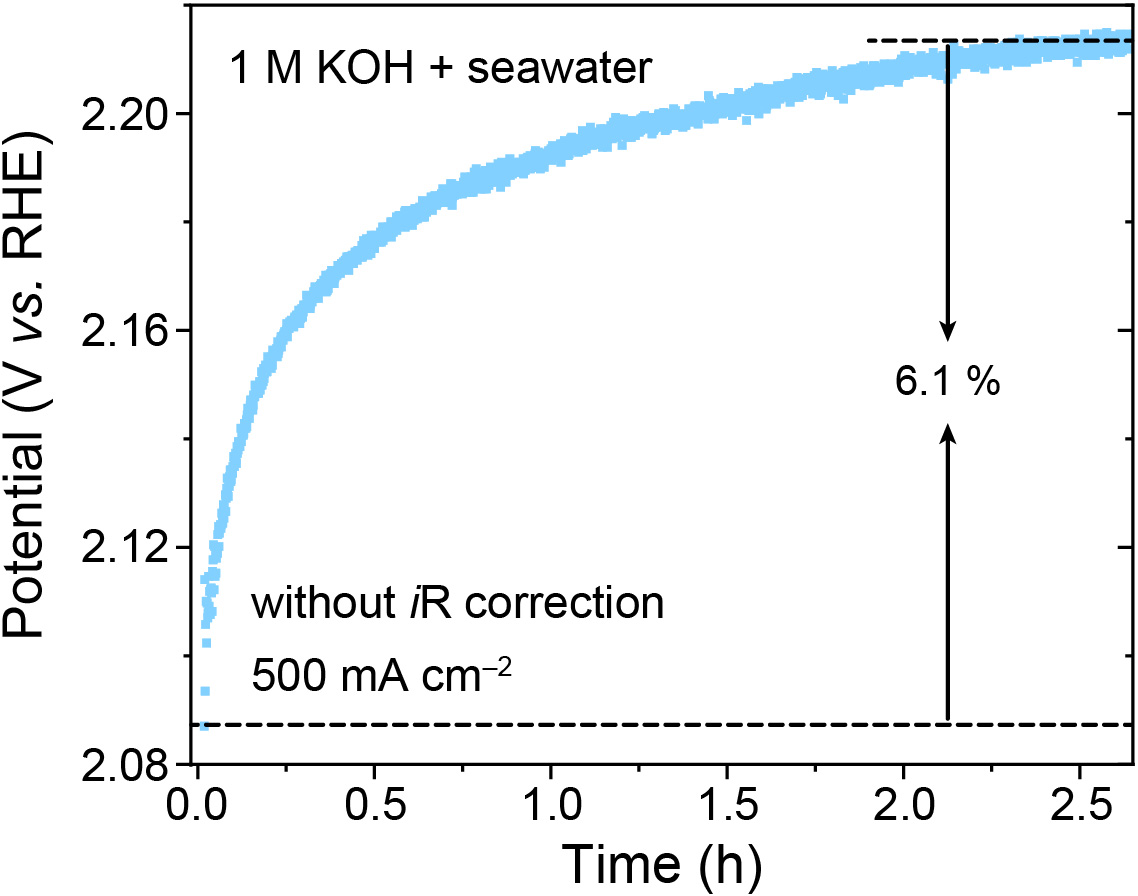


**Figure S11.** Chronopotentiometric curve of Ni(OH)_2_ in 1 M KOH + seawater.


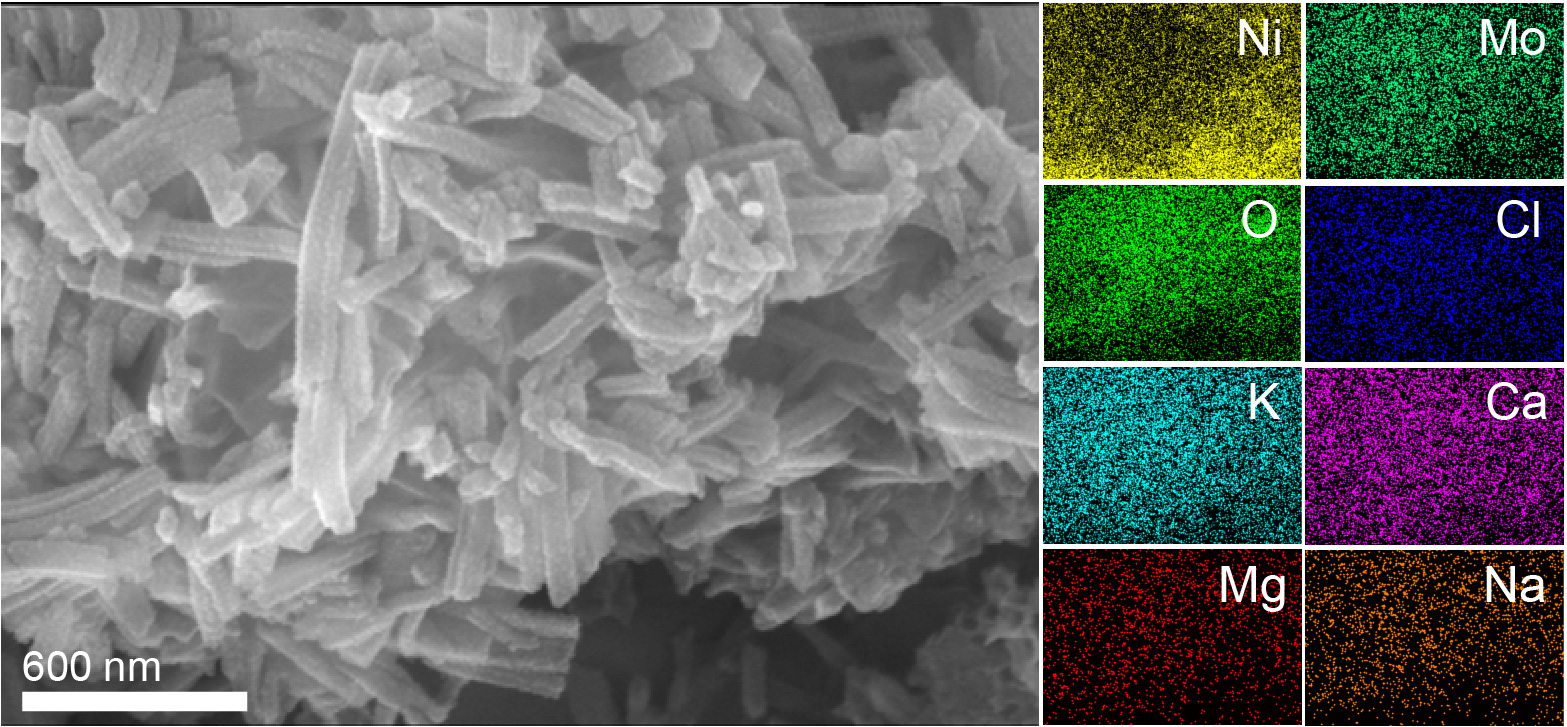


**Figure S12.** SEM image and the corresponding EDS mapping images of Ni(OH)_2_/NMO after 600-h chronopotentiometry in 1 M KOH + seawater.


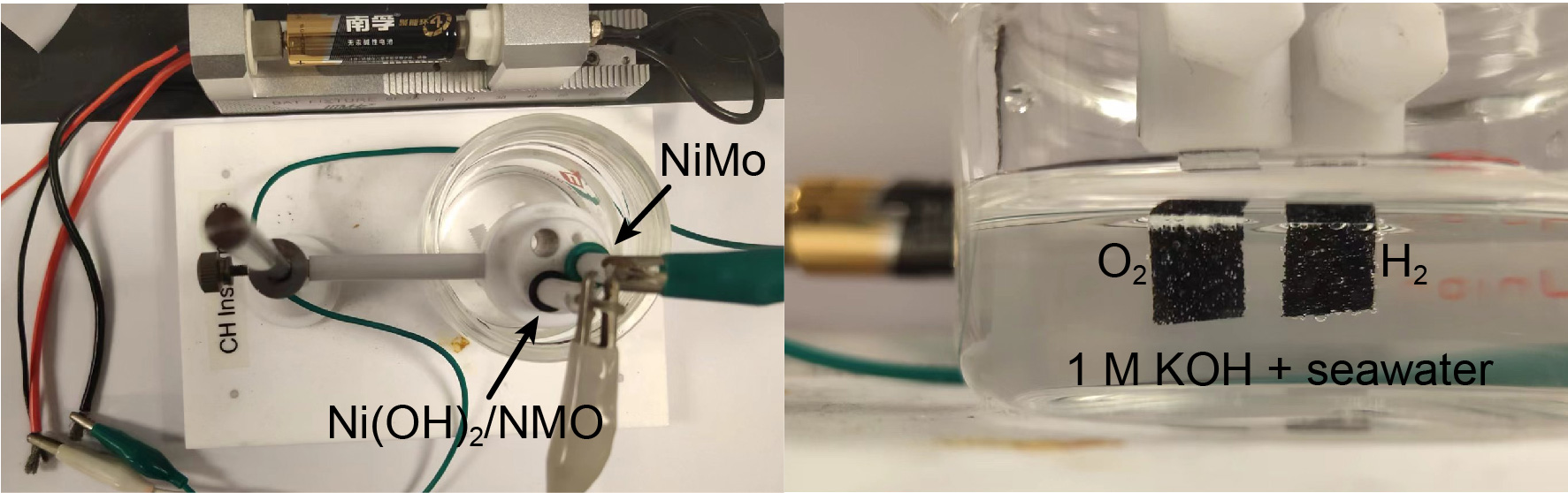


**Figure S13**. A photo of NiMo||Ni(OH)_2_/NMO two-electrode electrolysis system driven by a commercial 1.5-V battery.

NiMo cathode was coupled with Ni(OH)_2_/NMO anode to form a seawater-splitting system. A commercial 1.5-V battery was used to power the device setup. The continuous and vigorous production of bubbles at both the cathode and anode was observed (**Figure S13**), which were assigned to H_2_ and O_2_, respectively, confirming its feasibility for practical application.


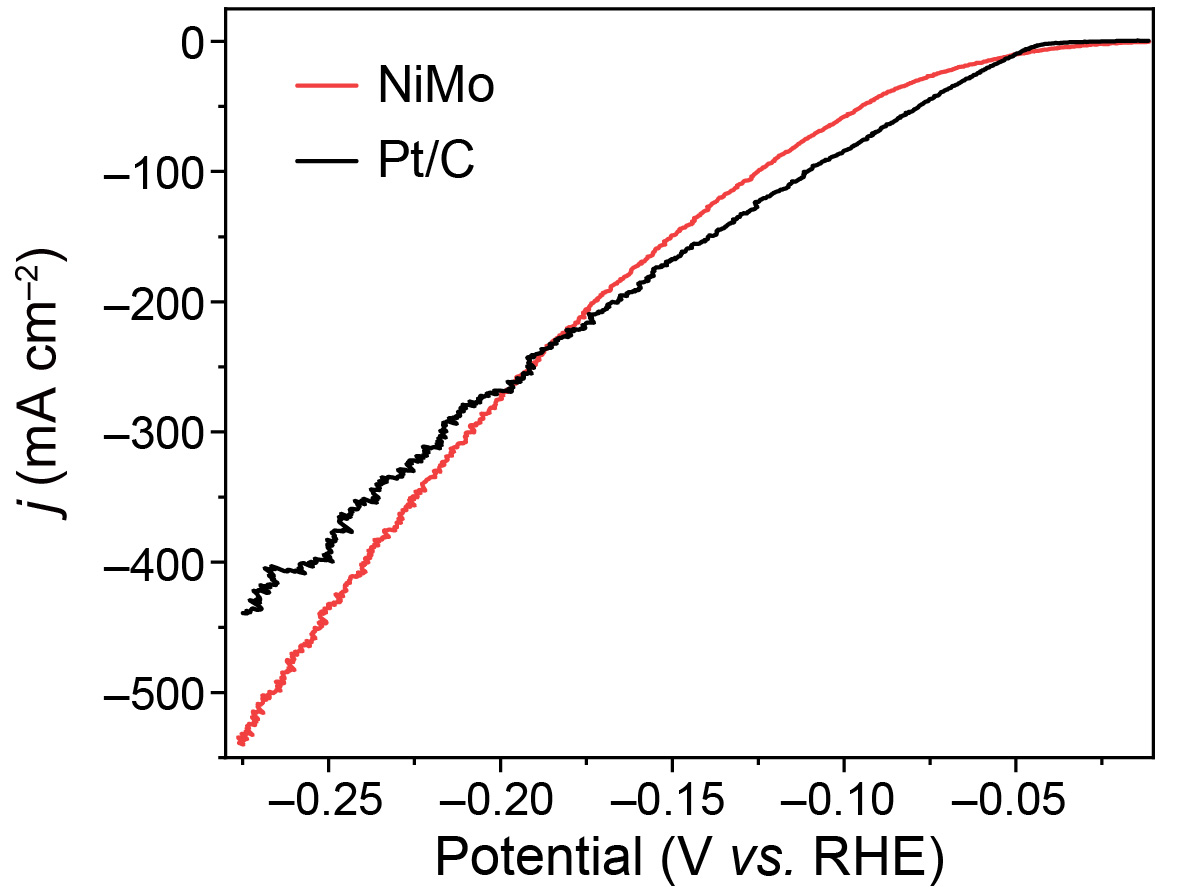


**Figure S14.** LSV curves of NiMo and commercial Pt/C catalysts in 1 M KOH.


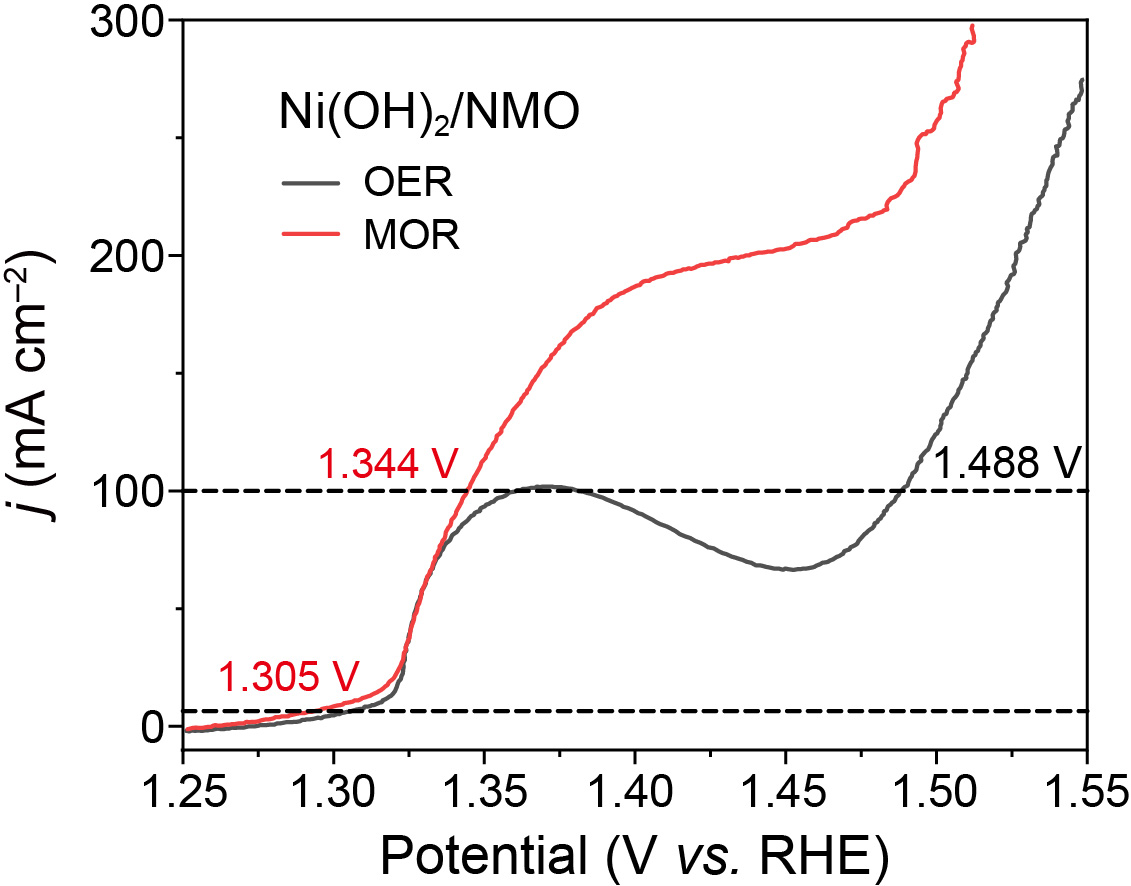


**Figure S15.** LSV curves of Ni(OH)_2_/NMO during OER (in 1 M KOH) and MOR (in 1 M KOH + 0.1 M methanol).


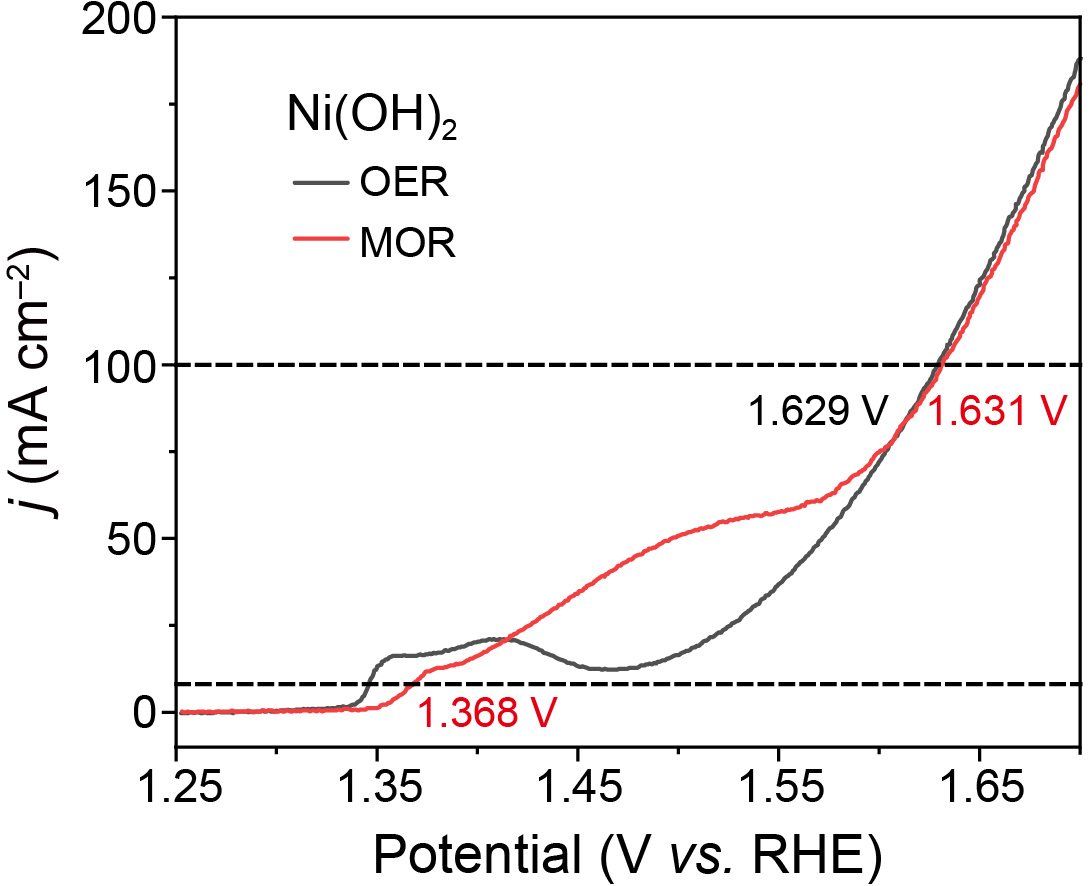


**Figure S16.** LSV curves of Ni(OH)_2_ during OER (in 1 M KOH) and MOR (in 1 M KOH + 0.1 M methanol).


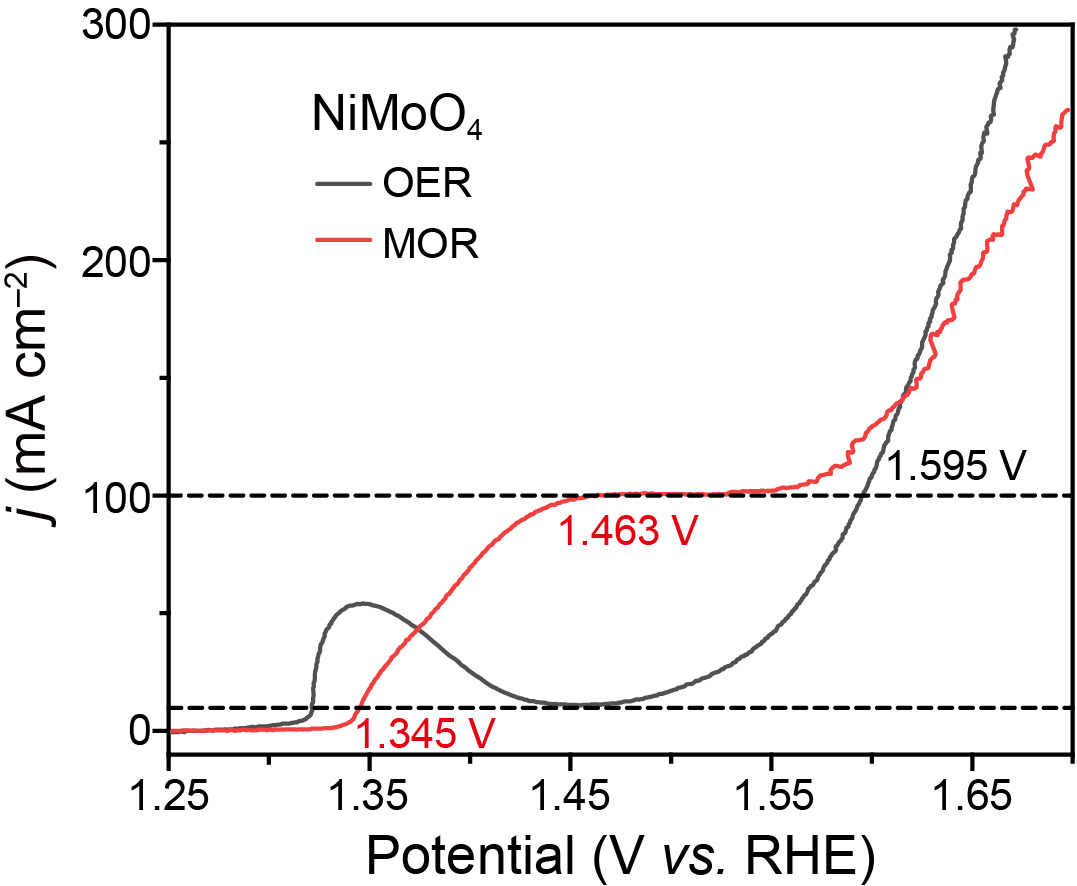


**Figure S17.** LSV curves of NiMoO_4_ during OER (in 1 M KOH) and MOR (in 1 M KOH + 0.1 M methanol).


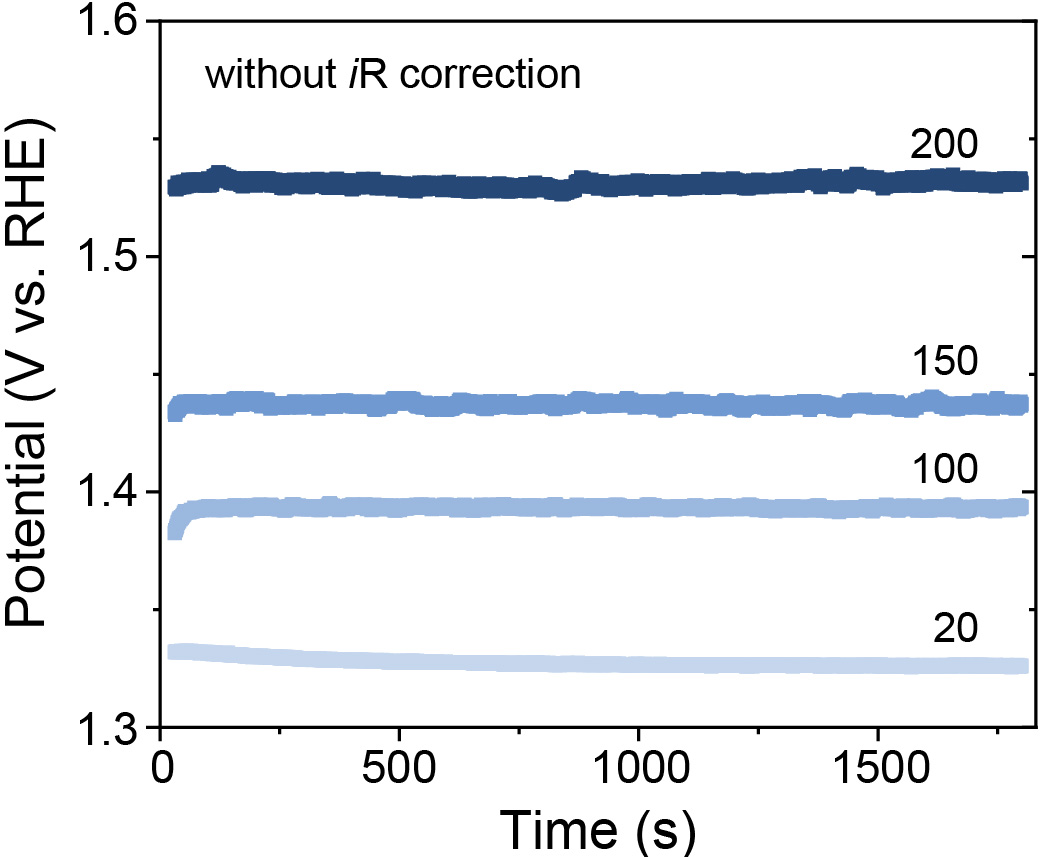


**Figure S18.** Chronopotentiometric curves of 0.5-h MOR at current densities from 20 to 100, 150, and 200 mA cm^−2^.


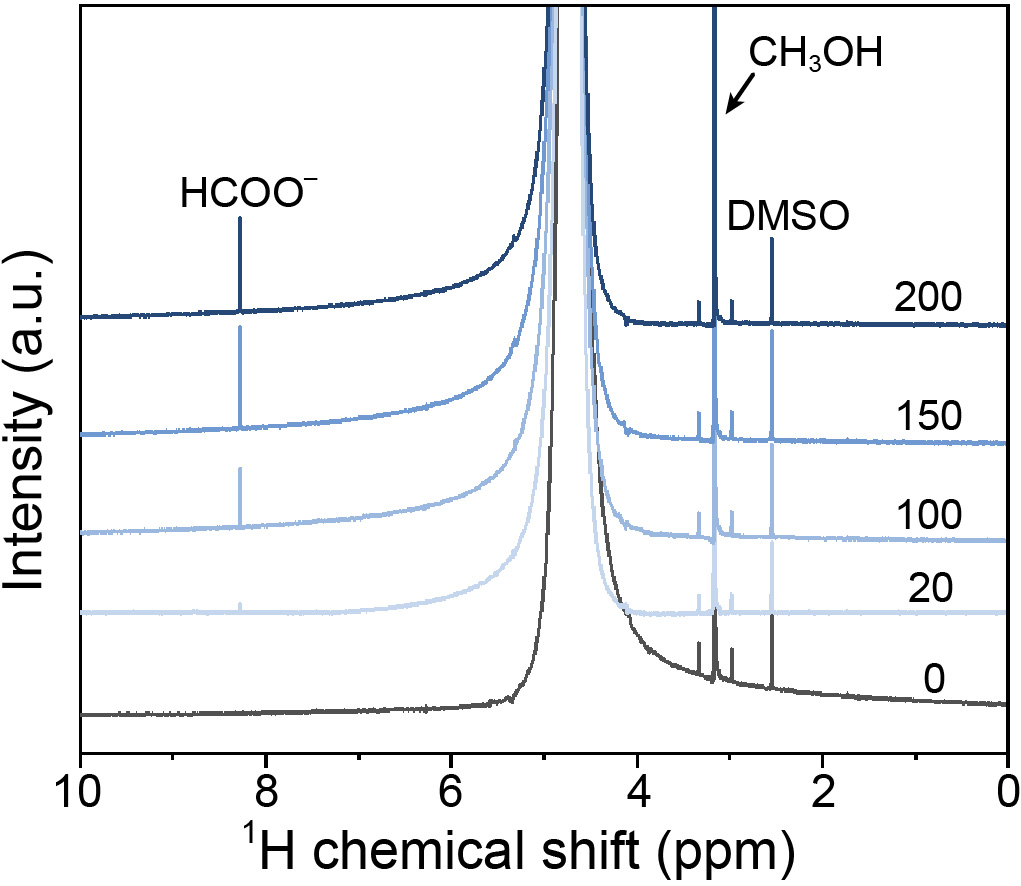


**Figure S19.** NMR spectra of electrolytes after 0.5-h MOR at current densities from 0 to 20, 100, 150, and 200 mA cm^−2^.


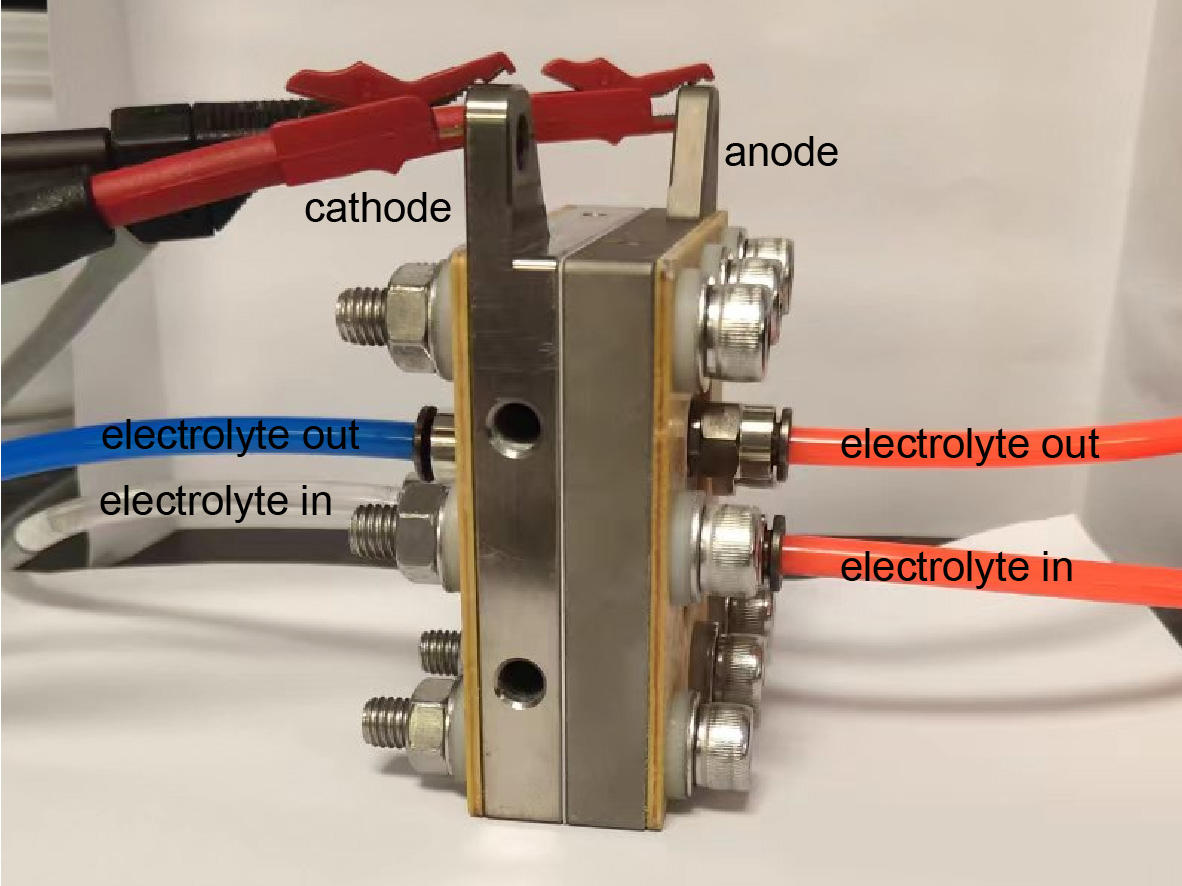


**Figure S20.** A photo of the flow cell water-splitting system.


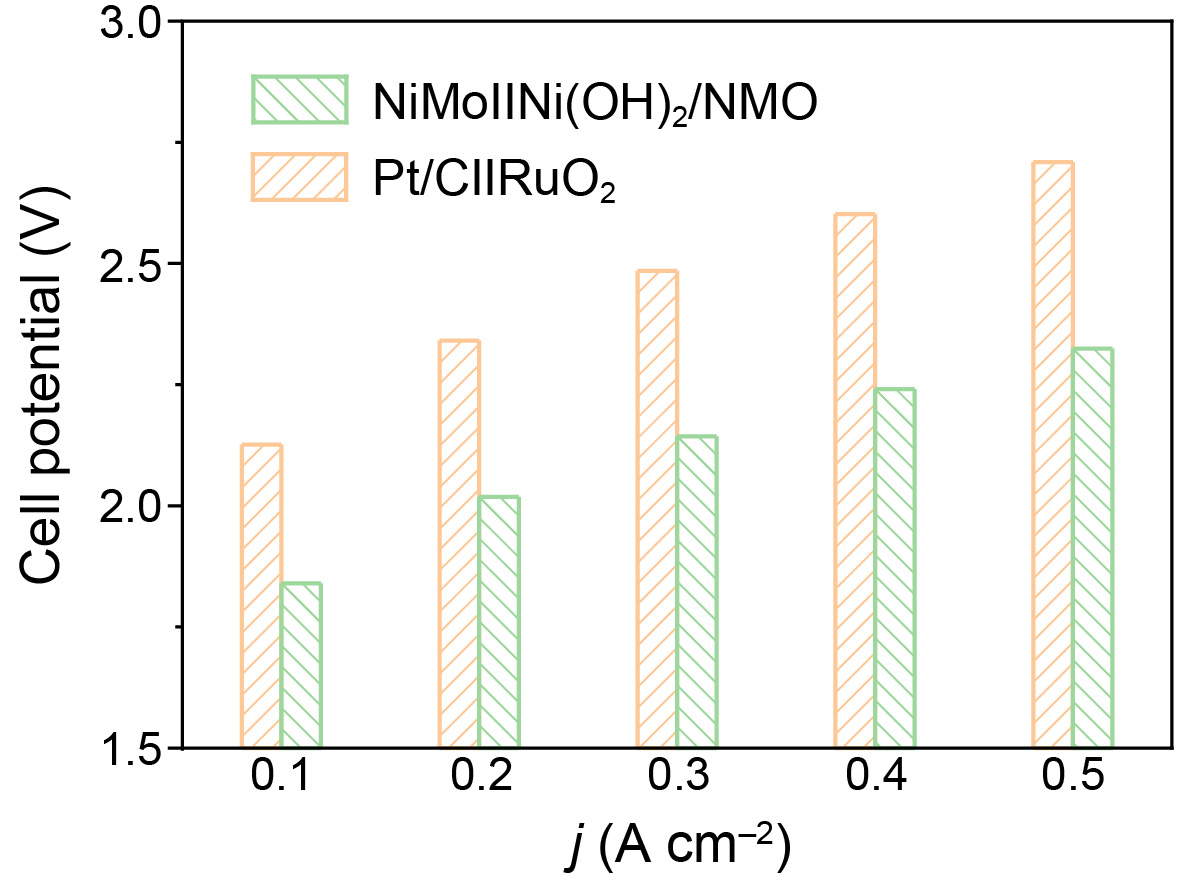


**Figure S21.** Comparison of cell potentials of NiMo||Ni(OH)_2_/NMO and Pt/C||RuO_2_ required to achieve current densities of 0.1, 0.2, 0.3, 0.4, and 0.5 A cm^−2^.


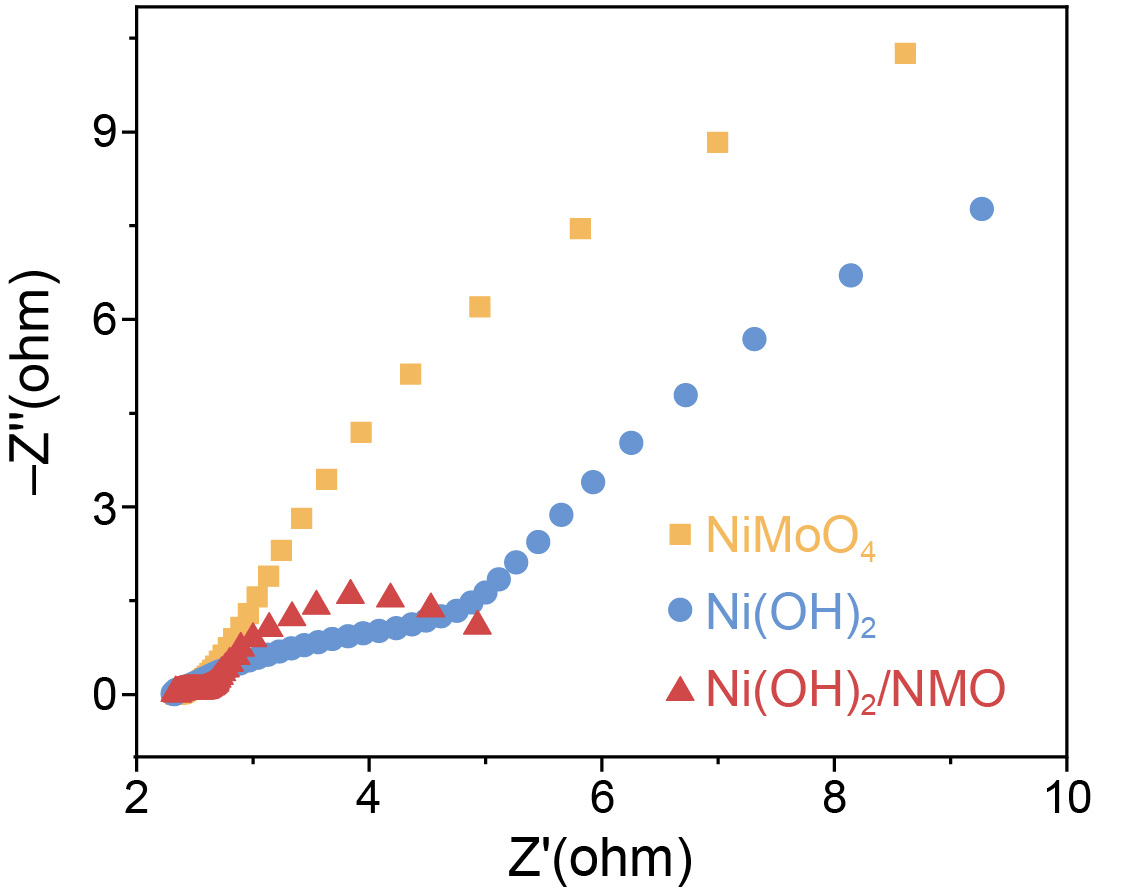


**Figure S22.** Nyquist plots of Ni(OH)_2_, NiMoO_4_, and Ni(OH)_2_/NMO in 1 M KOH.


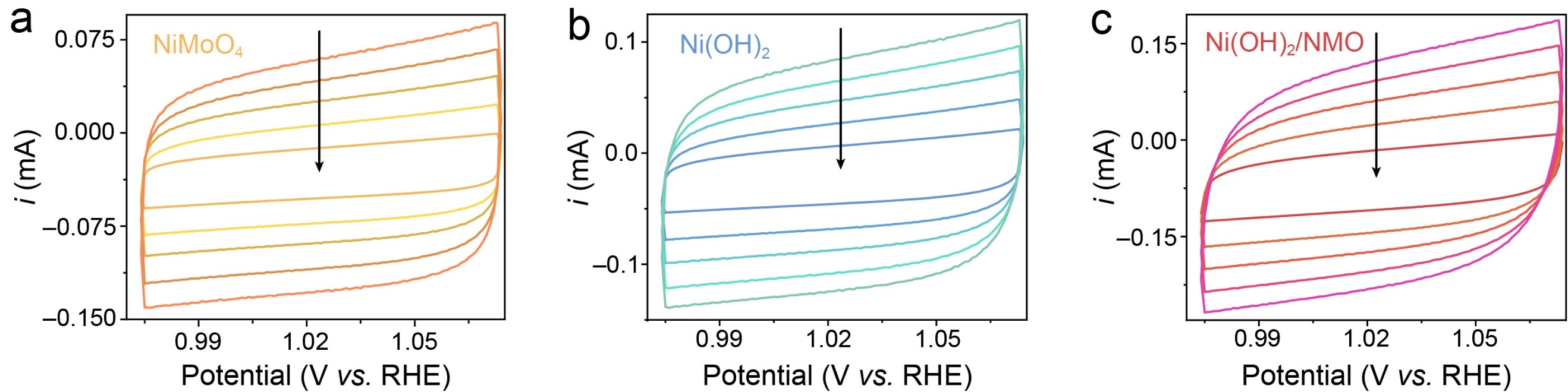


**Figure S23.** Cyclic voltammograms measured in a non-Faradaic region at various scan rates. (a) NiMoO_4_, (b) Ni(OH)_2_, and (c) Ni(OH)_2_/NMO. The scan rate decreased from 250 to 50 mV s^−1^.


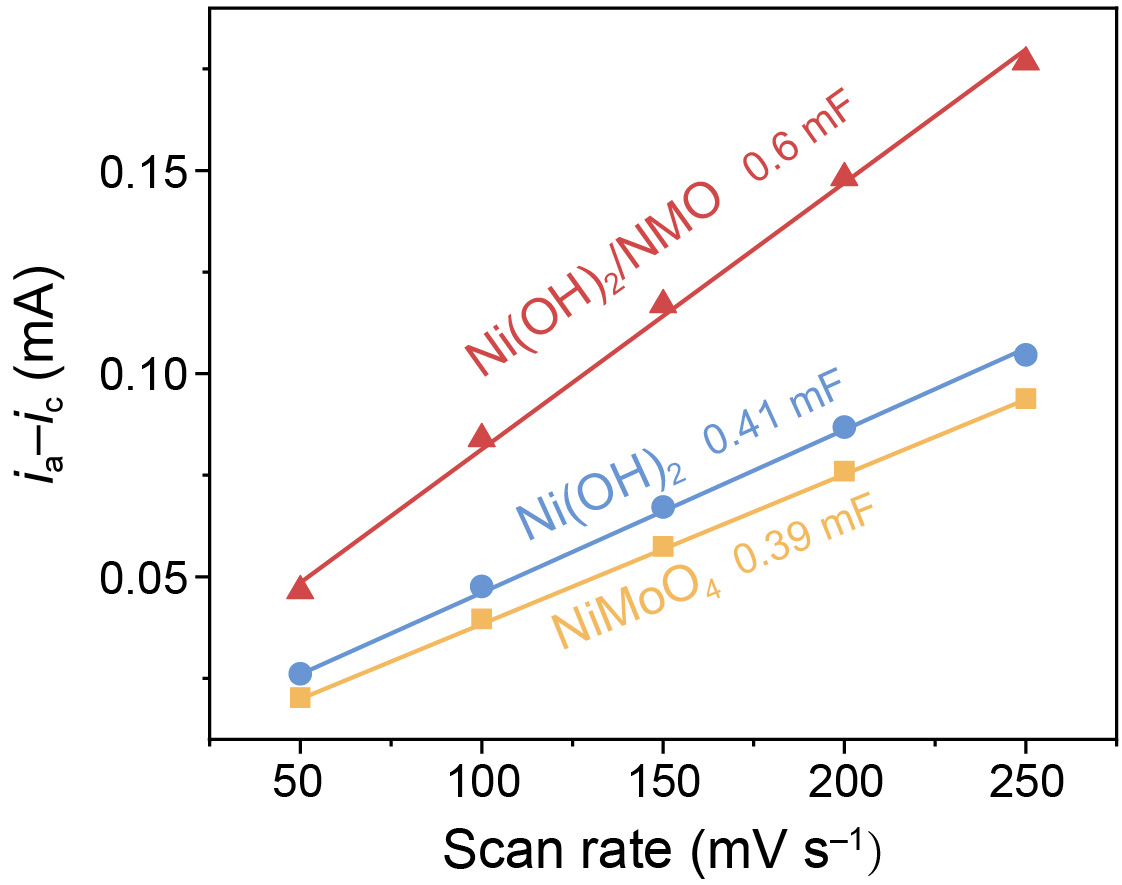


**Figure S24.** Linear fitting of double-layer capacitance (*C*_dl_) against CV scan rate for the estimation of electrochemically active surface area (ECSA) of Ni(OH)_2_, NiMoO_4_, and Ni(OH)_2_/NMO.


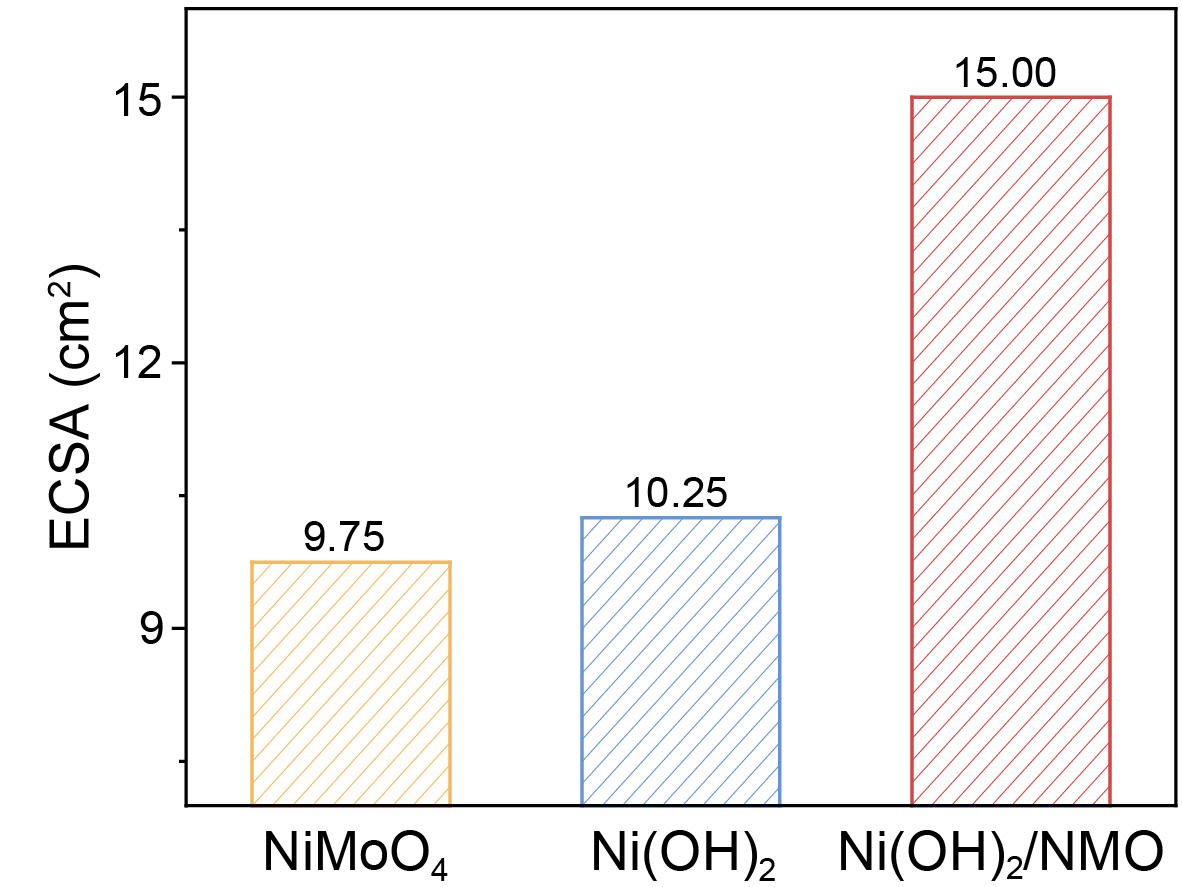


**Figure S25.** ECSA of Ni(OH)_2_, NiMoO_4_, and Ni(OH)_2_/NMO.


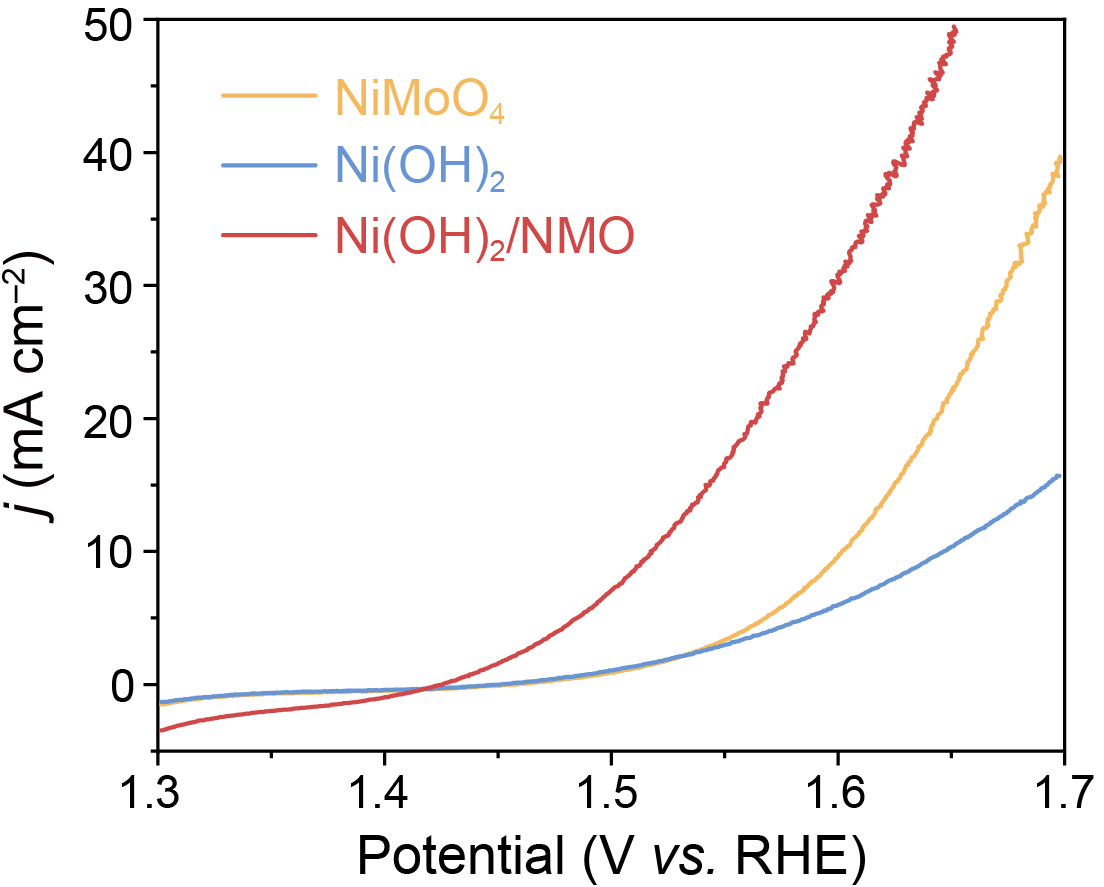


**Figure S26.** ECSA-normalized LSV curves of Ni(OH)_2_, NiMoO_4_, and Ni(OH)_2_/NMO.


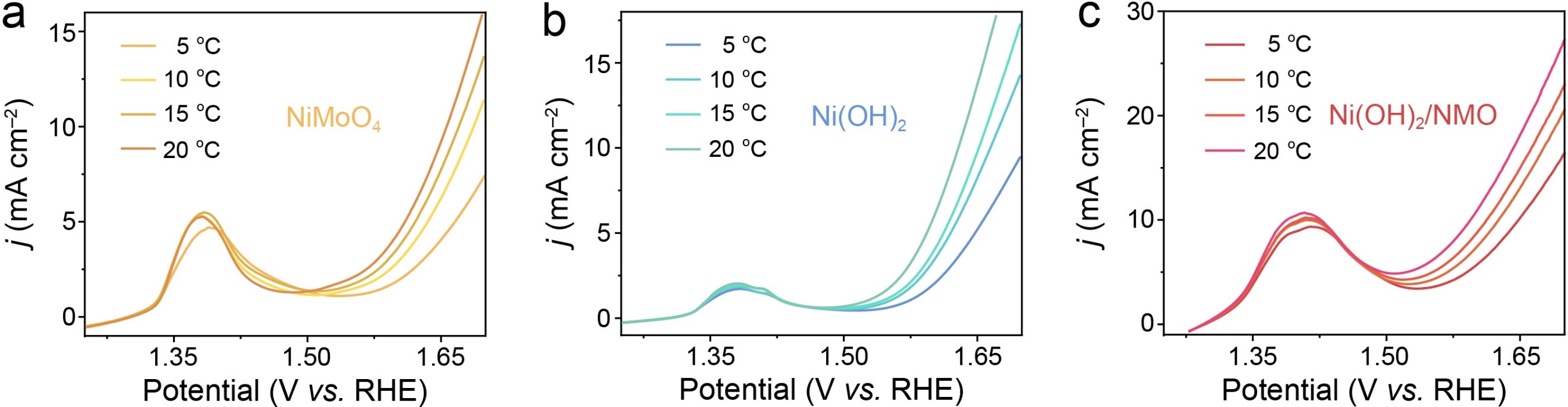


**Figure S27.** LSVs measured in 1 M KOH at various temperatures: (a) NiMoO_4_, (b) Ni(OH)_2_, and (c) Ni(OH)_2_/NMO.


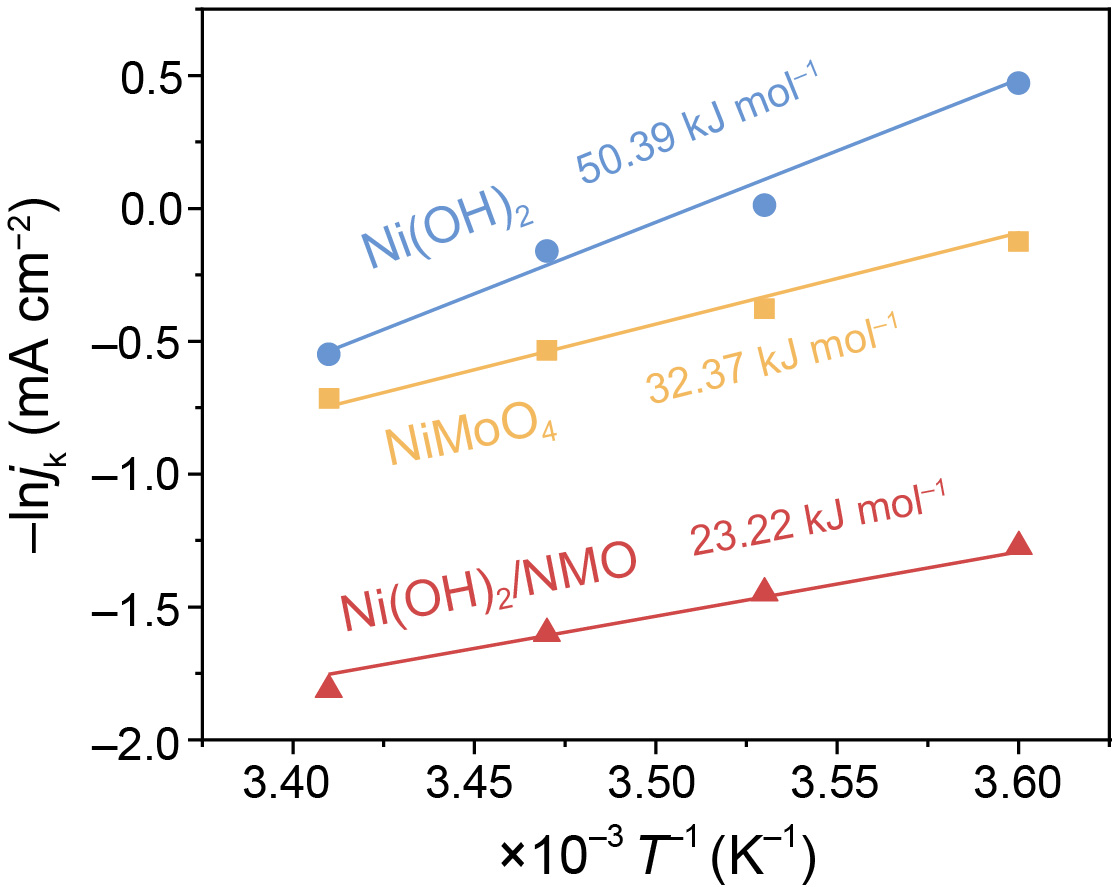


**Figure S28.** Arrhenius plots of Ni(OH)_2_, NiMoO_4_, and Ni(OH)_2_/NMO obtained using data from **Figure S27**.


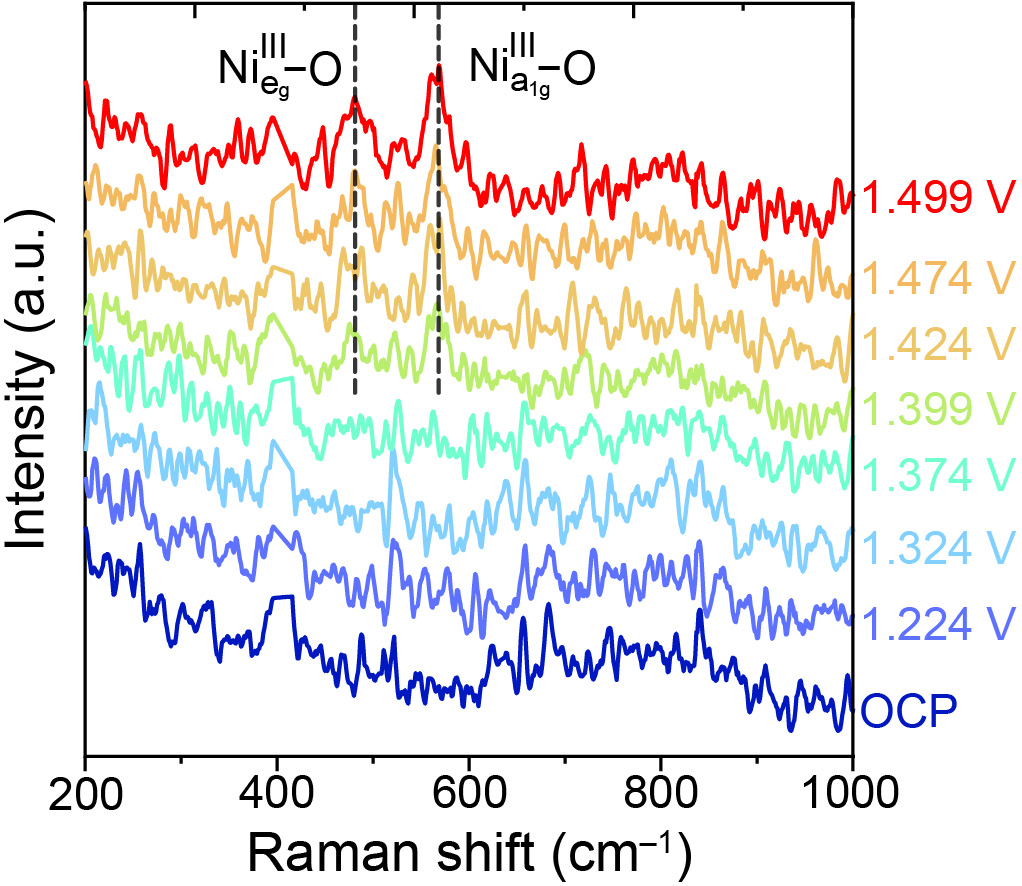


**Figure S29.** *In situ* Raman spectra of Ni(OH)_2_ in 1 M KOH.


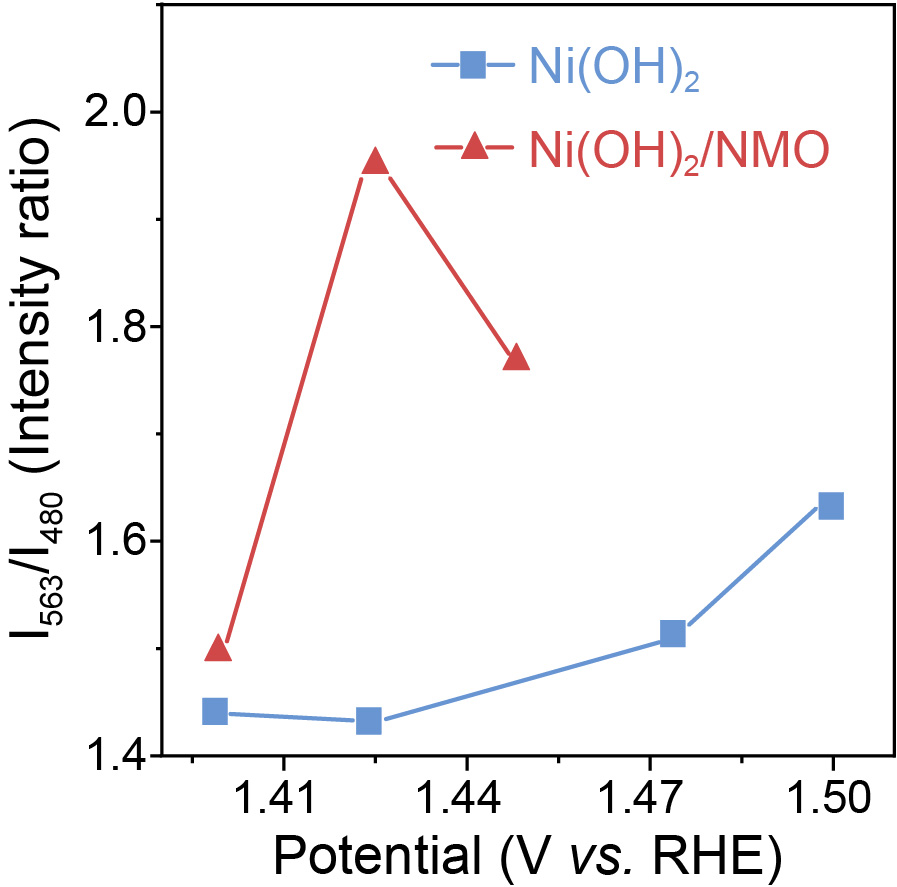


**Figure S30.** Intensity ratio of peak **ii**-to-**i** (I_563_/I_480_) at various applied potentials in 1 M KOH.


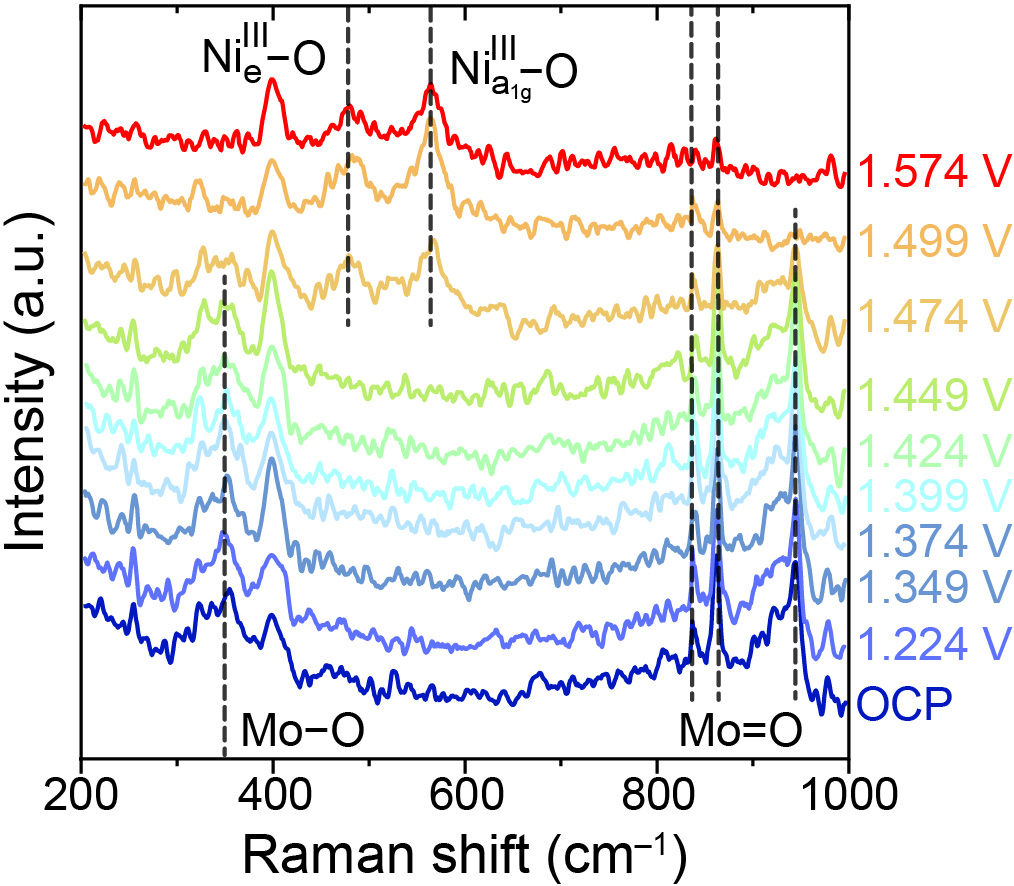


**Figure S31.** *In situ* Raman spectra of NiMoO_4_ in 1 M KOH.


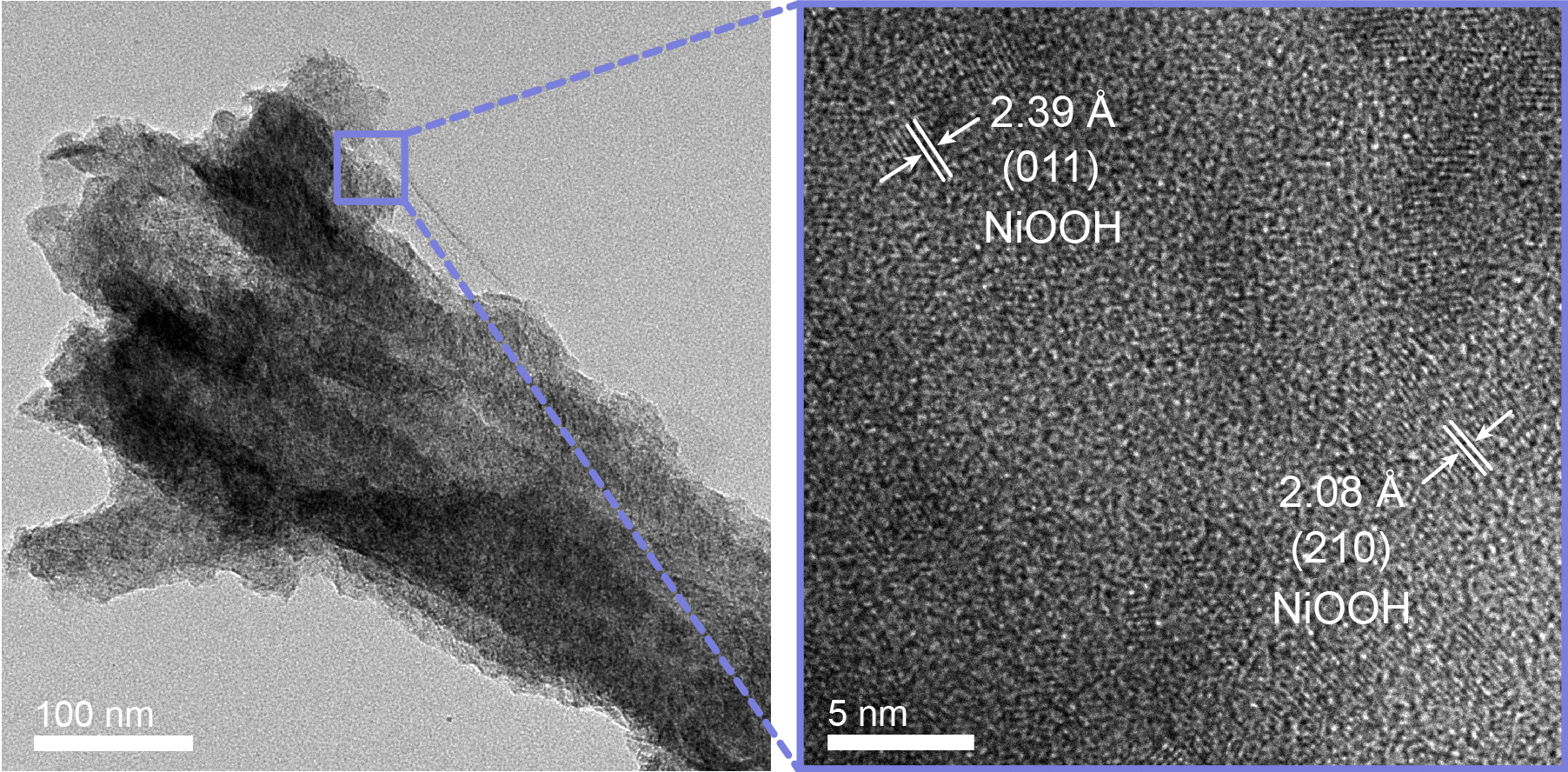


**Figure S32.** TEM and HRTEM images of Ni(OH)_2_/NMO after OER activation.


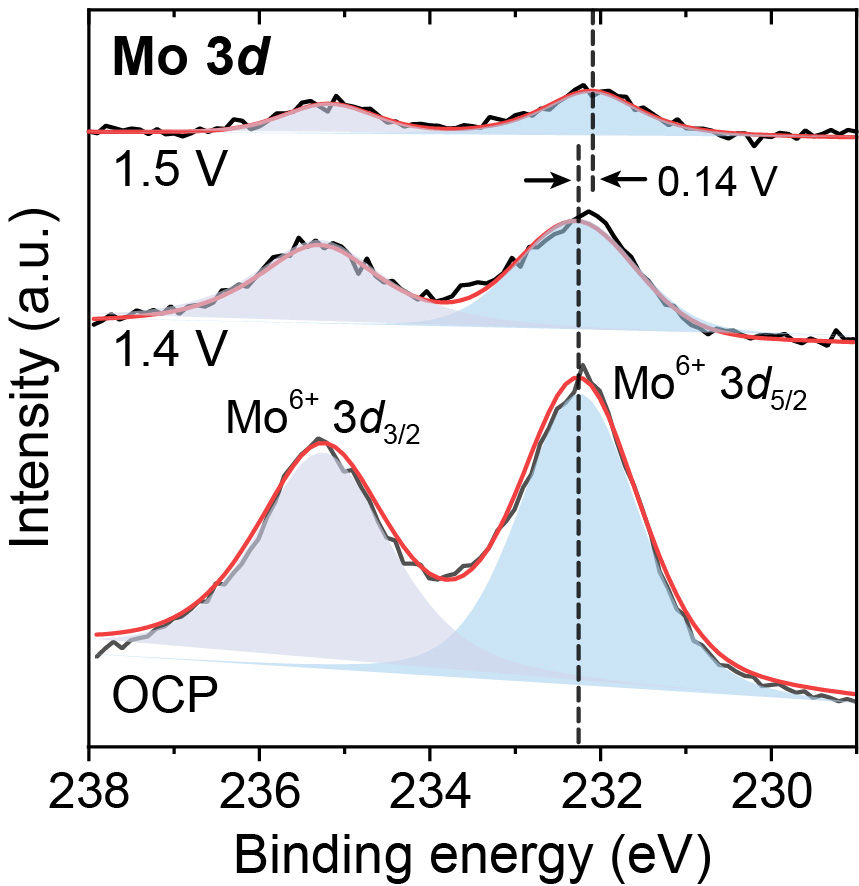


**Figure S33.** *Ex situ* XPS Mo 3*d* spectra of Ni(OH)_2_/NMO during OER activation.


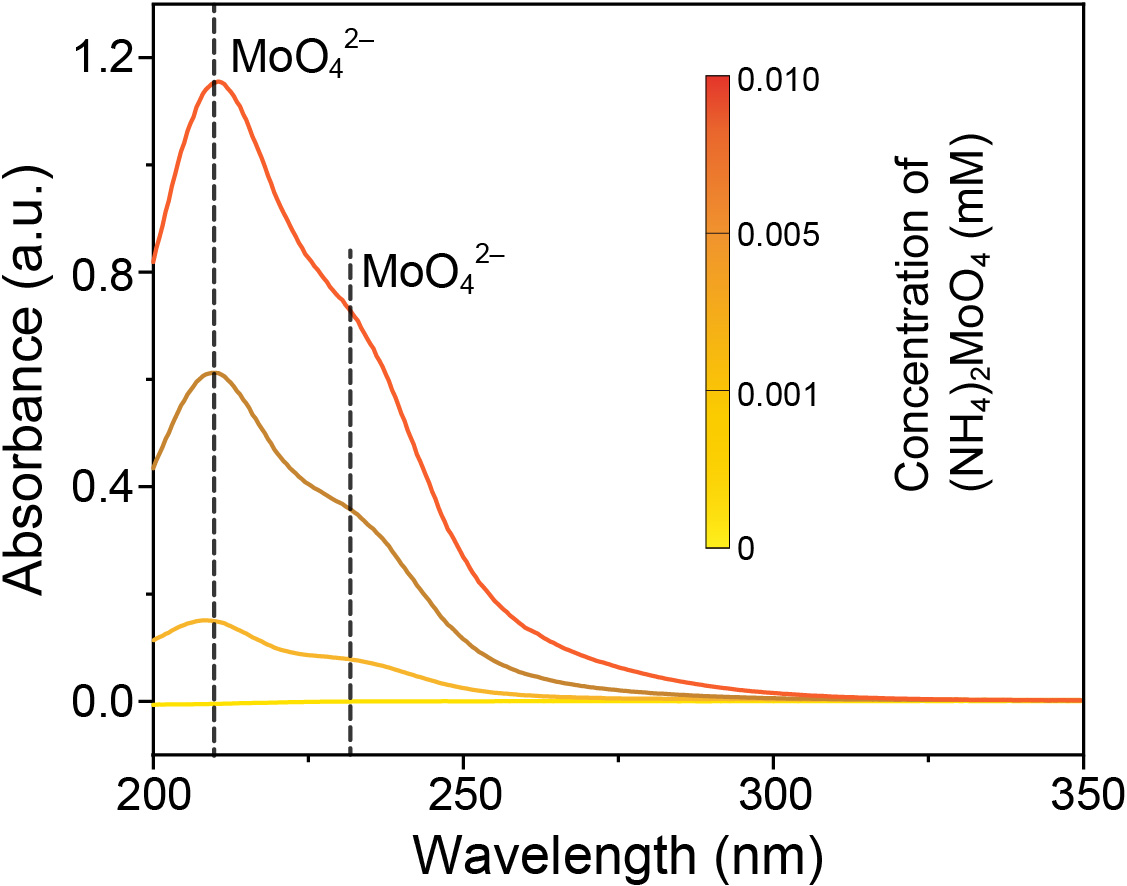


**Figure S34.** UV−Vis spectra of ammonium molybdate spectrophotometry with various (NH_4_)_2_MoO_4_ concentrations.


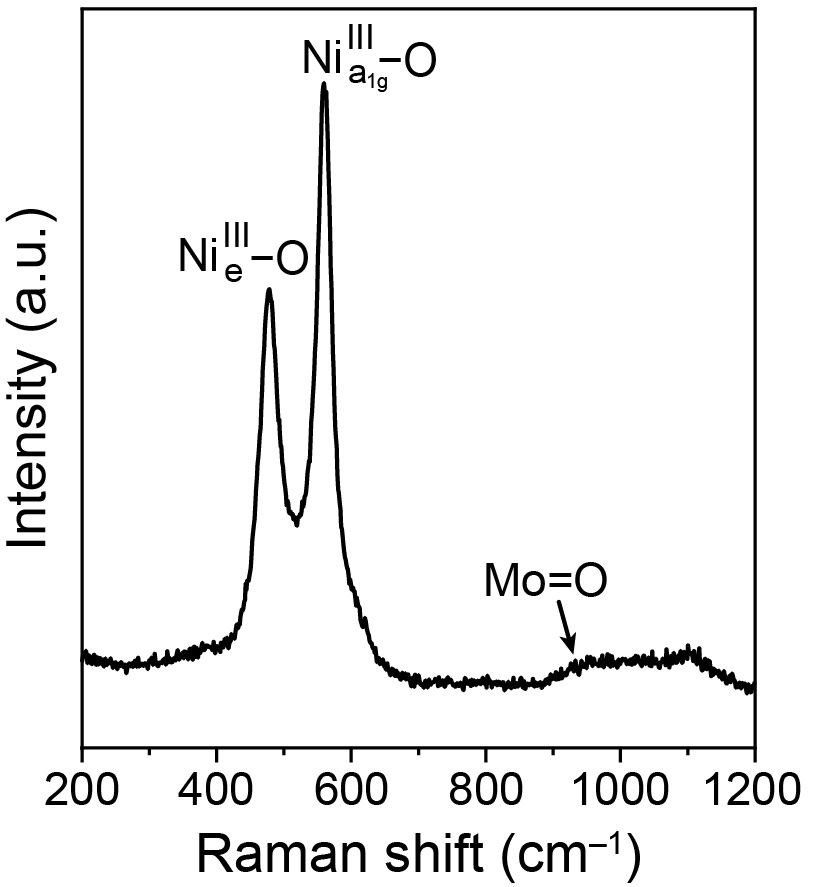


**Figure S35**. Raman spectrum of Ni(OH)_2_/NMO after 600-h *U*-t OER at 500 mA cm^−2^ in seawater + 1 M KOH.


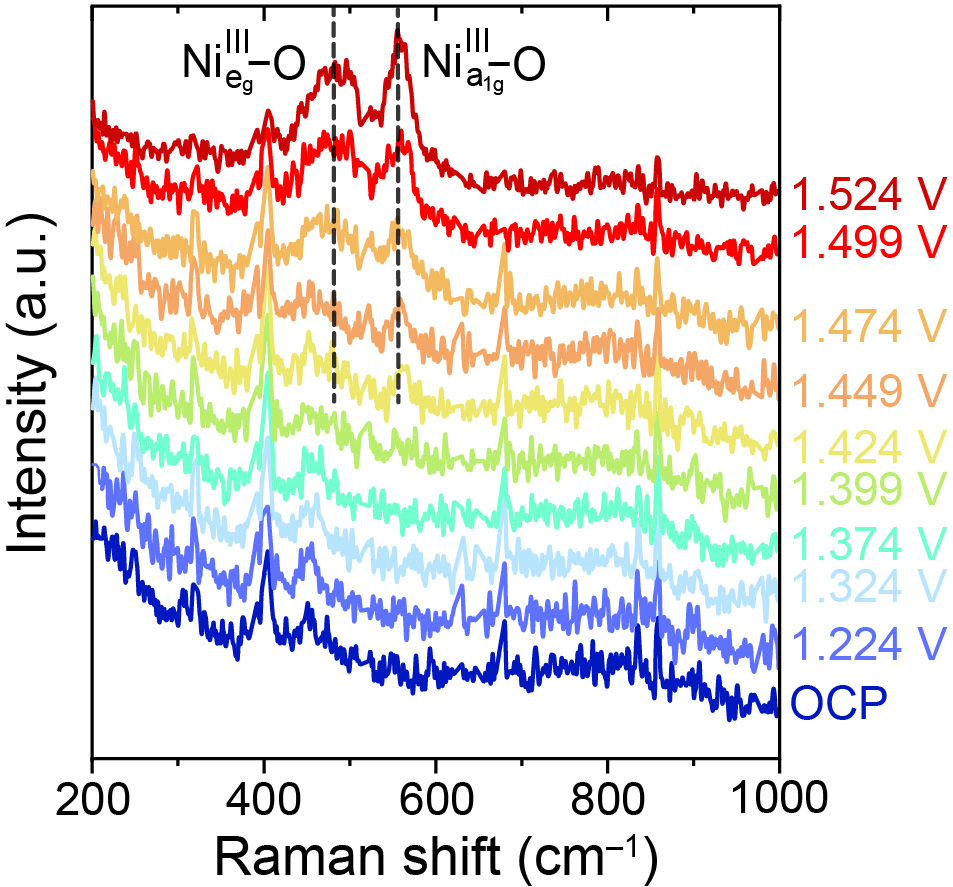


**Figure S36.** *In situ* Raman spectra of Ni(OH)_2_ in 1 M KOH + 0.1 M methanol.


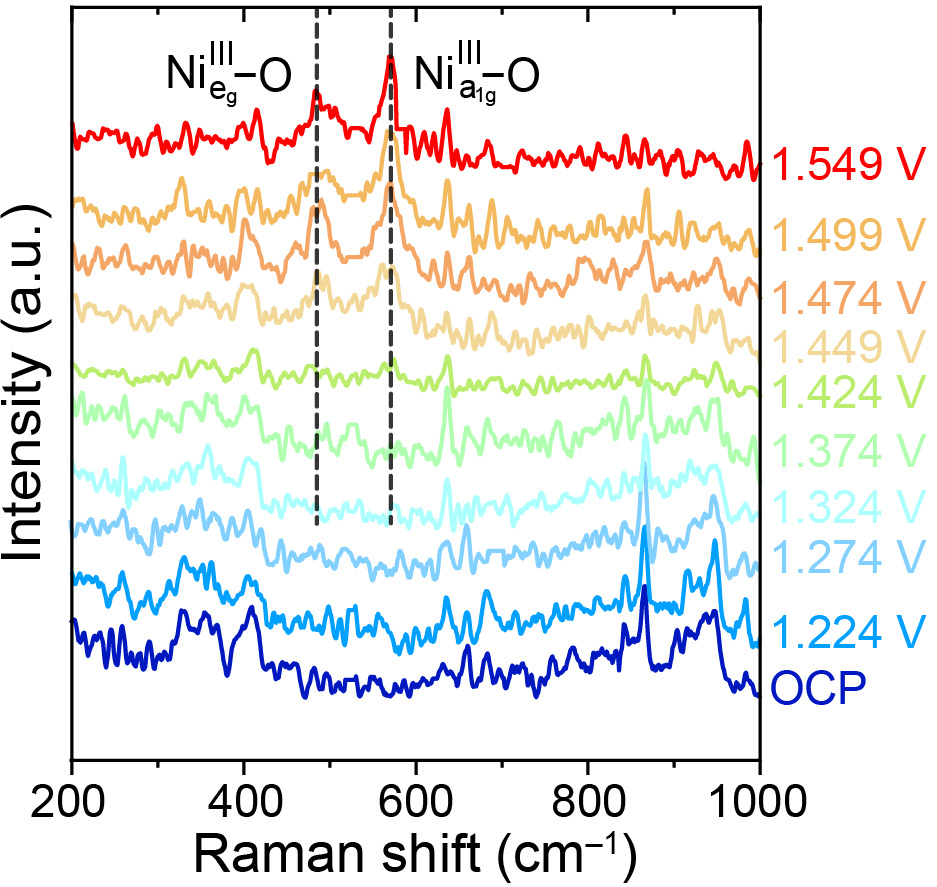


**Figure S37.** *In situ* Raman spectra of Ni(OH)_2_/NMO in 1 M KOH + 0.1 M methanol.


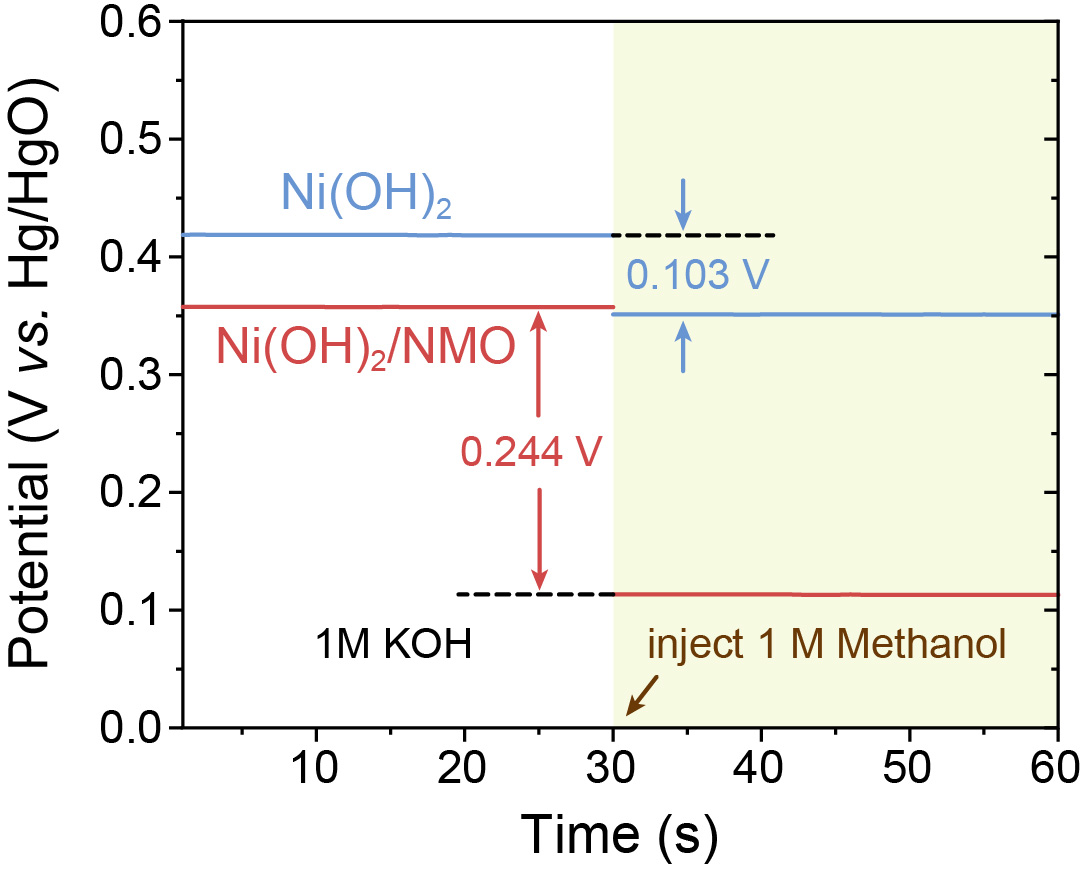


**Figure S38.** OCP curves of Ni(OH)_2_ and Ni(OH)_2_/NMO measured in 1 M KOH upon 1 M methanol injection.


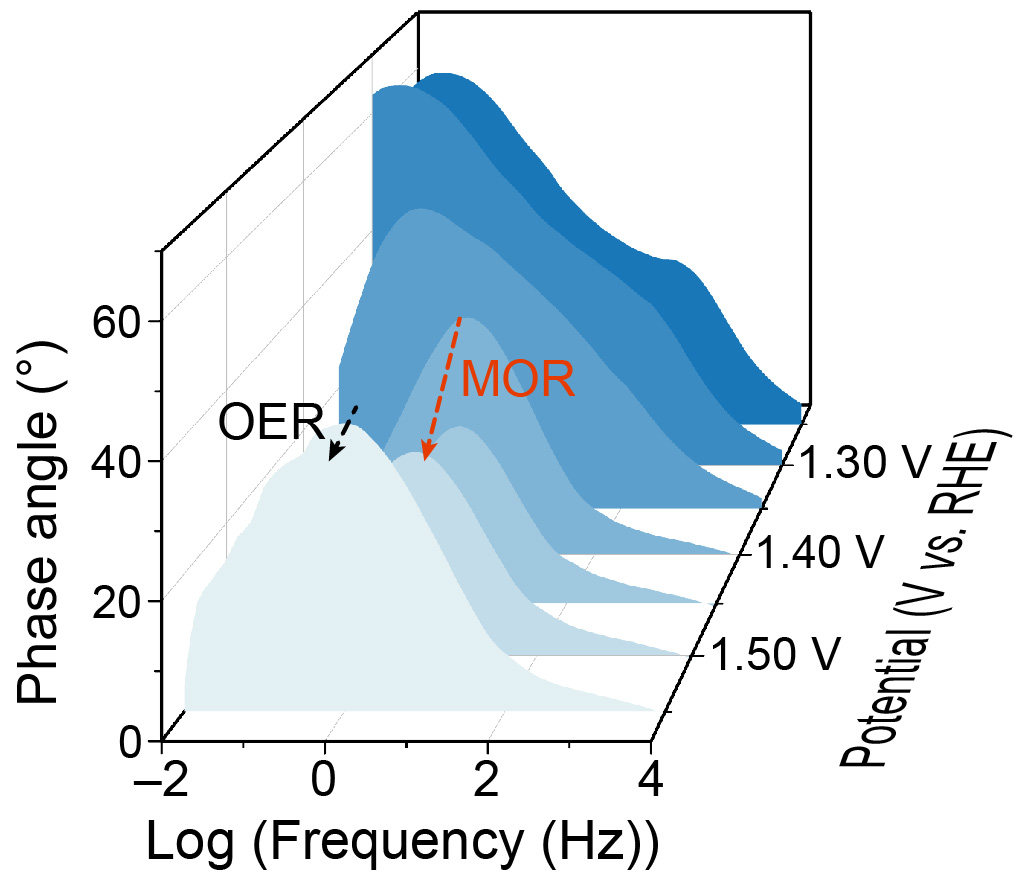


**Figure S39**. Bode phase plots for Ni(OH)_2_ obtained from *operando* EIS in 1 M KOH + 0.1 M methanol.


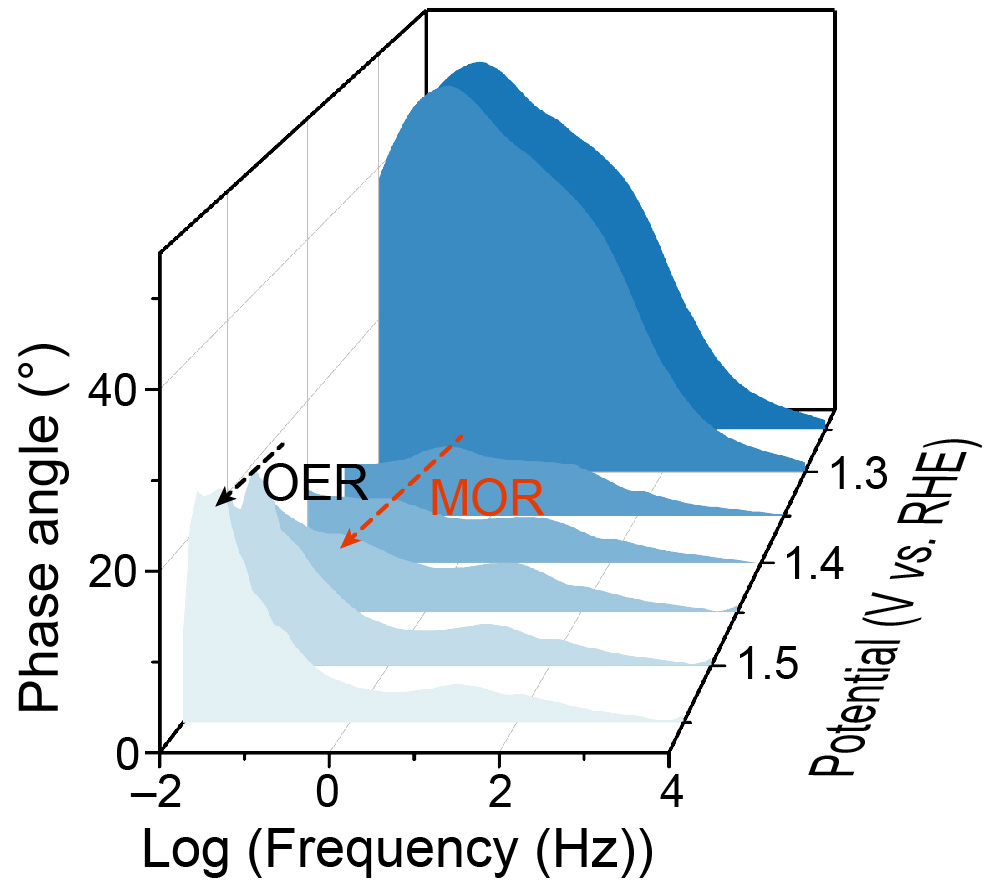


**Figure S40**. Bode phase plots for Ni(OH)_2_/NMO obtained from *operando* EIS in 1 M KOH + 0.1 M methanol.


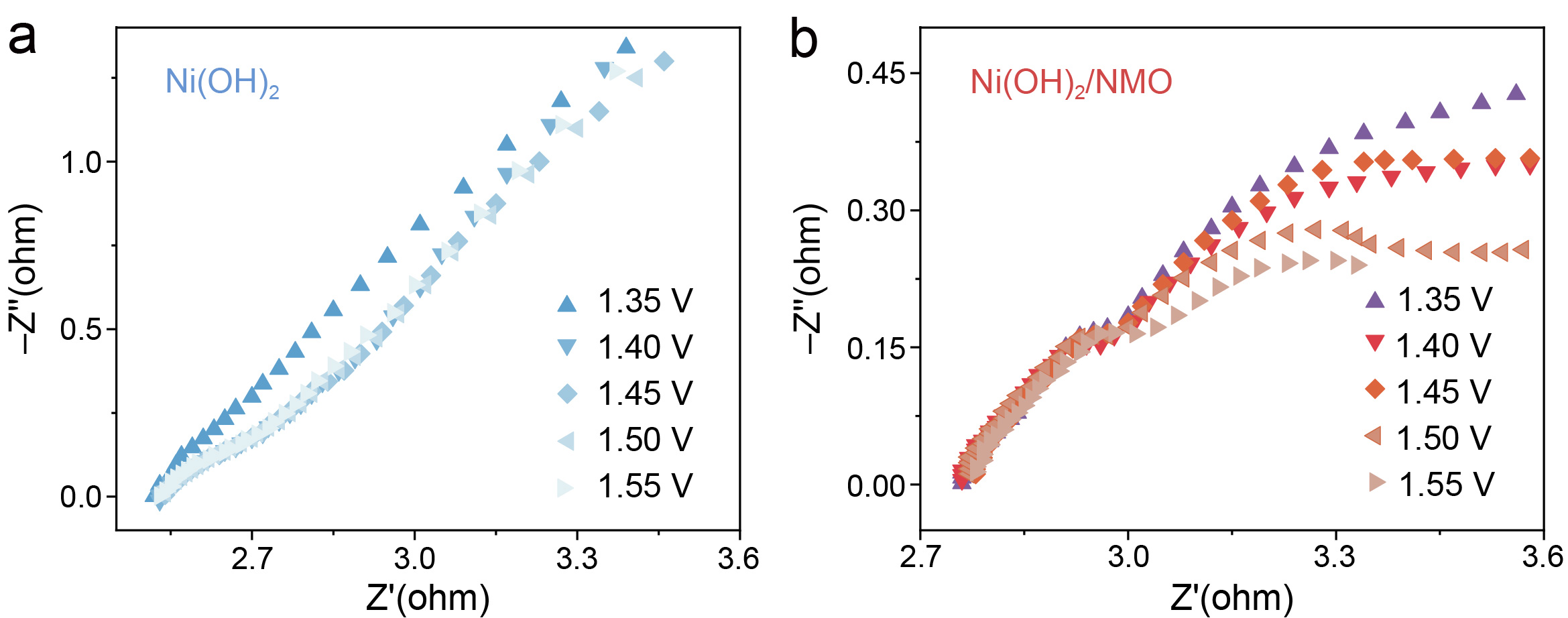


**Figure S41.** Nyquist plots of (a) Ni(OH)_2_ and (b) Ni(OH)_2_/NMO at various applied potentials in 1 M KOH.


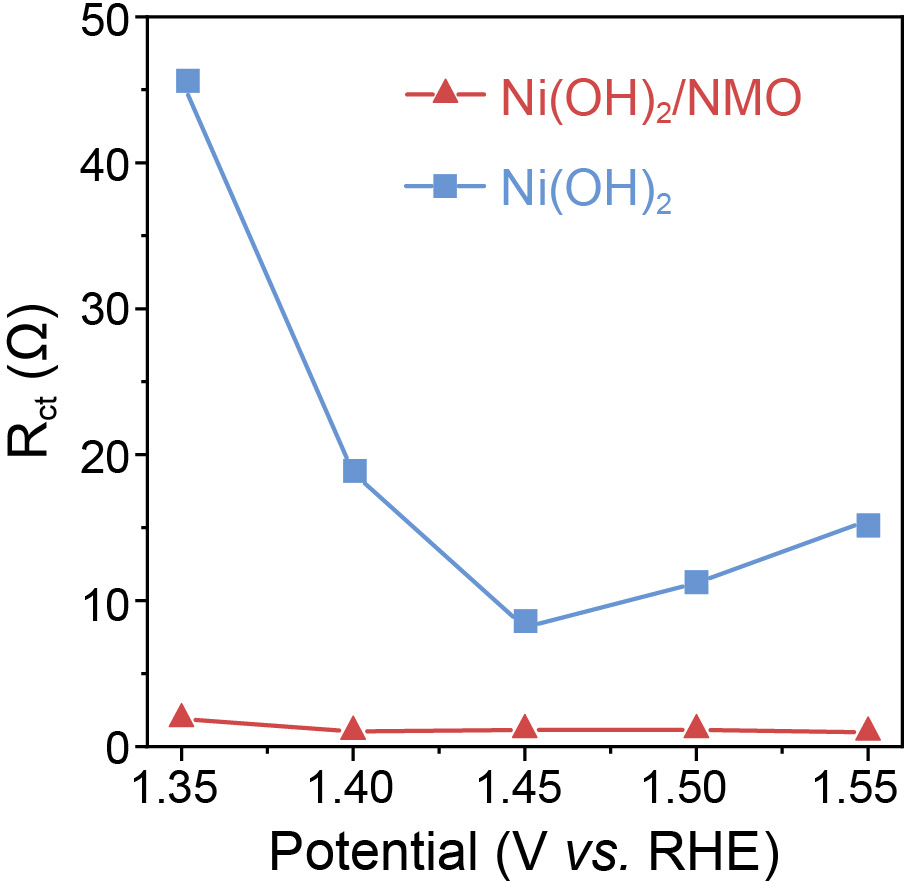


**Figure S42**. Solid–liquid interfacial resistances (R_ct_) at various applied potentials in 1 M KOH.


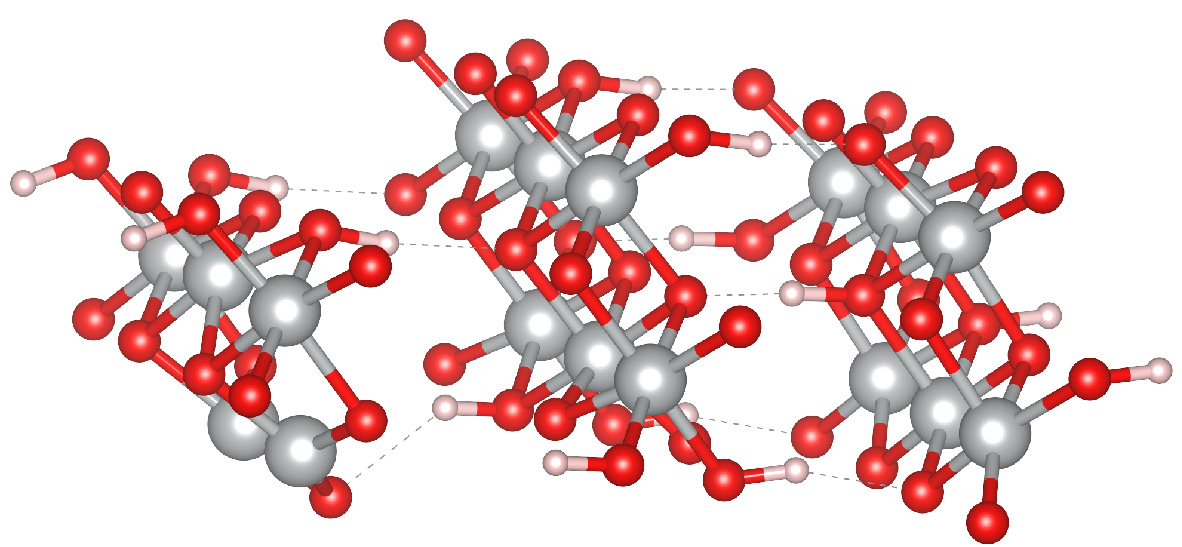


**Figure S43.** Optimized atomic structures of NiOOH. Ni, O, and H atoms are shown in gray, red, and pink, respectively.


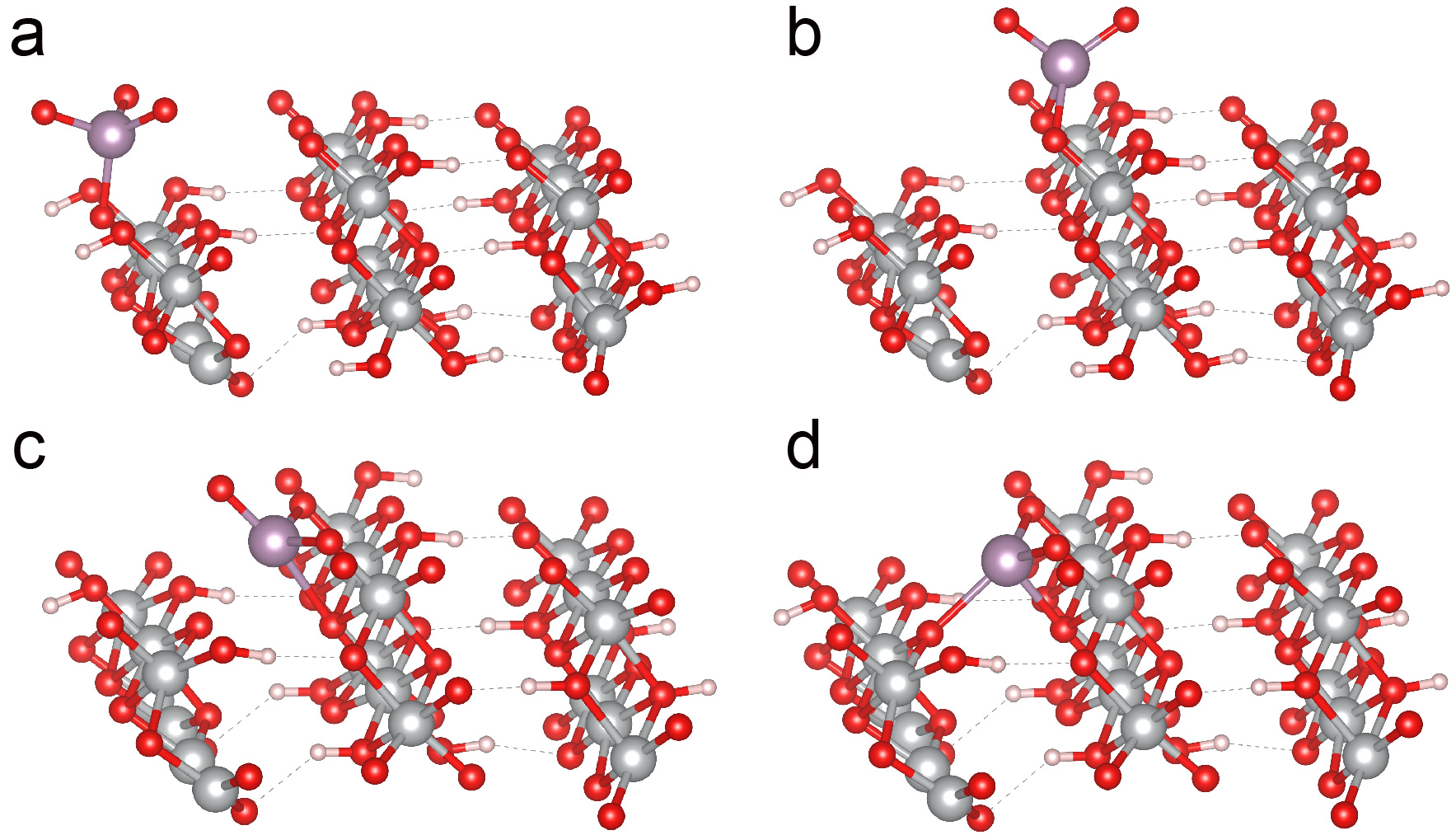


**Figure S44.** Various adsorption configurations of MoO_4_^2^^−^ on the O site in NiOOH. (a) Mono-oxygen, (b) di-oxygen, (c) tri-oxygen, and (d) tetra-oxygen coordinations. Ni, Mo, O, and H atoms are shown in gray, violet, red, and pink, respectively.


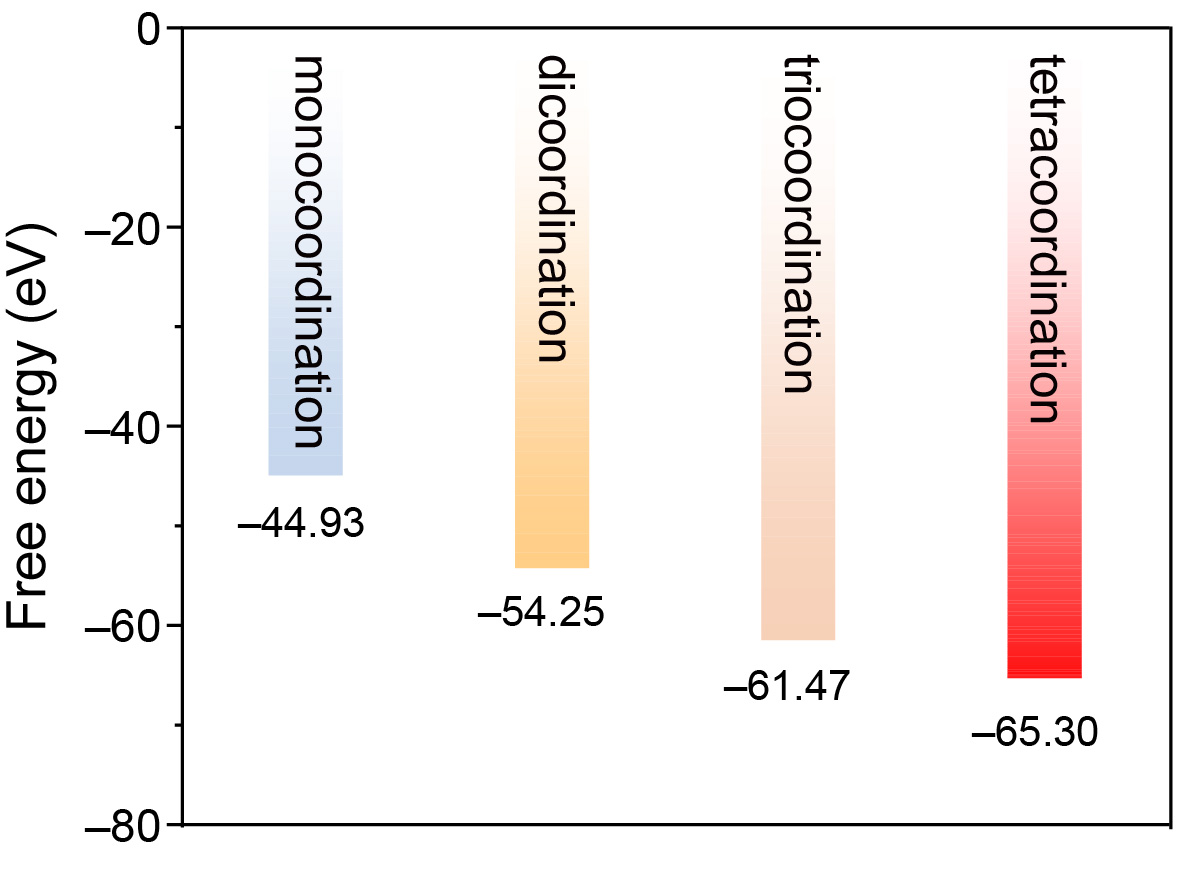


**Figure S45.** Adsorption free energies for MoO_4_^2−^ with different adsorption sites on NiOOH.


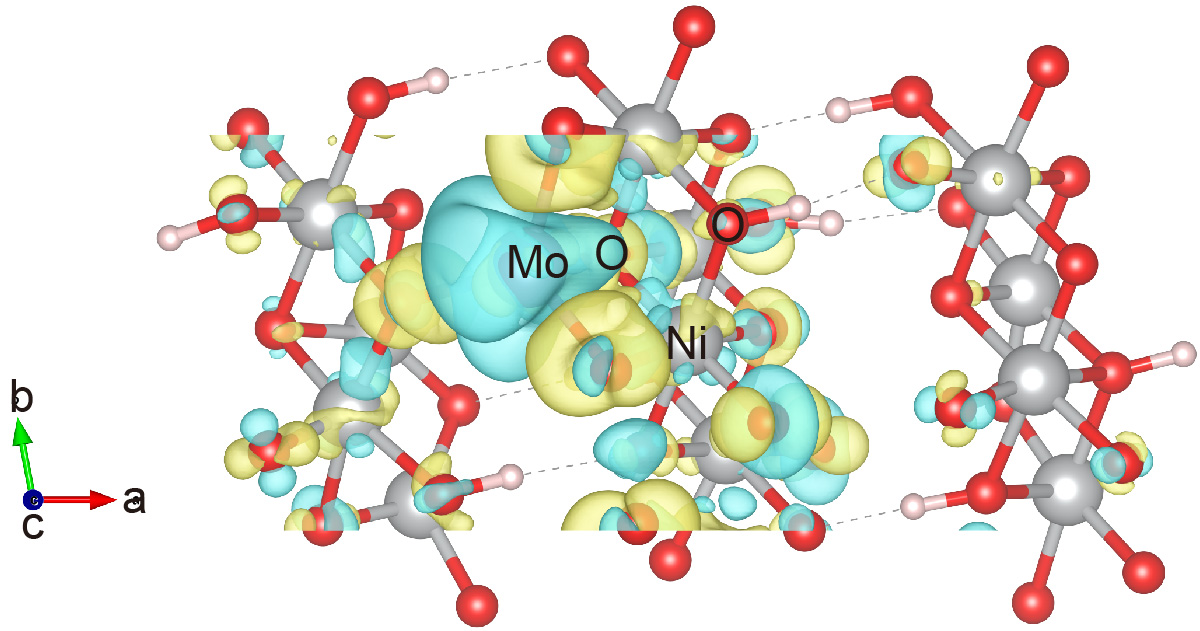


**Figure S46.** Charge density distribution in Mo-NiOOH. Yellow and blue regions represent electron accumulation and depletion, respectively.


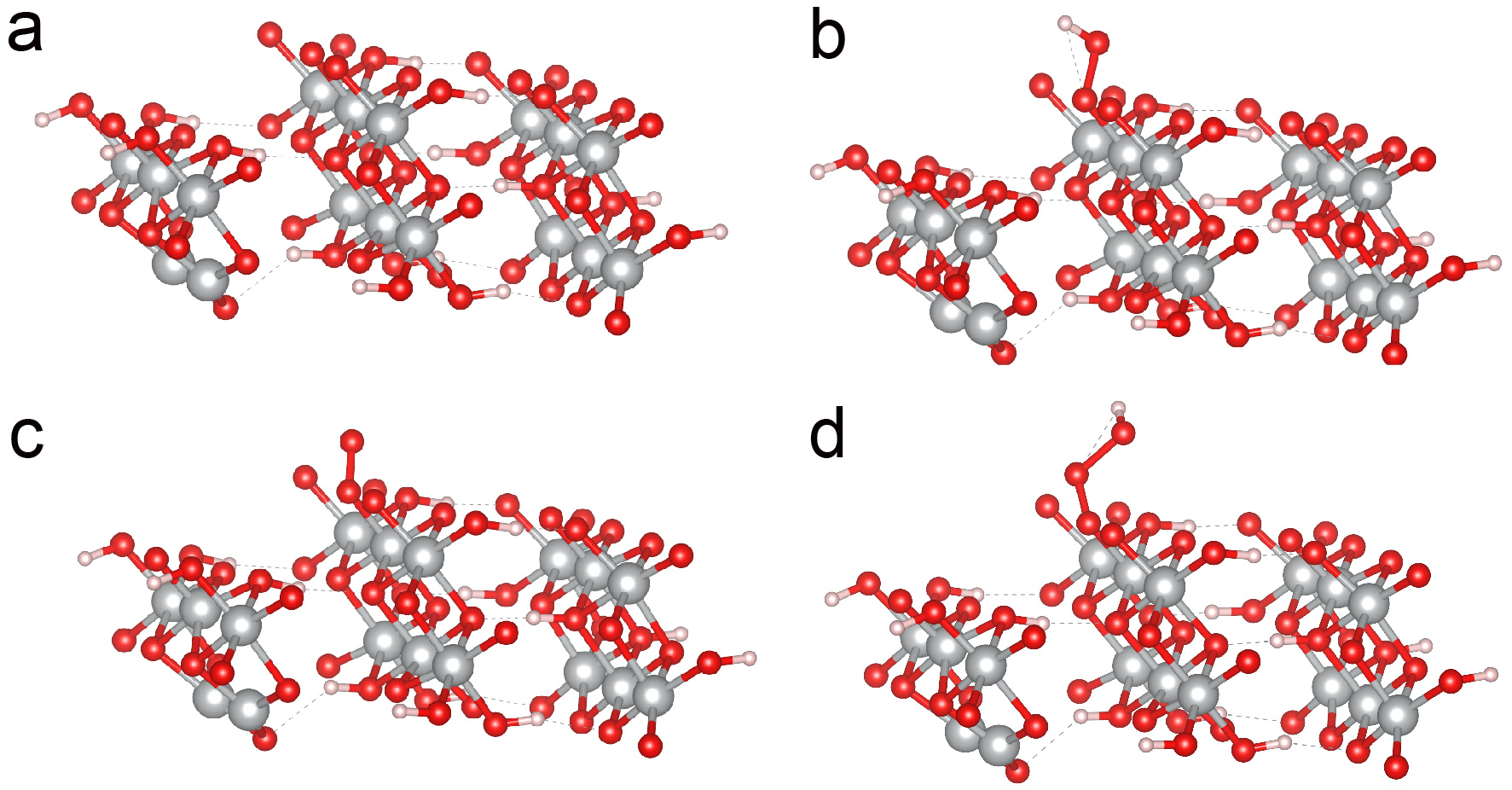


**Figure S47.** Structures of (a) bare NiOOH and (b) *OH, (c) *O, and (d) *OOH adsorbed NiOOH (O site). Ni, O, and H atoms are shown in gray, red, and pink, respectively.


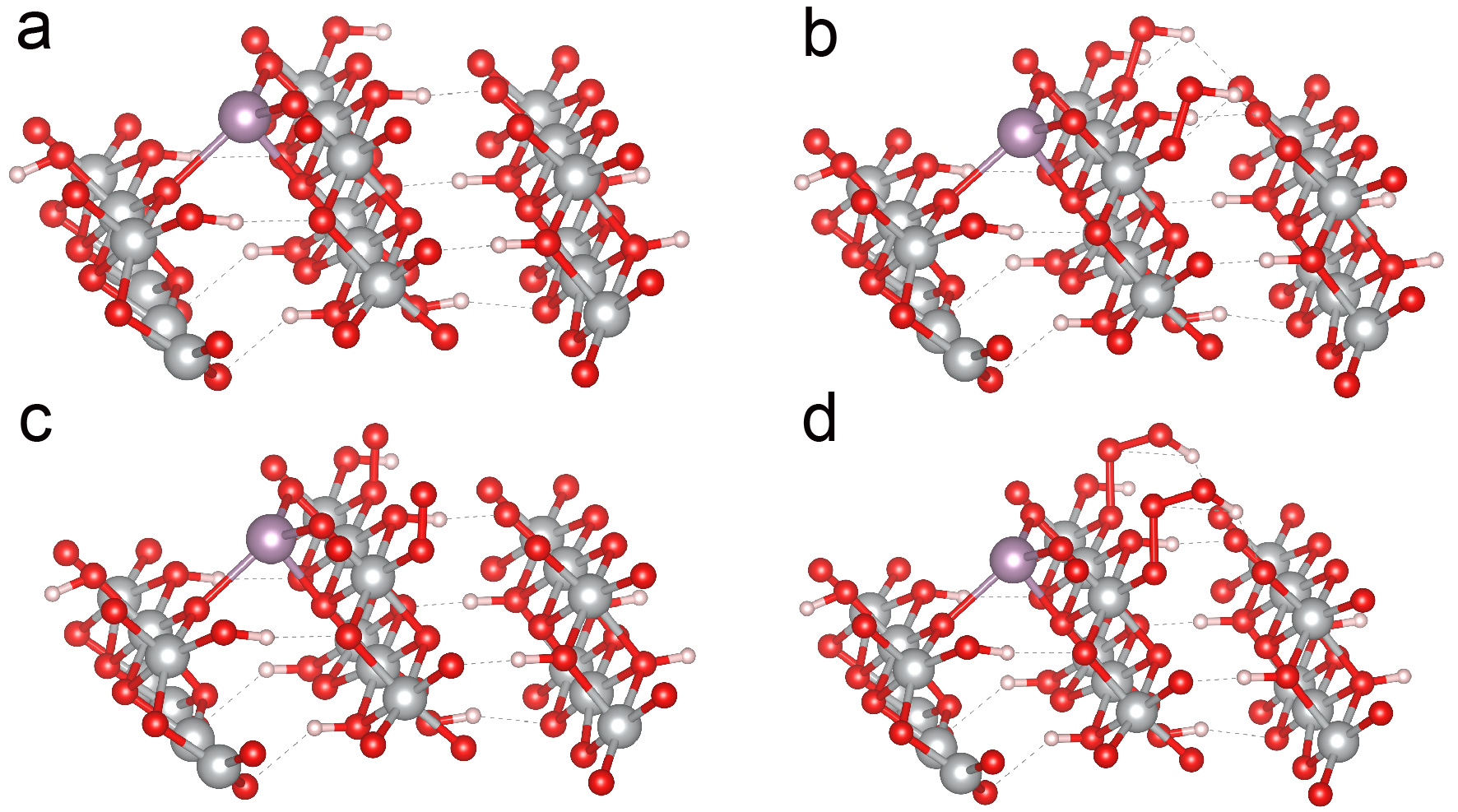


**Figure S48.** Structures of (a) bare Mo-NiOOH and (b) *OH, (c) *O, and (d) *OOH adsorbed Mo-NiOOH (O site). Ni, Mo, O, and H atoms are shown in gray, violet, red, and pink, respectively.


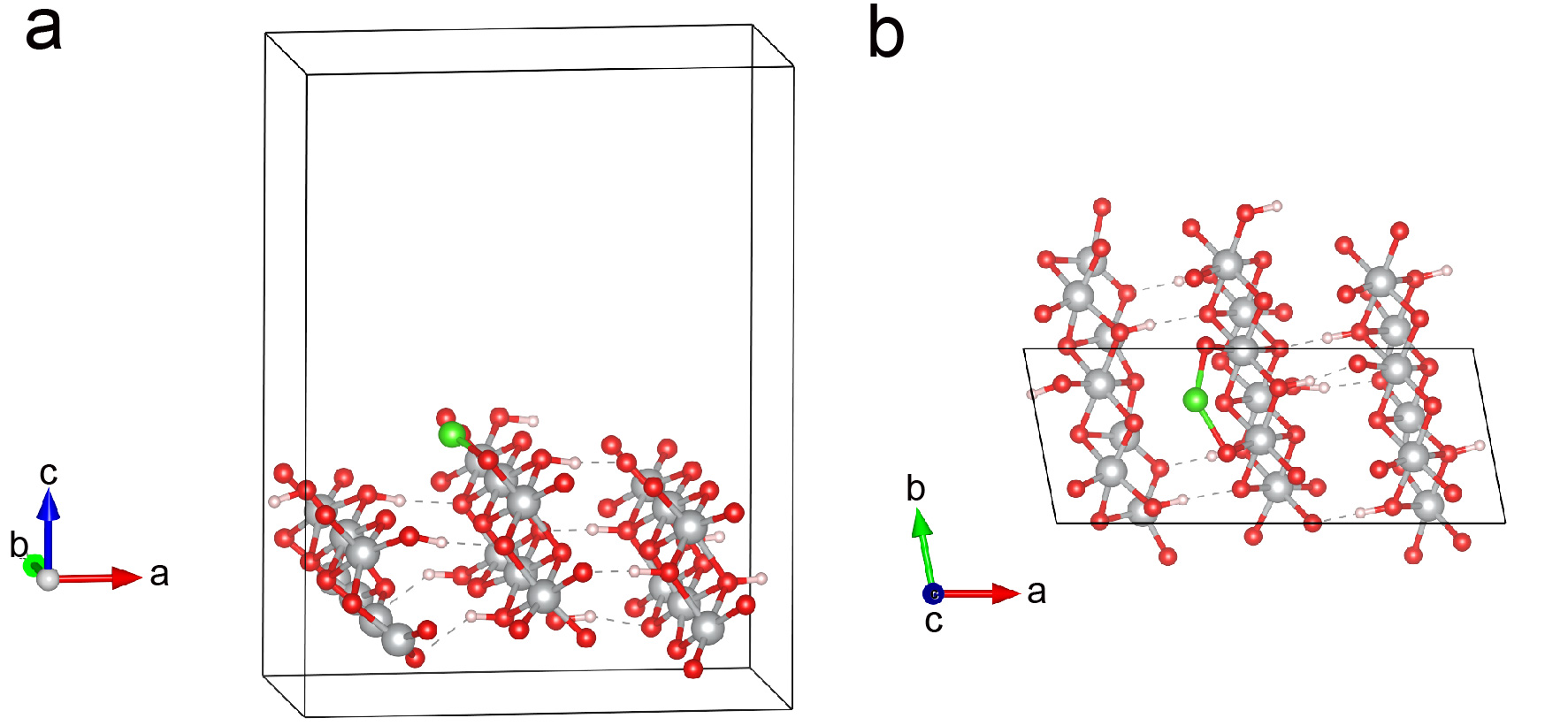


**Figure S49.** Atomic arrangement of *Cl-adsorbed NiOOH (O site): (a) side- and (b) top-views. Ni, O, Cl, and H atoms are shown in gray, red, green, and pink, respectively.


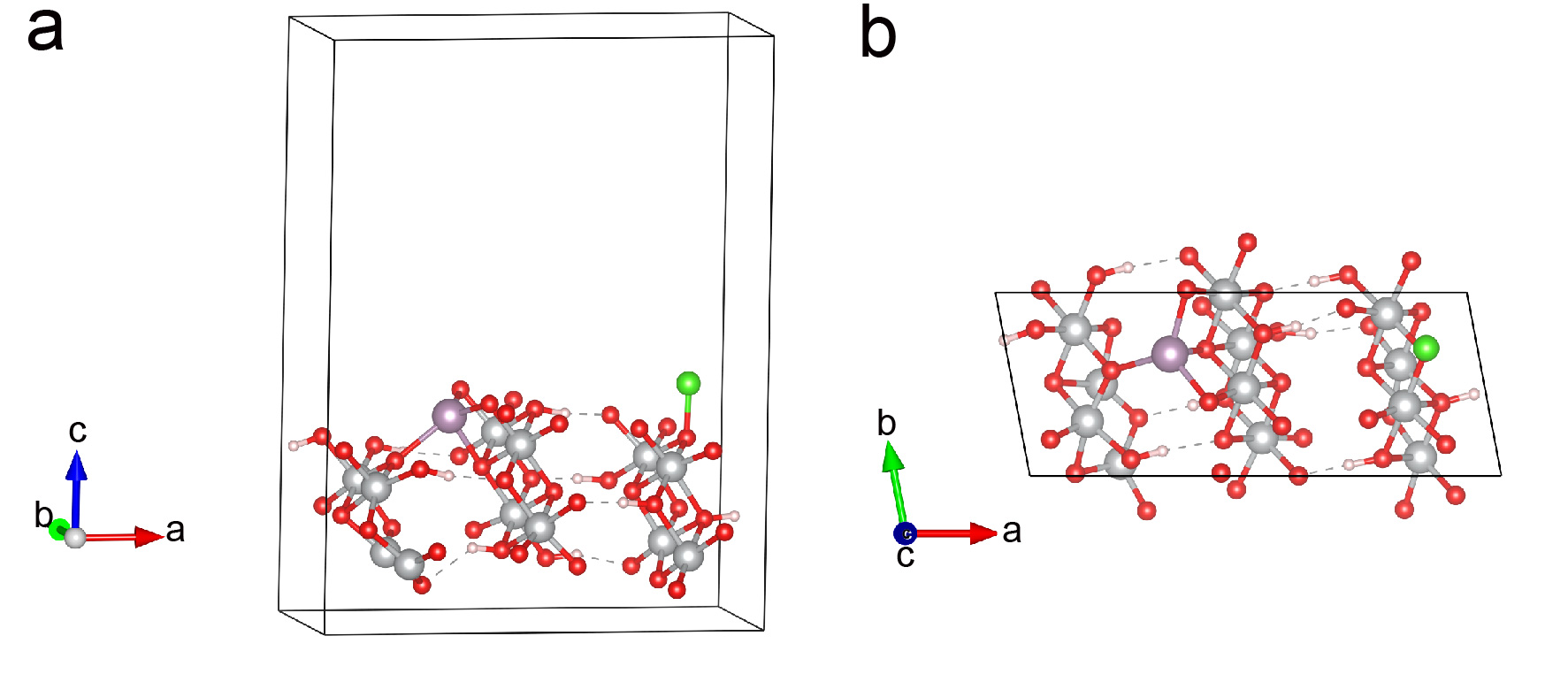


**Figure S50.** Structure of *Cl-adsorbed Mo-NiOOH (Mo site): (a) side- and (b) top-views. Ni, Mo, O, Cl, and H atoms are shown in gray, violet, red, green, and pink, respectively.


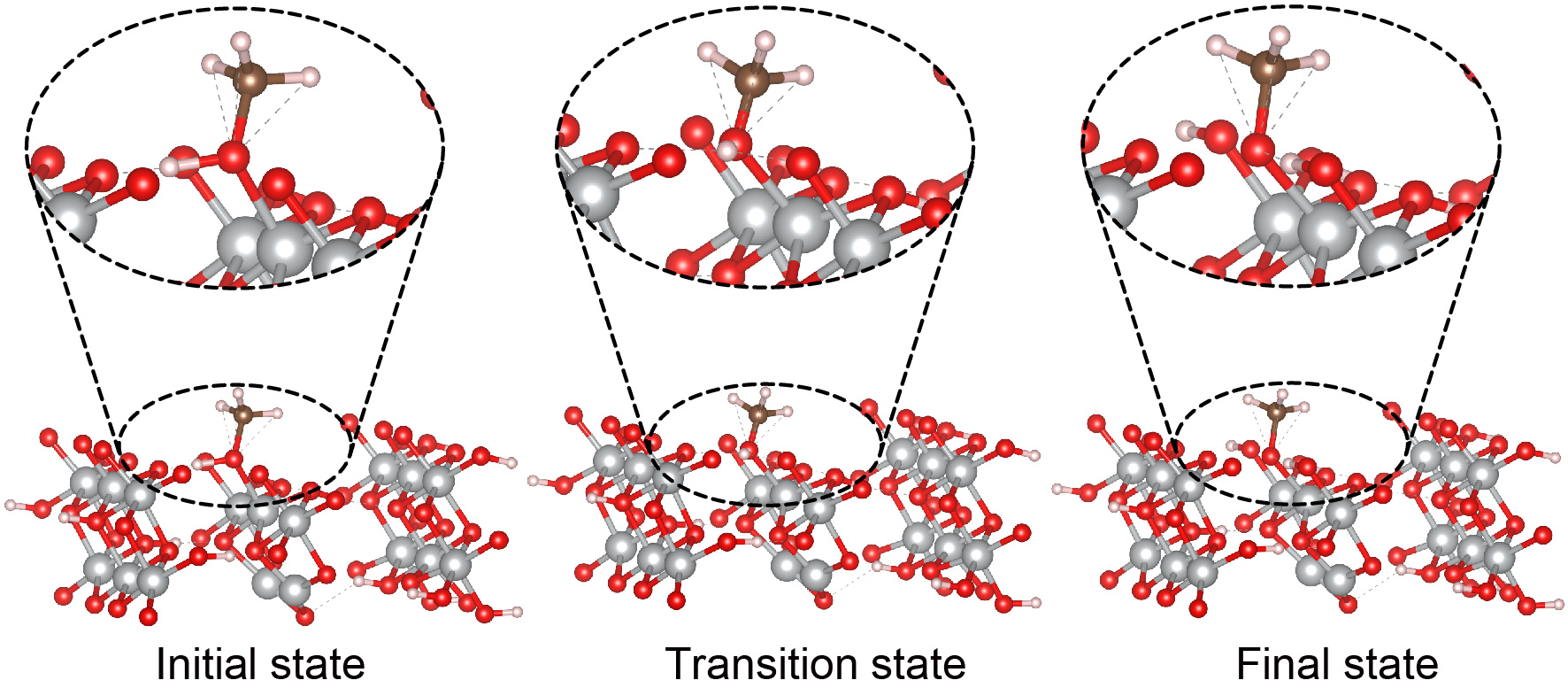


**Figure S51.** Side views of optimized methanol-adsorbed NiOOH (O site) during the hydrogen transfer process.


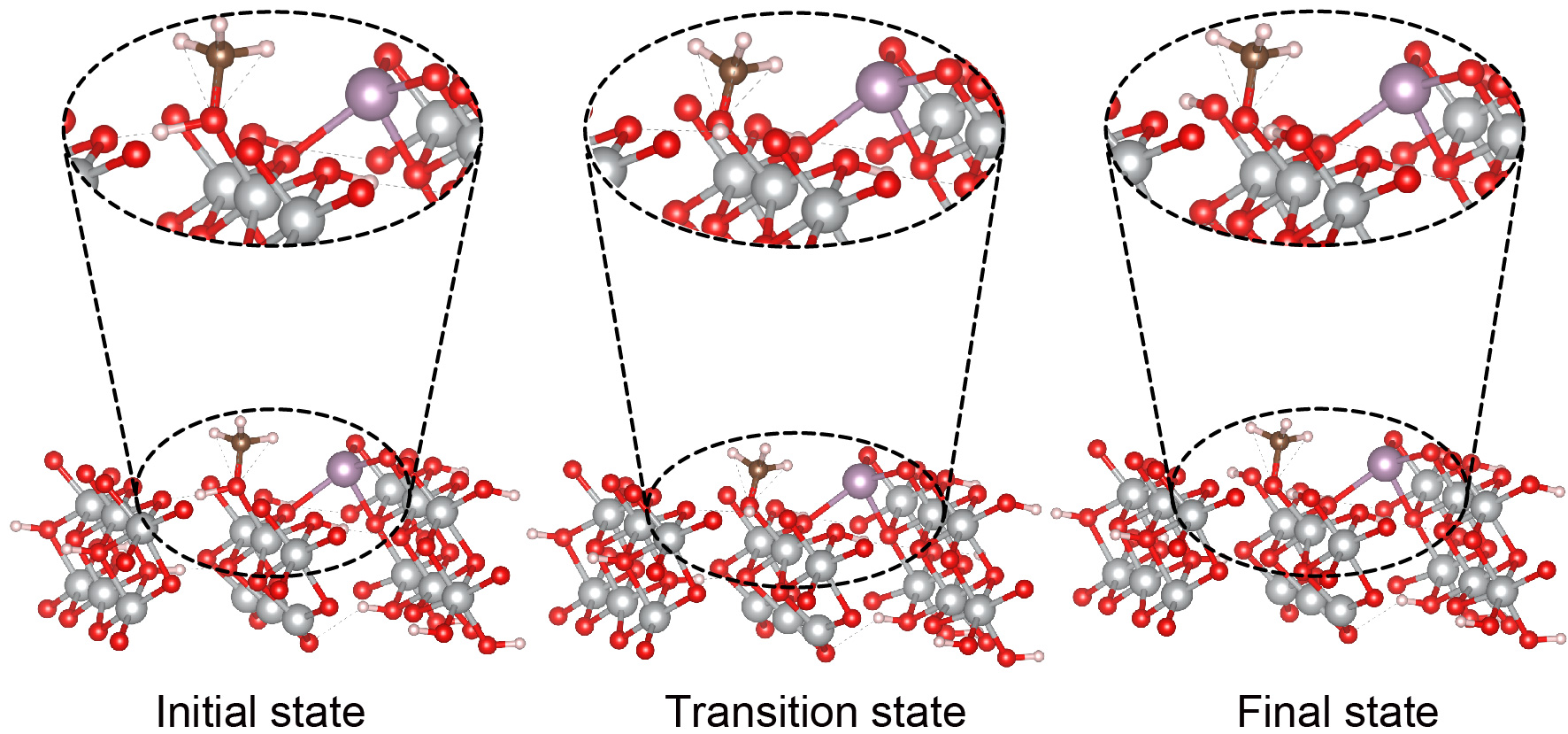


**Figure S52.** Side views of optimized methanol-adsorbed Mo-NiOOH (O site) during the hydrogen transfer process.

**Table S1.** ICP-OES analysis data of Ni(OH)_2_/NMO at various deposition stages.

| **Sample** | **Element content (ppm)** | | **Atomic ratio** |
| --- | --- | --- | --- |
|  | **Mo** | **Ni** | **Ni/(Ni+Mo)** |
| NiMoO_4_ | 97.33 | 65.80 | 0.52 |
| Ni(OH)_2_/NMO-5 min | 98.64 | 82.46 | 0.58 |
| Ni(OH)_2_/NMO-10 min | 94.47 | 101.62 | 0.64 |
| Ni(OH)_2_/NMO-15 min | 96.40 | 124.80 | 0.68 |
| Ni(OH)_2_ | 0 | 33.29 | 1 |

**Table S2.** Overpotentials of samples synthesized through electrodeposition with different loading times to reach 10 mA cm^−2^.

| Deposition time  (min) | Overpotential  (mV, at 10 mA cm^−2^) |
| --- | --- |
| 0 | 274 |
| 5 | 213 |
| 10 | 207 |
| 15 | 214 |

**Table S3.** EIS Parameters for samples synthesized using various electrodeposition times.

| Deposition time  (min) | R_s_ (Ω) | R_ct_ (Ω) | R_2_ (Ω) | CPE_1_ (F) | CPE_2_ (F) |
| --- | --- | --- | --- | --- | --- |
| 0 | 2.34 | 20.52 | 0.66 | 0.49 | 0.13 |
| 5 | 2.33 | 5.28 | 0.17 | 1.65 | 0.93 |
| 10 | 2.33 | 3.04 | 0.15 | 0.67 | 2.04 |
| 15 | 2.32 | 5.49 | 0.28 | 0.01 | 5.29 |

**Table S4.** Comparison of electrocatalytic performance of Ni(OH)_2_/NMO in 1 M KOH + seawater with previously reported materials.

| Samples | Overpotential  (mV, at 100 mA cm^−2^) | Overpotential  (mV, at 500 mA cm^−2^) | Ref. |
| --- | --- | --- | --- |
| Ni(OH)_2_/NMO | 267 | 381 | **this work** |
| B-Co_2_Fe LDH | 310 | 376 | [12] |
| NiFe-CuCo LDH | 315 | 355 | [13] |
| S-(Ni,Fe)OOH | 300 | 398 | [14] |
| CoFe-Ni_2_P | 274 | 360 | [15] |

**Table S5.** Cell potentials and estimated energy consumption for H_2_ production using Pt/C‖RuO_2_ and NiMo‖Ni(OH)_2_/NMO as electrode materials in methanol-contained alkaline seawater.

| **Current density**  **(A cm^−2^)** | **Pt/C‖RuO_2_** | | **NiMo‖Ni(OH)_2_/NMO** | |
| --- | --- | --- | --- | --- |
|  | **Potential**  **(V)** | **Energy**  **(kJ mol^−1^ H_2_)** | **Potential**  **(V)** | **Energy**  **(kJ mol^−1^ H_2_)** |
| 0.1 | 2.126 | 205.13 | 1.840 | 177.50 |
| 0.2 | 2.341 | 225.87 | 2.018 | 194.71 |
| 0.3 | 2.485 | 239.77 | 2.143 | 206.77 |
| 0.4 | 2.602 | 251.05 | 2.241 | 216.22 |
| 0.5 | 2.709 | 261.38 | 2.324 | 224.23 |

**Table S6.** Resistances of catalyst/electrolyte interfaces at various applied potentials.

| **Potential (V)** | **Resistance (Ω)** | |
| --- | --- | --- |
|  | **Ni(OH)_2_** | **Ni(OH)_2_/NMO** |
| 1.35 | 45.67 | 1.90 |
| 1.40 | 18.74 | 1.04 |
| 1.45 | 8.17 | 1.14 |
| 1.50 | 11.20 | 1.15 |
| 1.55 | 15.49 | 0.98 |

**References**

[1] M. Fang, W. Gao, G. F. Dong, Z. M. Xia, S. Yip, Y. B. Qin, Y. Q. Qu, J. C. Ho, *Nano Energy* **2016**, *27*, 247.

[2] G. Kresse, J. Furthmuller, *Phys. Rev. B* **1996**, *54*, 11169.

[3] G. Kresse, J. Hafner, *Phys. Rev. B* **1994**, *49*, 14251.

[4] G. Kresse, J. Hafner, *Phys. Rev. B* **1993**, *47*, 558.

[5] G. Kresse, D. Joubert, *Phys. Rev. B* **1999**, *59*, 1758.

[6] P. E. Blochl, *Phys. Rev. B* **1994**, *50*, 17953.

[7] J. P. Perdew, K. Burke, M. Ernzerhof, *Phys. Rev. Lett.* **1997**, *78*, 1396.

[8] H. J. Monkhorst, J. D. Pack, *Phys. Rev. B* **1976**, *13*, 5188.

[9] W. Tang, E. Sanville, G. Henkelman, *J. Phys.: Condens. Matter* **2009**, *21*, 084204.

[10] E. Sanville, S. D. Kenny, R. Smith, G. Henkelman, *J. Comput. Chem.* **2007**, *28*, 899.

[11] G. Henkelman, A. Arnaldsson, H. Jónsson, *Comput. Mater. Sci.* **2006**, *36*, 354.

[12] L. B. Wu, L. Yu, Q. C. Zhu, B. McElhenny, F. H. Zhang, C. Z. Wu, X. X. Xing, J. M. Bao, S. Chen, Z. F. Ren, *Nano Energy* **2021**, *83*, 105838.

[13] L. Yu, J. Y. Xiao, C. Q. Huang, J. Q. Zhou, M. Qiu, Y. Yu, Z. F. Ren, C. W. Chu, J. C. Yu, *Proc. Natl. Acad. Sci. USA* **2022**, *119*, e2202382119.

[14] L. Yu, L. B. Wu, B. McElhenny, S. W. Song, D. Luo, F. H. Zhang, Y. Yu, S. Chen, Z. F. Ren, *Energy Environ. Sci.* **2020**, *13*, 3439.

[15] C. Q. Huang, Q. C. Zhou, L. Yu, D. S. Duan, T. Y. Cao, S. H. Qiu, Z. Z. Wang, J. Guo, Y. X. Xie, L. P. Li, Y. Yu, *Adv. Energy Mater.* **2023**, *13*, 2301475.
